# Right-sided brain lesions predominate among patients with lesional mania: evidence from a systematic review and pooled lesion analysis

**DOI:** 10.1038/s41398-020-0811-0

**Published:** 2020-05-12

**Authors:** J. Bernardo Barahona-Corrêa, Gonçalo Cotovio, Rui M. Costa, Ricardo Ribeiro, Ana Velosa, Vera Cruz e. Silva, Christoph Sperber, Hans-Otto Karnath, Suhan Senova, Albino J. Oliveira-Maia

**Affiliations:** 1 Champalimaud Clinical Centre, Champalimaud Centre for the Unknown, Av. Brasilia, 1400-038 Lisboa, Portugal; 2 Champalimaud Research, Champalimaud Centre for the Unknown, Av. Brasilia, 1400-038 Lisboa, Portugal; 3 Department of Psychiatry and Mental Health, Centro Hospitalar de Lisboa Ocidental, Rua da Junqueira 126, 1340-019 Lisboa, Portugal; 4 NOVA Medical School | Faculdade de Ciências Médicas, Universidade Nova de Lisboa, Campo Mártires da Pátria 130, 1169-056 Lisboa, Portugal; 5 Department of Neuroscience, Zuckerman Mind Brain Behavior Institute, Columbia University, New York, NY 10027 USA; 6 Department of Neuroradiology, Centro Hospitalar de Lisboa Ocidental, Rua da Junqueira 126, 1340-019 Lisboa, Portugal; 7 Center of Neurology, Division of Neuropsychology, Hertie-Institute for Clinical Brain Research, University of Tübingen, Tübingen, Germany; 8 Department of Psychology, University of South Carolina, Columbia, SC USA; 9 Neurosurgery and PePsy Departments, Assistance Publique-Hôpitaux de Paris (APHP), Groupe Henri-Mondor Albert-Chenevier, Créteil, France; 10 Equipe 14, U955 INSERM, Institut Mondor de Recherche Biomedicale and Faculté de Médecine, Université Paris Est, Créteil, France; 11 Present Address: Department of Neuroradiology, Hospital de Braga, Sete Fontes – São Victor, 4710-243 Braga, Portugal

**Keywords:** Prognostic markers, Bipolar disorder, Diagnostic markers, Human behaviour, Predictive markers

## Abstract

Despite claims that lesional mania is associated with right-hemisphere lesions, supporting evidence is scarce, and association with specific brain areas has not been demonstrated. Here, we aimed to test whether focal brain lesions in lesional mania are more often right- than left-sided, and if lesions converge on areas relevant to mood regulation. We thus performed a systematic literature search (PROSPERO registration CRD42016053675) on PubMed and Web-Of-Science, using terms that reflect diagnoses and structures of interest, as well as lesional mechanisms. Two researchers reviewed the articles separately according to PRISMA Guidelines, selecting reports of adult-onset hypomania, mania or mixed state following a focal brain lesion, for pooled-analyses of individual patient data. Eligible lesion images were manually traced onto the corresponding MNI space slices, and lesion topography analyzed using standard brain atlases. Using this approach, data from 211 lesional mania patients was extracted from 114 reports. Among 201 cases with focal lesions, more patients had lesions involving exclusively the right (60.7%) than exclusively the left (11.4%) hemisphere. In further analyses of 56 eligible lesion images, while findings should be considered cautiously given the potential for selection bias of published lesion images, right-sided predominance of lesions was confirmed across multiple brain regions, including the temporal lobe, fusiform gyrus and thalamus. These, and several frontal lobe areas, were also identified as preferential lesion sites in comparisons with control lesions. Such pooled-analyses, based on the most comprehensive dataset of lesional mania available to date, confirm a preferential association with right-hemisphere lesions, while suggesting that several brain areas/circuits, relevant to mood regulation, are most frequently affected.

## Introduction

Bipolar disorder (BPD) and other bipolar spectrum conditions, affecting 3–6% of the population worldwide^1^, manifests as a recurrent, episodic disturbance of mood, sleep, behavior, and perception, including at least one episode of acute mania or mixed affective state. While the first manifestations of BPD are often depressive episodes, diagnosis is typically established after the first manic, hypomanic or mixed episode. The overwhelming majority of such episodes are idiopathic, leading to a diagnosis of primary BPD. Secondary manic, hypomanic or mixed affective states, in contrast, refers to cases where a manic, hypomanic or mixed episode first appears after an organic insult, including structural brain lesions. Common causes include stroke, traumatic brain injury, or tumors^2^. Distinguishing lesional mania from primary bipolar disorder may be challenging, as there is no clear difference in presenting symptoms^3^. Typically occurring at a later age (39 ± 15^2^ vs 20.2 ± 11.8 years^4^), the diagnosis of lesional mania, hypomania or mixed affective state requires the occurrence of an identifiable brain insult before the onset of the inaugural manic episode. While temporal proximity between the onset of manic symptoms and ocurrence of the brain insult supports the diagnosis, the typical temporal interval has not been clearly defined, and may vary from days to years^3^. Satzer and colleagues stress that a high index of suspicion for lesional mania is necessary, and that clinicians should consider this diagnosis in cases with the following characteristics: focal or soft neurological signs; atypical manic features (visual or olfactory hallucinations, clouding of consciousness, disorientation, or memory impairment); initial presentation at an older age (≥40 years); uncommon illness course (single manic episode, unremitting or refractory mania)^3^.

Although lesional mania, hypomania or mixed affective state has traditionally been associated with right-hemisphere brain lesions^5^, the evidence supporting this claim is mostly anecdotal^6^. Moreover, while a recent narrative review found that thalamus, hypothalamus, basal ganglia and frontal and temporal cortices were the most frequent lesion locations^3^, it remains unresolved if lesional mania, hypomania or mixed affective state predominantly involves a specific brain area or network. Nevertheless, studying lesional mania, hypomania or mixed affective state may be a valuable approach to understand the neuroanatomy of primary mania and BPD. In fact, the direction of causal associations between brain structure and behavioral changes is clearer for lesional mania, hypomania or mixed affective state than for primary BPD. Furthermore, this approach may highlight brain areas and networks missed by comparative image protocols, the latter being inevitably contaminated by unspecific, non-causal positive findings^7^.

Here we present the results of a systematic literature review on lesional mania, hypomania or mixed affective state, with pooled analyses of anatomical data reported for individual cases, as well as comparisons of these data with those of lesion distribution in control populations. While the main goal of this pooled analysis was to confirm whether brain lesions in lesional mania, hypomania or mixed affective state are more often right- than left-sided, we further explored whether lesions converged on specific areas or circuits relevant to mood regulation.

## Materials and methods

### Protocol and registration

The protocol was published in PROSPERO database (CRD42016053675) and can be consulted for full description of methods (http://www.crd.york.ac.uk/PROSPERO/display_record.asp?ID=CRD42016053675).

### Information sources and search strategy

Search was performed on PubMed and Web-of-Science between May 2015 and April 2019. Search terms reflected diagnoses of interest (bipolar disorder, manic, mania), structures of interest (cerebral, cerebellum, brain, central nervous system) and possible mechanisms of lesion (injury, tumor, neoplasm, mass, infection, abscess, cyst, stroke, hemorrhage, bleeding). Filters were applied to restrict search results to adult human subjects (Supplementary Table S1). No restrictions were applied to publication year.

### Study selection and eligibility criteria

After eliminating duplicates, two researchers reviewed the list of articles separately, selecting eligible reports according to PRISMA procedures. Articles in English, French, German, Portuguese or Spanish were considered, regardless of publication date or country of origin. Eligible cases were 18 years or older, with a distinct episode of behavioral change lasting at least 4 days, and causing significant psychosocial impairment, manifesting with elevated, expansive, or irritable mood and abnormally and persistently increased goal-directed activity or energy, as well as at least three of the following: inflated self-esteem or grandiosity, decreased need for sleep, excessive talkativeness, flight of ideas, distractibility, increased goal-directed activity, and excessive involvement in activities with potentially painful consequences^8^. Reports that did not provide details on behavioral changes remained eligible if authors explicitly stated that they met contemporary DSM or ICD criteria for manic, hypomanic or mixed affective state. Eligibility further required at least one confirmed brain lesion that preceded the first manic/hypomanic manifestations. Cases where a brain lesion was diagnosed after the first manic/hypomanic manifestations were considered if the lesion was unequivocally acquired prior to onset of the manic, hypomanic or mixed affective state. Cases were excluded if no brain lesion was identified, the chronology between lesion occurrence and manic symptoms could not be unequivocally established, or if the brain lesion occurred after the first manic syndrome. Literature reviews or meta-analyses were excluded, but were screened for additional references, as were reference lists of eligible articles.

### Data extraction, data items and risk of bias

Two researchers extracted data separately according to PRISMA guidelines. For each paper, author name, title and journal, publication year, study type, and number of reported and eligible cases was recorded. For each eligible case, we noted age at first episode of lesional mania, gender, hand dominance, time-interval between brain lesion and mania onset, availability of lesion image (MRI, CT, SPECT, drawing on a standard brain atlas, or photographs of autopsy specimens), lesion location and nature as described by original authors, previous history of depression, personal or family history of other neuropsychiatric disorders, and mania symptoms mentioned in the case description. Additional clinical information was extracted, namely medication at the onset of mania, medication used to treat the manic episode, duration of manic symptoms, length of follow-up and subsequent affective episodes. Extraction of personal and/or family history of neuropsychiatric disorders was fully dependent on this information being explicitly reported in each case report and included, among others, anxiety disorders, substance use disorders, Parkinson’s disease, epilepsy and multiple sclerosis. For systematic assessment of study quality we created a Clinical Quality Assessment scale (CQA) and a Brain Lesion Documentation Assessment scale (BLDA) since, after thoroughly searching for quality assessment tools that might be used to assess the quality of case reports and case series, we found none that could be usefully employed for the purposes of this literature search (please see Supplementary Material and Table S2 for full details). Eligible lesion images (i.e. BLDA ≥ 3) were manually transcribed onto the corresponding slices of the MNI_ICBM152NLin2009 template (http://www.bic.mni.mcgill.ca/ServicesAtlases/ICBM152NLin2009), using MITK software v2014.10.00 (http://mitk.org/wiki/MITK). We only traced tissue damage that was clearly visible in the available images and did not extrapolate to juxtaposed brain slices. Two of the authors, including a neuroradiologist, performed this task jointly, and a third author, who is a neurosurgeon, independently reviewed lesion traces. When tracing, each case report matched a single individual, except for 5 case series^9–12^. In these case series, representing a total of 54 patients (*n* = 7; *n* = 7; *n* = 11; *n* = 12; *n* = 17) individual lesions were fused into a single conjoint lesion tracing, from which the original individual lesions were impossible to disentangle. For these series, we considered each group tracing as a single case, hence comprising a total of 5, rather than 54, cases.

### Summary measures and synthesis of the results

Based on descriptions by the original authors and/or available brain images, we classified each lesion according to laterality and affected brain region(s) into whole-brain regions of interest (ROIs) defined a priori by three of the authors (psychiatrist, neuroradiologist and neurosurgeon). For traced lesion images, quantification of lesion distribution in grey-matter (GM) and white-matter (WM) into more specific ROIs was performed on Anatomist software (http://brainvisa.info/web/download.html), using the Automated Anatomical Labeling atlas (AAL; http://www.gin.cnrs.fr/en/tools/aal/), and the John Hopkins University (JHU) WM tractography atlas (http://cmrm.med.jhmi.edu/), respectively.

### Statistical analysis

Data are presented as % patients or mean ± standard deviation (SD). To test the null hypothesis that lesions were randomly distributed across both brain hemispheres, and because the same subject may have both right- and left-hemisphere lesions, we used McNemar’s test for repeated measures, to compare the proportion of patients with left- vs. right-sided lesions in the entire sample with focal lesions, first for the whole brain and then for each pre-defined ROI. Because the likelihood of suffering right-hemisphere and left-hemisphere lesions is not independent, a within-subjects test such as the McNemar’s is necessary^13^. In traced lesion images we further compared, for each area of the AAL and JHU atlases, the proportion of affected voxels on the left- vs. right-hemisphere. Because data did not follow a normal distribution, a non-parametric test was necessary. Since visual inspection of the distribution plots of the left–right differences in lesioned voxels showed that the distribution of these differences around 0 was not always symmetrical, we opted for the more conservative Sign Test, where the symmetry assumption is not required^13^.

Furthermore, we compared lesion distribution among our tumor sub-sample with data for 169,934 adult-onset brain tumours reported in a database published by Ostrom et al., not selected for any particular clinical outcome^14^. We considered this sample as the best approximation to the normative anatomical distribution of tumors in the general population. To perform this comparison, we classified tumors in our database according to the criteria used by Ostrom et al., and compared the proportions of patients with lesions in each brain region with those reported by Ostrom et al., using Fisher’s exact tests due to small expected counts (<5) in 2 × 2 contingency tables^13^.

For cases of right-sided vascular lesions with traced lesion images, we compared the proportion of affected voxels in each area of the GM and WM atlases to that in a sample of 439 right-hemisphere stroke patients, not selected for any particular outcome^15^, and for whom we had access to three-dimensional (3D) MRI images, allowing for more detailed comparative analyses. This sample was considered as an approximation to the normative anatomical distribution of right-hemisphere strokes in the general population. Since we had access to a maximum of 5 scan slices from lesional mania patients, as opposed to full 3D brain scans from the right-hemisphere stroke group, rendering a direct comparison uninterpretable, we decreased the number of slices included per patient in the latter group. Specifically, for each scan in the right-hemisphere stroke group, we found the axial slice with the maximum extent of damage, and considered this slice, as well as up to four neighboring lesion-englobing slices, at 8 mm intervals above and/or below the main slice^15^. The proportion of scans from the right-hemisphere stroke group contributing with 1, 2, 3, 4 or 5 slices was determined according to the equivalent proportions in the lesional mania case-reports, so as to render the groups comparable, and with the number of slices extracted from each subject reflecting lesion extent (please see *Matlab Script* supplementary file for full details). Because the proportion of lesioned voxels per area per individual did not follow a normal distribution, we compared the two groups using the Wilcoxon rank-sum test, a non-parametric test for two independent samples^13^.

For all analyses, statistical significance was defined according to Benjamini-Hochberg^16^, assuming a false discovery rate (FDR) of 0.1. All statistical analyses were performed in StataCorp. 2017. Stata Statistical Software: Release 15. College Station, TX: StataCorp LLC.

## Results

### Literature review

**Fig. 1:**
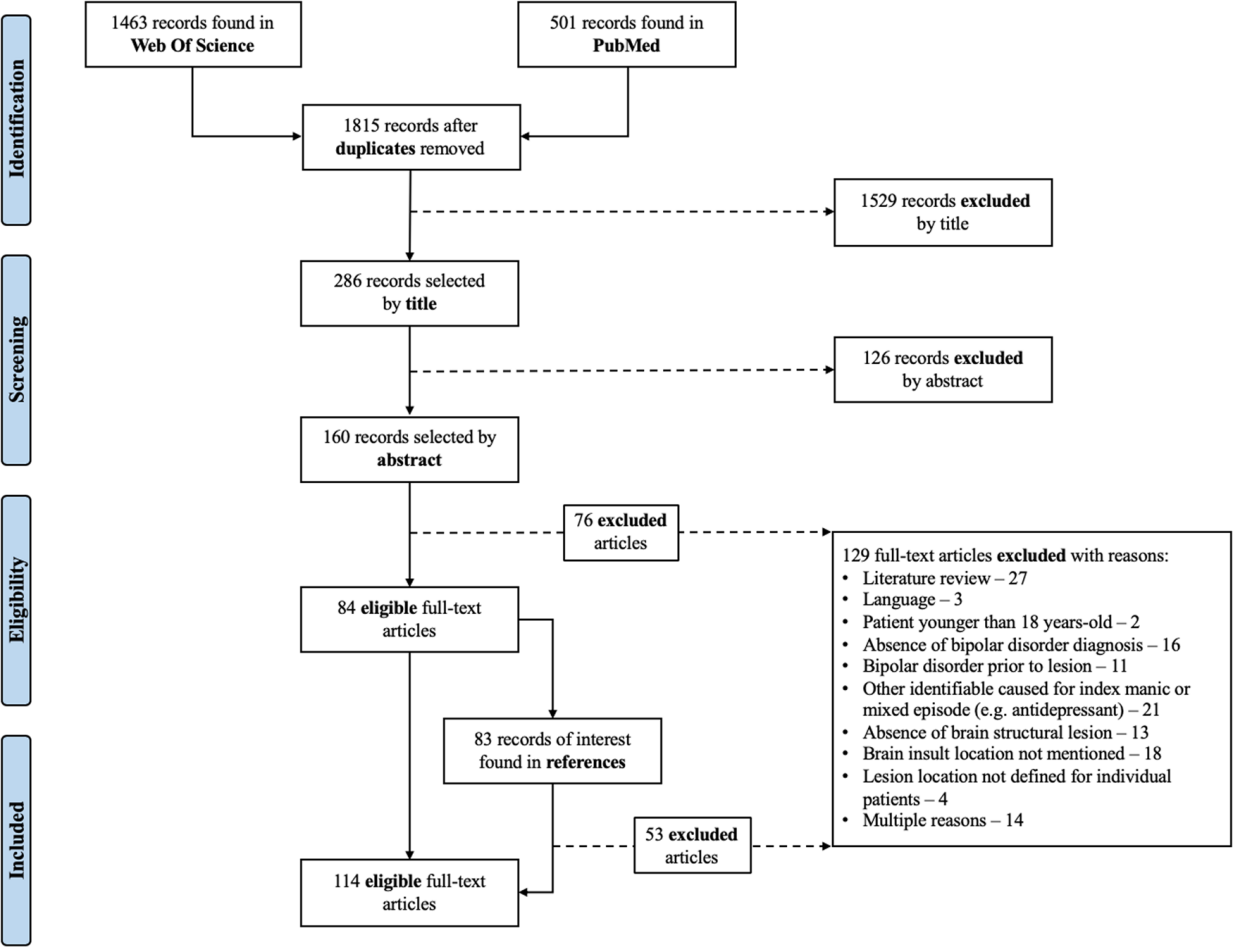
Article selection flowchart. Article selection ws performed according to PRISMA Statement.

Literature review identified 114^9–12,17–126^ eligible articles (Fig. 1 and Supplementary Table S3) published from 1928 to 2018, comprising 211 case-descriptions, including both focal lesions, involving at least one circumscribed brain area^127^, and diffuse lesions, where damage was spread over wide or multiple brain areas. Brain lesion documentation was provided for 118 patients (55.9%), namely from MRI (*n* = 32), CT (*n* = 29), schemes/drawings (*n* = 55), and autopsy photographs (*n* = 2). Fifty-one of these cases were traced on the MNI atlas. Thirteen cases were not eligible due to low image quality (BLDA ≤ 2) or because lesions were diffuse. Lesion tracings obtained from 5 case series^9–12^, that depicted several individual lesions jointly in group tracings, and comprising a total of 54 patients (*n* = 7; *n* = 7; *n* = 11; *n* = 12; *n* = 17), were processed as 5 individual cases for the purpose of lesion topography analysis. Thus, there was a total of 56 lesion tracings for analysis.

### Results and synthesis of studies

Mean age at lesional mania onset was later than what is typically reported for primary BPD (48.6 ± 17.5 vs. 20.2 ± 11.8 years in Morken et al.^4^), and most patients were male (63.7%) and right-handed (87.6%). A prior history of depression was mentioned in 14.2% of patients, and 27.6% had another neuropsychiatric diagnosis, epilepsy being most common. Just over half of the cases developed manic symptoms in the first month after the respective brain lesion. Mood elation and psychomotor agitation were the most frequently reported symptoms (90%) and psychotic symptoms were present in almost 50% of cases (Supplementary Table S4). At the onset of the manic syndrome 17.7% of patients were reported to be taking any type of medication, with antihypertensives being the most commonly reported (8.1% of all patients) and 8.9% of patients taking antiepileptics, antipsychotics, benzodiazepines and/or other central nervous system agents. On average, manic symptoms persisted for 4 months, with follow-up period after the onset of the index mania episode varying between 2 weeks and 28 years, and subsequent affective episodes reported in half of the cases. We found no differences in terms of episode recurrence or length of follow-up between patients who developed mania less than 1 month post-lesionally and patients who developed mania at a later stage (>1 M). Most cases were secondary to vascular lesions (51.3%) or tumors (20.4%). Comparison between patients with vascular and non-vascular lesions revealed that the former were older, more frequently right-handed and had a less frequent history of depression (Table 1).

**Table 1:** Demographic and clinical data extracted from eligible cases.

Characteristic	Total sample (*n* = 211)	Vascular vs. non-vascular etiology^a^		
		Vascular (*n* = 77)	Non-Vascular (*n* = 70)	*p* value
	Mean ± SD (*N*) or *N* (%)	Mean ± SD (*N*) or *N* (%)	Mean ± SD (*N*) or *N* (%)	
Age at onset (Years)	48.5 ± 17.4 (195)	56.0 ± 16.4 (57)	43.2 ± 15.0 (69)	<0.0001^b^
Gender				
Female	74 (36.1)	23 (32.4)	25 (35.7)	n.s.^c^
Male	131 (63.9)	48 (67.7)	45 (64.3)	
Hand Dominance				
Right	93 (87.7)	33 (97.1)	9 (69.2)	0.01^c^
Left/Amb.	13 (12.3)	1 (2.9)	4 (30.8)	
Time E-MM				
≤1 m	38 (52.8)	25 (62.5)	13 (40.6)	n.s.^c^
>1 m	34 (47.2)	15 (37.5)	19 (59.4)	
Aetiology				
Vascular	101 (51.8)			
Tumour	40 (20.5)			
TBI	24 (12.3)			
Other	30 (15.4)			
Previous Depression				
Yes	25 (15.2)	8 (12.3)	11 (20.4)	
No	140 (84.8)	57 (87.7)	43 (79.6)	n.s.^c^
Personal history of other NP disorder				
Yes	48 (28.7)	10 (16.4)	22 (36.7)	
No	119 (71.3)	51 (83.6)	38 (63.3)	0.01^c^
MM Duration (Months)	4.4 ± 14.2 (59)	2.2 ± 2.6 (28)	6.3 ± 19.4 (31)	n.s.^b^
Follow-up Time (Months)	32.1 ± 53.1 (68)	28.1 ± 61.1 (32)	35.6 ± 45.5 (36)	n.s.^b^
Affective episode recurrence				
Yes	42 (50.6)	20 (48.8)	22 (52.4)	
No	41 (49.49)	21 (51.2)	20 (47.6)	n.s.^c^

*Amb* ambidextrous, *E* event causing brain insult, *E-MM* time in months (m) between event causing brain insult and the manic/mixed state episode onset, *NP* neuropsychiatric.

^a^Does not include the following case series, which did not provide enough information on individual lesion etiology: Carran 2003, Robinson 1988, Starkstein 1987 and Starkstein 1991 (See Supplementary Material for complete references – Supplementary Table S3).

^b^*p* value for two-sample *t* test comparing vascular vs. non-vascular etiology.

^c^*p* value for Fisher’s exact tests comparing vascular vs. non-vascular etiology.

### Anatomical distribution of lesions

**Fig. 2:**
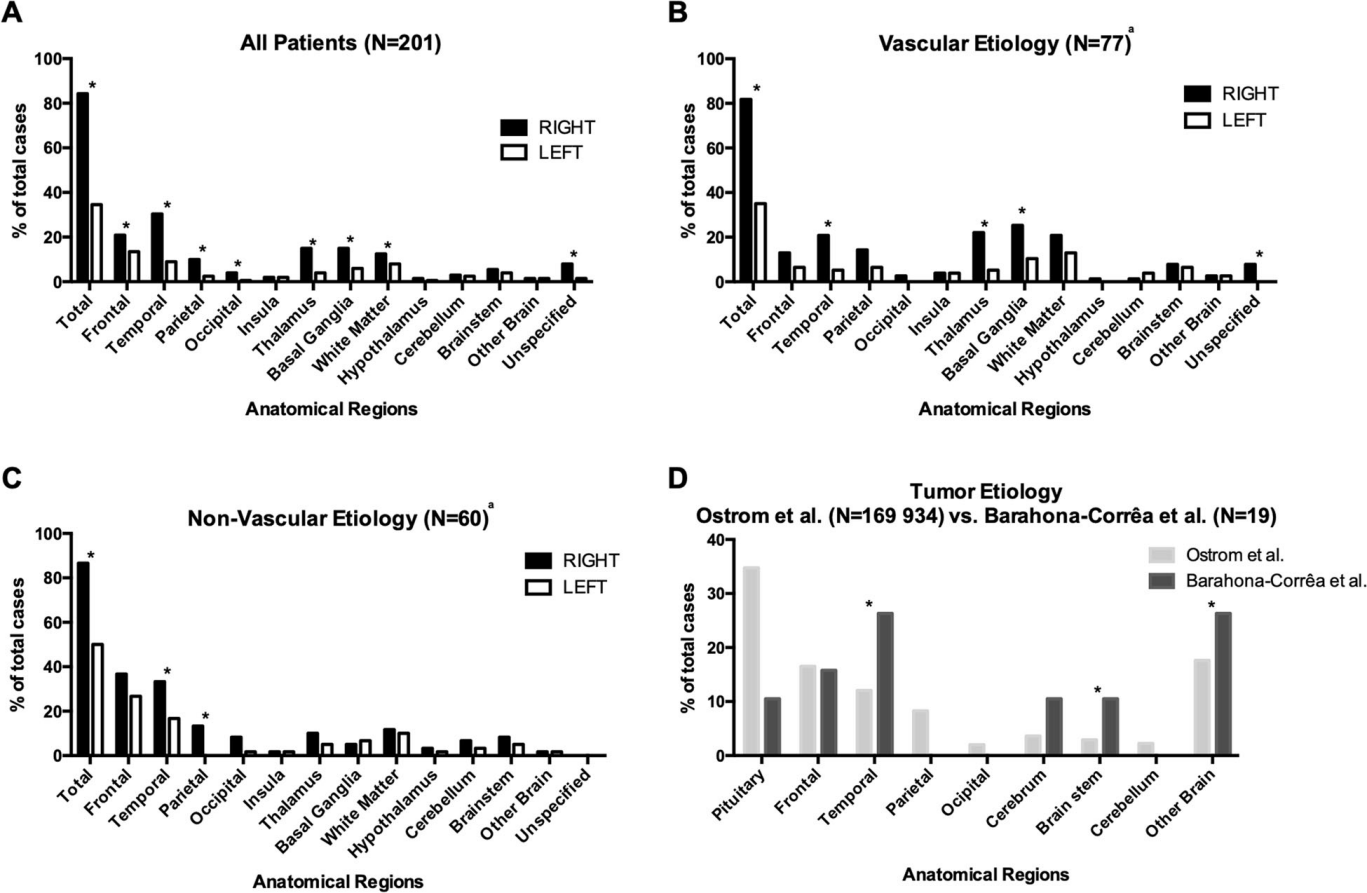
Lesion distribution by major brain areas. **a** Lesion distribution for all cases in the literature review. **b** Lesion distribution for cases with vascular lesions. **c** Lesion distribution for cases with non-vascular lesions. **d** Comparison of lesion distribution for tumor cases identified in this literature review (*n* = 19) with tumor distribution described by Ostrom et al. for a large patient database (*n* = 169934)^14^. In what brain tumors are concerned, regions were defined according to the International Classification of Diseases for Oncology (ICD-O), without considering tumours originating from the meninges (*n* = 14), ventricles (*n* = 2), cranial nerves (*n* = 1), or of unspecified origin (*n* = 4). “Other Brain” refers to lesions spanning multiple areas (C71.8: “neoplasm involving two or more sites, corpus callosum and tapetum”) or when areas were insufficiently specified (C71.9: “intracranial site, cranial fossa not otherwise specified, anterior cranial fossa, middle cranial fossa, posterior cranial fossa and suprasellar”). “Cerebrum” refers to multiple subcortical structures (C71.0: “basal ganglia, central white matter, unspecified cerebral cortex, cerebral hemisphere, cerebral white matter, corpus striatum, globus pallidus, hypothalamus, insula, internal capsule, island of Reil, operculum, pallium, putamen, rhinencephalon, supratentorial brain not otherwise specified and thalamus”). ^a^Does not include the following case series, which did not provide enough information on individual lesion etiology: Carran 2003, Robinson 1988, Starkstein 1987 and Starkstein 1991 (See Supplementary Material for complete references – Supplementary Table S3). *p value < 0.05.

**Fig. 3:**
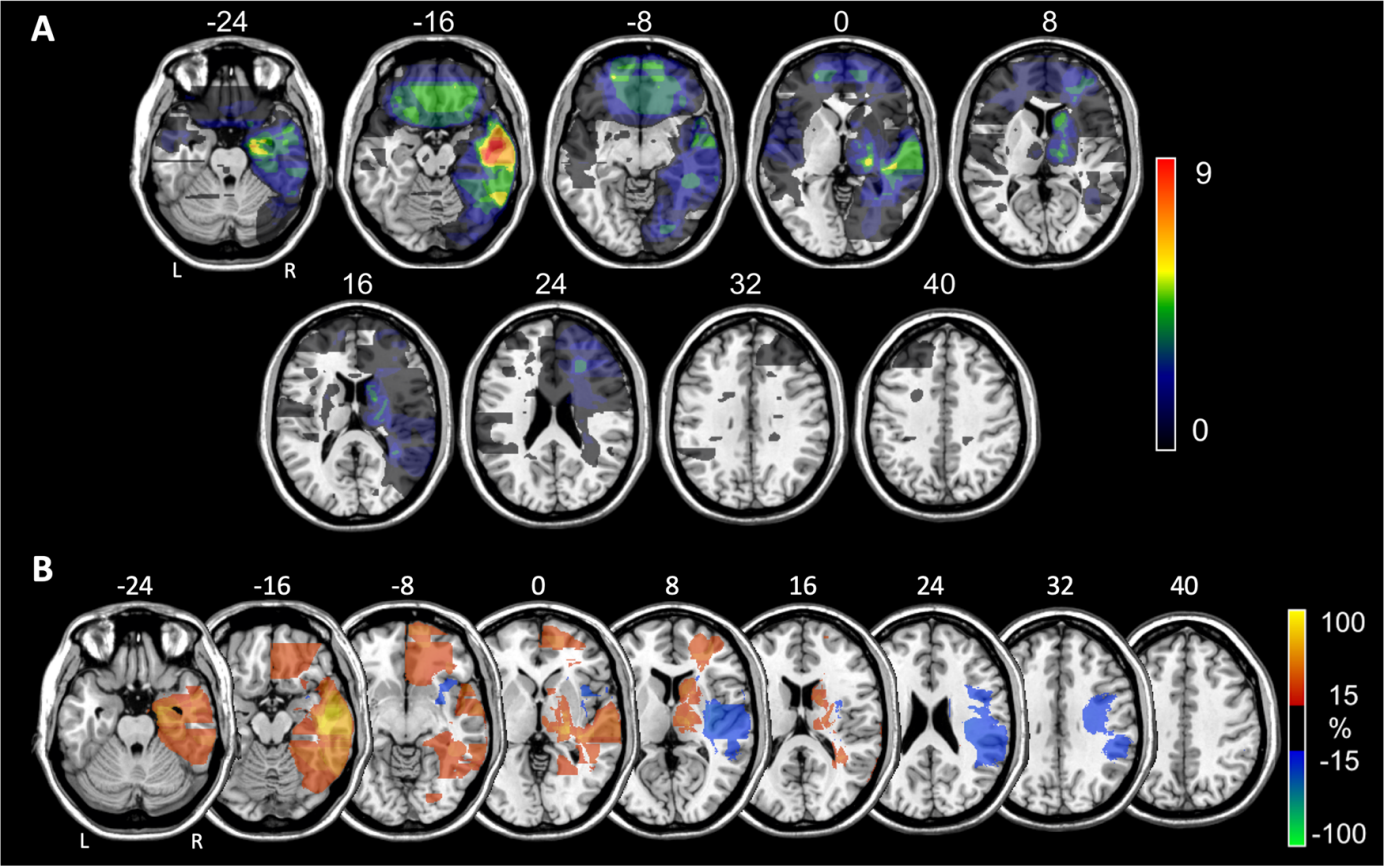
Distribution of brain lesions associated with secondary mania in 56 patients with eligible lesion images. **a** Comparison between right vs. left-sided lesions. Each lesion was traced manually onto a common brain atlas (MNI) and projected on the closest depicted slice. Numbers above slices indicate z-coordinates in MNI space. The color code indicates maximal number of lesions overlapping on a single voxel. **b** Subtraction plot contrasting 29 right-sided stroke patients with secondary bipolar disorder (red-yellow) versus 439 unselected right hemisphere stroke patients (blue-green). In this plot, a value of, for example, 30, reflects that the voxel is damaged 30% more frequently in patients with secondary bipolar disorder than in unselected patients (for more details on the method see Rorden and Karnath^7^). To improve visualization, lesions of mania patients were projected onto the closest depicted slice before plot generation.

Among 201 cases with focal brain lesions, these were exclusively right-sided in 60.7%, exclusively left-sided in 11.4%, bilateral in 21.9% and midline in 4.0% (*p* < 0.0001, McNemar’s test). Thus, 169 patients (84.1%) had right-hemisphere lesions, while only 71 (35.3%) had left-sided lesions. Further comparisons demonstrated a significantly higher proportion of lesions on the right, relative to left hemisphere, in the frontal (*p* = 0.005), temporal (*p* = 0.00001), parietal (*p* = 0.002), and occipital lobes (*p* = 0.008), as well as thalamus (*p* = 0.00001), basal ganglia (*p* = 0.0007) and subcortical WM (*p* = 0.01; McNemar’s tests; FDR corrected; Fig. 2a). Separate comparisons for vascular and non-vascular lesions confirmed an overall predominance of right-sided lesions, in both cases, and for the temporal cortex, basal ganglia and thalamus for vascular lesions only (Fig. 2b, c). Similarly, lesion distribution remained unchanged when we restricted analyses to cases that met strict DSM 5 criteria for mania, and after excluding cases with brain insults caused by neurosurgical interventions or cases that were taking medication of any kind at the time of mania onset (Supplementary Table S4 and Supplementary Fig. S2). Gender, age, hand-dominance, time between brain insult and mania episode, affective episode recurrence and clinical report quality also had no effect on lesion distribution, with only minor differences according to lesion aetiology (Supplementary Table S5 and S6). In quantitative GM and WM analyses of traced lesions (Fig. 3a), when compared to the corresponding areas in the left hemisphere, we found a significantly higher median proportion of lesioned voxels for the right hippocampus, parahippocampal, superior temporal, middle temporal, inferior temporal, lingual and fusiform gyri, caudate, putamen, thalamus and posterior limb of internal capsule (Table 2 and Supplementary Tables S7 and S8).

**Table 2:** Comparison of right hemisphere lesions with left hemisphere lesions and with a control sample of stroke lesions.

Area	% lesioned voxels^a^ (*n* = 56)					% lesioned voxels^a^				
	Left		Right		*p* (Sign test)^b^	Sperber et al. (*n* = 439)		Barahona-Corrêa et al. (*n* = 29)		*p* (Rank-sum test)^c^
	Mean	SD	Mean	SD		Mean	SD	Mean	SD	
Grey matter										
Frontal										
Precentral gyrus	0.01	0.04	0.01	0.06	n.s.	0.23	0.7	0.01	0.03	0.009
Superior frontal gyrus	0.09	0.4	0.1	0.48	n.s.	0.05	0.3	0.05	0.16	n.s.
Superior frontal gyrus, orbital part	0.69	2.08	0.66	2.11	n.s.	0.03	0.25	0.56	2.00	0.005
Middle frontal gyrus	0.08	0.41	0.14	0.57	n.s.	0.17	0.63	0.09	0.35	n.s.
Middle frontal gyrus, orbital part	0.49	1.73	0.62	2.09	n.s.	0.07	0.4	0.58	2.20	n.s.
Inferior frontal gyrus, pars opercularis	0.04	0.24	0.1	0.56	n.s.	1.04	2.24	0.13	0.72	0.0007
Inferior frontal gyrus, pars triangularis	0.15	0.70	0.15	0.82	n.s.	0.73	1.97	0.20	1.09	0.004
Inferior frontal gyrus, pars orbitalis	0.5	1.78	0.51	1.78	n.s.	0.17	0.83	0.38	1.36	n.s.
Rolandic operculum	0.14	0.78	0.14	0.83	n.s.	1.72	3.40	0.24	1.15	0.001
Supplementary motor area	0	0	0.01	0.07	n.s.	0.04	0.46	0	0	n.s.
Olfactory cortex	0.43	1.81	0.5	2.08	n.s.	0.03	0.27	0.26	1.07	n.s.
Medial frontal gyrus	0.11	0.46	0.13	0.58	n.s.	0.04	0.39	0.08	0.39	n.s.
Medial orbitofrontal cortex	0.89	3.14	0.93	3.18	n.s.	0.04	0.42	0.95	3.13	0.0006
Rectus gyrus	0.75	2.81	0.8	2.95	n.s.	0	0.07	0.55	2.06	0.005
Temporal and insula										
Hippocampus	0.01	0.06	0.45	1.47	2.0 × 10^−5^	0.21	0.78	0.57	1.44	9.3 × 10^−7^
Parahippocampal gyrus	0.03	0.14	0.68	1.88	0.006	0.09	0.39	0.91	2.20	0.004
Amygdala	0.05	0.39	0.27	0.86	n.s.	0.07	0.58	0.43	1.08	1.0 × 10^−6^
Transverse temporal gyrus (Heschl)	0.12	0.91	0.20	1.06	n.s.	1.73	3.51	0.39	1.46	0.01
Superior temporal gyrus	0.20	1.03	0.43	1.16	0.01	1.33	2.57	0.66	1.31	n.s.
Superior temporal pole	0.15	0.73	0.36	0.87	n.s.	0.16	0.61	0.58	1.09	0.0004
Middle temporal gyrus	0.12	0.55	0.53	1.28	0.008	0.75	1.69	0.82	1.42	n.s.
Middle temporal pole	0.22	0.98	0.25	0.73	n.s.	0.02	0.26	0.39	0.94	2.1 × 10^−18^
Inferior temporal gyrus	0.03	0.1	0.54	1.54	0.01	0.13	0.67	0.76	1.80	5.0 × 10^−6^
Insula	0.25	1.06	0.25	0.93	n.s.	1.47	2.59	0.4	1.24	0.02
OCCIPITAL										
Calcarine sulcus	0.01	0.04	0.02	0.11	n.s.	0.23	0.88	0.01	0.05	n.s.
Cuneus	0	0	0	0	NA	0.09	0.5	0	0	n.s.
Lingual gyrus	0.002	0.013	0.32	1.08	0.02	0.37	1.4	0.31	1.1	n.s.
Superior occipital	0.002	0.013	0.003	0.022	n.s.	0.17	0.69	0.01	0.03	n.s.
Middle occipital gyrus	0.004	0.021	0.02	0.16	n.s.	0.4	1.19	0.04	0.22	0.04
Inferior occipital	0.005	0.024	0.41	1.64	n.s.	0.26	1.21	0.15	0.59	n.s.
Fusiform gyrus	0.01	0.08	0.61	1.88	6.0 × 10^−5^	0.1	0.47	0.76	2.16	9.0 × 10^−6^
Parietal										
Postcentral gyrus	0.02	0.16	0.02	0.09	n.s.	0.26	0.72	0.01	0.08	0.01
Superior parietal lobule	0	0	0	0	NA	0.04	0.39	0	0	n.s.
Inferior parietal lobule	0	0	0	0	NA	0.17	0.89	0	0	n.s.
Supramarginal gyrus	0.08	0.58	0	0	n.s.	0.64	1.67	0	0	0.002
Angular gyrus	0.01	0.05	0.002	0.013	n.s.	0.36	1.2	0	0	0.01
Precuneus	0.002	0.01	0.002	0.013	n.s.	0.05	0.28	0	0	n.s.
Paracentral lobule	0	0	0.02	0.12	n.s.	0.03	0.42	0	0	n.s.
Cingulum										
Anterior cingulate gyrus	0.23	0.93	0.26	1.19	n.s.	0.06	0.55	0.09	0.35	n.s.
Midcingulate gyrus	0	0	0.002	0.01	n.s.	0.08	0.56	0	0	n.s.
Posterior cingulate gyrus	0	0	0	0	NA	0.02	0.31	0	0	n.s.
Subcortical grey matter										
Caudate nucleus	0.04	0.17	0.40	1.05	0.0005	0.71	1.68	0.52	1.13	n.s.
Putamen	0.02	0.08	0.27	1.21	n.s.	1.65	3.08	0.52	1.66	n.s.
Globus pallidum	0.06	0.45	0.42	1.53	n.s.	1.15	2.9	0.78	2.07	n.s.
Thalamus	0.05	0.23	0.55	1.61	0.0005	0.35	1.12	0.75	1.59	0.006

For both analyses, statistical significance was defined using a False Discovery Rate (FDR) of 0.1, according to Benjamini-Hochberg16.

*NA* not applicable, *n.s.* non-significant, *SD* standard deviation.

^a^Displayed values are means and standard deviations. Please see Supplementary Table S8 for medians, minimums and maximums.

^b^*p* value for Sign tests comparing the left- and right-hemisphere lesion volumes based on quantitative GM and WM analysis. The values being compared reflect the median proportion of voxels in each AAL and JHU atlas area that are included in the lesion.

^c^*p* value for Wilcoxon rank-sum tests comparing lesion volumes based on quantitative GM and WM analysis between vascular lesional mania cases and an unselected sample of right-sided stroke described by Sperber and Karnath^15^. The values being compared reflect the median proportion of voxels in each AAL and JHU atlas area that are included in the lesion.

To further confirm the relevance of specific brain regions, we then compared lesion distribution among specific sub-samples of our database with that of patients with lesions of similar etiology, but not selected for any particular symptom. Compared to the distributions of brain tumors described by Ostrom et al.^14^, tumors associated with lesional mania were more frequently located in the temporal lobes, brain stem, and cerebrum, which includes the thalamus and basal ganglia (Fig. 2d). Among lesional mania patients with traced right-sided vascular lesions, a more detailed comparison with MRI scans from 439 right-hemisphere stroke patients described by Sperber and Karnath^15^ showed that lesional mania patients had a higher median proportion of lesioned voxels in the orbital part of the superior frontal gyrus, medial orbitofrontal cortex, rectus gyrus, hippocampus and parahippocampal gyrus, amygdala, superior and middle temporal poles, inferior temporal gyrus, fusiform gyrus and thalamus (Table 2 and Supplementary Tables S7 and S8). In contrast, the precentral gyrus, inferior frontal gyrus, rolandic operculum, transverse temporal gyrus, middle occipital gyrus and several parietal regions showed a lower median proportion of lesioned voxels in lesional mania cases. Results were similar when we compared the percentage of patients lesioned in each area (please see Supplementary Table S9 for further details). These results, as well as quantitative GM and WM analyses of traced lesions, did not significantly change after restricting analyses to patients without personal or family history of psychiatric disorder, with non-vascular lesion etiology, that fulfill DSM 5 criteria for manic episode, and with CT or MRI images available for analysis (Supplementary Tables S10 and S11).

## Discussion

The main aim of this work was to test the hypothesis that lesional mania is more often associated with right-hemisphere than left-sided lesions. Towards this aim, we performed a systematic literature review of case-reports and case-series, followed by pooled analysis of individual patient and lesion data from eligible cases. We retrieved 211 cases of lesional mania, occurring at a much older age than the typical age of onset reported for primary mania, probably reflecting the cumulative age-related increase in the likelihood of suffering a brain lesion associated to cardio-vascular diseases and tumors. For most of these cases, right-hemisphere focal brain lesions (84.1%) were reported, with only 35.3% of patients having left-sided lesions. This difference was statistically significant and was conserved across different disease subtypes as well as in analyses restricted to patients without personal or family history of neuropsychiatric disorder.

To our knowledge, this is the first systematic review and pooled analysis of published cases of lesional mania, offering the most comprehensive demonstration of a long-held, albeit empirically unconfirmed, axiom of textbook neuropsychiatry, i.e., that lesional mania is preferentially associated with right-hemisphere, rather than left-hemisphere, brain lesions, as suggested in previous work based on smaller samples^10,113^. Robinson and colleagues found a predominance of right-sided brain lesions in 17 lesional mania cases when compared to 31 patients with post-stroke depression, who had predominantly left-sided lesions^10^. Most lesional mania patients had lesions involving the right orbitofrontal and basotemporal cortex, caudate and thalamus, while patients with post-stroke depression had more widely distributed lesions predominantly involving the head of the left caudate and the left insular and basotemporal cortex^10^. Moreover, there was no overlap between right-sided lesions associated with mania and right-sided lesions associated with depression^10^. Starkstein and colleagues reported similar findings in an independent cohort of eight lesional mania patients and further complemented the anatomical analysis with results from ^18^fluorodeoxyglucose (^18^FDG) positron emission tomography performed in 3 patients, which showed a lower ^18^FDG uptake in several right limbic regions including lateral basotemporal and superior frontal areas^113^.

Additionally, our findings of preferential lesions of the right brain in lesional mania patients is convergent with evidence for lateralized differences in brain anatomy of primary BPD, when compared to healthy controls, as suggested in recent voxel-based meta-analyses of GM and WM volume. In one meta-analysis of eight voxel-based morphometry studies, Selvaraj and colleagues found a right-sided contiguous cluster of GM reduction in BPD patients compared to controls, encompassing the insula, middle and superior temporal gyrus, temporal pole, inferior frontal gyrus (pars opercularis and triangularis) and claustrum^128^. More recently, in two other voxel-based meta-analyses, Wise and colleagues described clusters of reduced GM volume in the right middle occipital, middle temporal and inferior temporal gyri among patients with BPD, as well as decreased fractional anisotropy in the right anterior superior longitudinal fasciculus^129,130^. Consistently with these meta-analytic findings, functional MRI (fMRI) studies in adults with BPD have shown impaired activity or connectivity in multiple right-sided structures^131–133^, with EEG demonstrating a right-left imbalance of frontal alpha-power in hypomanic BPD patients when compared to healthy subjects^134^. This apparent convergence of our results with the literature on primary BPD must, however, be considered with caution, and more systematic follow-up is required to determine the overlap between regions of interest identified in lesional mania studies and among patients with primary BPD.

Our analyses further suggest that right-hemisphere predominance of lesions in lesional mania follows a non-random anatomical distribution, resulting mostly from lesions of specific areas, namely the hippocampus, parahippocampal gyrus, superior, middle and inferior temporal gyri, lingual and fusiform gyri, caudate nucleus, thalamus, and posterior limb of internal capsule (Table 2 and Supplementary Tables S7 and S8). Importantly, we also found significant differences in lesion distribution when comparing lesional mania subsamples with large samples of patients with brain lesions of a similar nature, but not selected for particular behavioral outcomes. Specifically, in comparisons with patients with right-hemisphere stroke^15^, right-sided vascular lesions associated with lesional mania affected all of these areas more frequently, with the exception of the lingual gyrus, superior and middle temporal gyrus, caudate nucleus, and WM areas. This suggests that right-sided predominance of lesions is not merely attributable to a laterality bias in stroke incidence in the general population. Moreover, while the limited quality of lesion topography depiction in most case-reports advises caution in interpreting the over-representation of these areas in lesional mania, it is nevertheless remarkable that these areas have been highlighted by structural or functional neuroimaging studies in primary BPD^135–137^. Two recent case-control studies by the ENIGMA Bipolar Disorder Working Group deserve special mention. Based on large samples comprising thousands of individuals, this consortium found reduced cortical thickness in BPD patients in the middle and inferior temporal gyri, fusiform gyrus, superior frontal gyrus, parahippocampal gyrus, and medial orbitofrontal and anterior cingulate cortices, as well as reduced volumes of the hippocampus and thalamus^138,139^. While the functional and neuropathological underpinnings of these volumetric abnormalities remain unclear, it is nevertheless noteworthy that almost all of these regions and structures emerged from our analysis as being more frequently affected in lesional mania compared to the control group, suggesting that reduced volumes in these areas may reflect loss of function and/or dysconnectivity.

Temporal lesions, in particular those affecting the right hippocampus and parahippocampal gyrus, were, in addition to the thalamus, the most consistently over-represented in lesional mania, both in left–right comparisons and comparisons with tumor and vascular controls. In fact, there is reasonable consensus regarding hippocampal abnormalities in primary BPD, but it remains unclear if they reflect treatment effects or disease progression, rather than neural vulnerability for the disorder^135^, with our results supporting the latter hypothesis. Occipital cortical areas, fusiform and lingual gyri in particular, were also over-represented in right-sided lesions, with the fusiform gyrus also more frequently involved in lesional mania when compared to stroke controls. While occipital areas are seldom mentioned in BPD literature, two independent fMRI studies found fusiform gyrus hypoactivation during emotional face processing in primary BPD^140,141^. Other areas, namely the temporal pole, amygdala and several frontal lobe areas, were identified in comparisons with the control stroke sample, but not the left–right comparison (Table 2 and Supplementary Tables S7 and S8). In metanalyses of GM volumetric changes in primary BPD patients compared to control subjects, the temporal pole is part of the right-sided contiguous cluster of volume reduction identified by Selvaraj and collegues^128^, while significantly reduced volumes were found in medial prefrontal areas by Wise and colleagues^130^. Absent lateralization regarding frontal areas could in fact reflect their critical role, with possibly similar functional effects resulting either from a lesion in a particular hemisphere, or from disturbed frontal interhemispheric connectivity secondary to a lesion in the contralateral hemisphere^142^. Finally, as has been reported in other lesion studies^143^, several areas were less frequently affected in vascular lesional mania compared to the vascular control group^15^, possibly reflecting differing vascularization patterns for these areas and those that are over-represented in lesional mania.

The wide distribution of GM areas highlighted by our analyses argues for a potential circuit-based impact of the several different lesions associated with secondary mania. In fact, some of these areas, namely the superior frontal gyrus, including its orbital part, hippocampus/parahippocampal gyrus, and inferior temporal gyrus, partly overlap with the Default Mode Network (DMN)^144^. Gray-matter volume reduction has been found among primary BPD patients in several components of the DMN, namely the prefrontal cortex^137,139,145^, cingulate cortex^136,146^, temporal gyri^146^ and hippocampus^136,138,139^. Consistently, in primary BPD, functional connectivity studies show reduced coherence and connectivity strength in several DMN nodes^131^, and there is evidence of a left-predominant asymmetry of the DMN^147^, further reinforcing the validity of a preferentially right-sided distribution of brain lesions in lesional mania. The higher frequency of superior frontal gyrus and external capsule lesions further suggests a possible disruption, in many cases, of the frontoparietal control network (FPCN)^148^. Interestingly, connectivity between the DMN and the FPCN has recently been shown to correlate with the ability to attend to internal states, a self-monitoring function that is typically disrupted in mania and may be affected in lesional mania by network effects of lesions in components of either of these two networks^149^. In any case, the wide distribution of areas associated with lesional mania merits further analysis, possibly using novel approaches for network localization of symptoms from focal brain lesions, such as lesion network analsysis^150^. This approach involves mapping the lesions onto an atlas of the normative human connectome in order to identify brain regions or networks that are functionally connected to the lesion locations, and has already been used to analyze other lesional neuropsychiatric syndromes^151^.

Our findings, while novel and informative, should be interpreted considering the limitations of the study design. First and foremost, our analyses were restricted to author descriptions of lesions and/or a limited number of slices from each scan, limiting the accuracy of lesion mapping and the validity of topographical analysis. Moreover, the choice of published scan slices by authors could be potentially biased by expectations regarding lesion location. Comparison of case-reports with and without available lesion scans showed that lesion distribution in the two groups is very similar, except for an over-representation of right-sided basal ganglia, brain stem and white matter involvement in reports that provide lesion scans. This possibly reflects the fact that reports that provide lesion scans tend to be more recent, using higher-definition scans that are more likely to detect lesions in deep-brain locations not identifiable in older, lower definition scans (Supplementary Table S6). Irrespective of these limitations, it is important to note that right-hemisphere predominance was restricted to many of the same areas that were identified in the comparison with the stroke control group, cross-validating these findings and suggesting that, for these structures, a specific interaction does exist between laterality and mood regulation. Importantly, authors and reviewers may also have been biased towards publication of cases confirming the conventional view that lesional mania is associated with right hemisphere lesions. Nevertheless, the contrary could also be true, considering the tendency to publish rare associations. In any event, among published reports of lesional mania, the number of right-sided lesions has been consistently higher than left-sided lesions since before the 1970s, arguing against the existence of such a bias (see Supplementary Fig. S1 for details). Several other factors, namely over-diagnosis of milder hypomanic syndromes rather than complete manic syndromes, joint analysis of cases with various etiologies, or effects of medication on the risk of developing mania, may also limit the validity of our findings. However, lesion distribution remained essentially unchanged when we restricted the analysis to cases that met strict DSM 5 criteria for mania, and likewise when we controlled for lesion etiology or medication status at mania onset (Supplementary Table S4 and Supplementary Fig. S2).

Another possible bias could result from under-diagnosis of lesional mania in aphasic patients with left-hemisphere lesions. However, there are several reasons why we believe such a bias does not explain our results. First and foremost, if there was indeed a bias towards underreporting of lesional mania in aphasic patients, we would expect all the cortical areas that are predictive of aphasia when lesioned on the left hemisphere to emerge as being more frequently lesioned on the right-hemisphere in lesional mania patients. However, lesions were not significantly more frequent on the right than on the left hemisphere for any of the cortical areas that are most predictive of Broca’s aphasia or global aphasia (the two most conspicuous forms of aphasia), namely the Inferior Frontal Gyrus, the Pars Triangularis, the Rolandic Operculum, or the Middle Frontal Gyrus (see Yourganov et al.^152^). In fact, for most of these areas, lesions were equally distributed between the left and right hemispheres, while a right-hemisphere predominance did emerge in areas unrelated to language such as the lingual gyrus, the parahippocampal gyrus or the fusiform gyrus (Table 2 and Supplementary Tables S7 and S8). Furthermore, systematic exclusion of aphasic patients would be likely in trials or case series using structured interviews and scales. However, our sample is overwhelmingly composed of sporadic, individual case-reports, where the authors typically offer rich clinical descriptions of psychopathology rather than scale ratings. Unlike depression, mania and hypomania are typically marked by externalized behavior and symptoms that are promptly identifiable even in patients who will not or cannot communicate verbally – a fact well illustrated by the presence of six aphasic patients among our sample of lesional mania patients. Moreover, many patients were assessed several months after the acute lesion, at a time when, in many instances, partial recovery from aphasia is likely to have occurred. Finally, if the greater prevalence of right hemisphere lesions in lesional mania patients was due to a systematic underdiagnosis of lesional mania in aphasic, left-hemisphere lesioned patients, we would expect to find a similar bias towards right-hemisphere predominance of lesions in lesion studies that focused on other behavioral outcomes unrelated to language. Yet, in several neuropsychiatric lesion studies lesions were left-skewed or equally distributed across both hemispheres^151,153,154^, demonstrating that a systematic exclusion of aphasic patients either did not occur or did not lead to such a lateralization bias.

Finally, it is possible that a small number of patients included in the tumor or vascular lesion control populations may have developed manic symptoms, as none of the two databases collected data regarding behavioral manifestations, including systematic neuropsychiatric assessment. However, the rarity of post-lesional mania and the large size of the two control populations mean that any false-negative controls will have a diluted effect. Furthermore, rather than biasing our results in favor of a spurious association, the occurrence of a small minority of manic episodes among the two control populations will, if anything, result in an under-estimation of the true effect size of the associations reported here. While, for these reasons, human lesion studies in Neuropsychiatry are intrinsically limited, they nevertheless provide quasi-experimental insights on the structural neuroanatomy of behavioral disorders, that cannot be achieved by methods such as focal neuromodulation or correlations between symptoms and structural or functional variations observed in non-lesioned brains. As proposed recently by Vaidya et al.^155^, the inferential strength of lesion studies depends on inclusion of various lesion etiologies in the index group and comparisons with an adequately defined control group, sufficiently large to ensure adequate statistical power, as was achieved here.

In conclusion, our study provides the most solid demonstration to date of the association between right-hemisphere brain lesions and the development of secondary mania, while suggesting the first systematic mapping of lesion topography in lesional mania. In that respect, we found that in lesional mania, specific brain areas, distributed across multiple brain regions and circuits, are most frequently affected. We expect these findings will contribute to a more thorough understanding of the role of these brain areas in mood regulation and their importance in the context of bipolar disorders, specifically with regards to lateralization in the control of such functions and in the development of these disorders.

## Supplementary information

Supplemental Material

Matlab Script
