# Supplementary material for: Right-sided brain lesions predominate among patients with lesional mania: evidence from a systematic review and pooled lesion analysis
Source: Transl Psychiatry. 2020 May 12;10:139. doi: 10.1038/s41398-020-0811-0 (PMC7217919; doi:10.1038/s41398-020-0811-0)
Supplement: Supplementary file 1 — Supplemental Material [file 41398_2020_811_MOESM1_ESM.docx]

**Supplementary Material**

**Table S1 – Search syntax**

|  | **Psychiatric Disorder** | **Structure** | **Lesion** | **Not Topic** |
| --- | --- | --- | --- | --- |
| **Pubmed*** | Bipolar Disorder | Cerebral | Injury |  |
|  | Manic | Cerebellum | Tumor |  |
|  | Mania | Brain | Neoplasm |  |
|  |  | Central Nervous System | Mass |  |
|  |  |  | Infection |  |
|  |  |  | Abscess |  |
|  |  |  | Cyst |  |
|  |  |  | Stroke |  |
|  |  |  | Hemorrhage |  |
|  |  |  | Bleeding |  |
| **Web of Science**** | Bipolar Disorder | Cerebral | Injury | Animal |
|  | Manic | Cerebellum | Tumor | Monkey |
|  | Mania | Brain | Neoplasm | Chimpanzee |
|  |  | Central Nervous System | Mass | Mouse |
|  |  |  | Infection | Mice |
|  |  |  | Abscess | Rat |
|  |  |  | Cyst | Cat |
|  |  |  | Stroke | Dog |
|  |  |  | Hemorrhage | Rabbit |
|  |  |  | Bleeding | Bird |
|  |  |  |  | Fish |
|  |  |  |  | Child |

*We applied the following filters: Humans; age +19; English Portuguese, Spanish, German and French

** We applied the following filters: English, Portuguese, Spanish, German and French

**Table S2 – Quality assessment tools for the quality of each case included in the review: Clinical Quality Assessment (CQA) and Brain Lesion Documentation Assessment (BLDA)**

| **Clinical Quality Assessment (CQA)** | **score** |
| --- | --- |
| None of the following clinical elements are described:   - gender; - age at onset of first manic or mixed affective episode; - time-interval between the occurrence of the brain lesion and onset of first manic or mixed affective episode; - personal history of neuropsychiatric disorders; - family history of neuropsychiatric disorders | 0 |
| One, 2, 3, 4 or all 5 of the above clinical elements are described | 1, 2, 3, 4 or 5 |
|  | |
| **Brain Lesion Documentation Assessment (BLDA) *** | **score** |
| Only brain lesion topography description | 1 |
| Brain lesion image is present but not perceptible | 2 |
| Low quality CT Scan, drawing or autopsy photograph | 3 |
| Low quality MRI or high quality CT Scan, drawing or autopsy photograph | 4 |
| High quality MRI image | 5 |

Quality assessment tools to assess case reports and case series according to the purposes of our literature search were not available when planning this project. Existing tools have been originally designed to assess case reports/series describing new interventions, their side-effects and/or their effect on prognosis (1-4), rather than descriptive clinical narratives containing descriptions of brain lesions and associated neuropsychiatric syndromes. As described in our PROSPERO protocol, we thus decided to create novel quality assessment tools. Clinical Quality Assessment (CQA) covers quality of individual case descriptions with regards to details such as gender, time between event and manic syndrome, personal history of psychiatric disorder and family history of neuropsychiatric history (please see Tables S7, Table S8 and S9). Brain Lesion Documentation Assessment (BLDA) addresses quality of the brain lesion description in each report (please see Tables 2, S5, S6 and S7). We conducted several analyses to assess quality of these instruments. We were not able to assess construct validity for CQA, because there is no gold standard procedure to measure the aspects of case-report quality that are relevant in the present context. However, for BLDA, validity was assessed by comparing scores with the judgement of neuroimaging experts: traceable vs. untraceable. BLDA≥3, which was the *a priori* definition of eligible images to be used in further analyses, was considered as the criterion to differentiate traceable from untraceable lesions. We found that BLDA correctly classified 96.3% of cases, with sensitivity of 100% and specificity of 94.2% (AUC of 0.97, asymptotic normal confidence interval 0.95-0.99), which is considered strong/excellent(5, 6). Inter-rater reliability was available for both CQA and BLDA since data collection was performed in parallel by two researchers: for CQA there was 86.5% agreement between the two raters (Cohen’s Kappa=0.81) while for BLDA there was 89.2% agreement (Cohen’s Kappa=0.84), which is considered excellent(7, 8).

**Table S3 - Summary of all articles and corresponding reported case(s) (9-122)**

| **Author** | **Title** | **Year** | **Article Type** | **Case No** | **Age (MM)** | **Gender** | **Hand** | **Time E-MM** | **MM**  **Duration** | **F/U**  **Time** | **MM**  **Recur.** | **Depr.**  **Recur.** | **Image** | **Etiology** | **Depr.** | **NP Family** | **A or S** | **CQA** | **BLDA** |
| --- | --- | --- | --- | --- | --- | --- | --- | --- | --- | --- | --- | --- | --- | --- | --- | --- | --- | --- | --- |
| Alla, P. | Hemiballism with manic access caused by toxoplasmic abscess in AIDS | 1997 | Case Report |  | 31 | Male |  | 2m | days | 8m | 0 | 0 | MRI | Other |  |  |  | 4 | 4 |
| Alpers, Bernard J | Relation of the hypothalamus to disorders of personality: report of a case | 1937 | Case Report |  | 37 | Male |  |  | 1y |  |  |  | Autopsy | Tumor |  |  |  | 2 | 2 |
| Antelmi, E. | Late onset bipolar disorder due to a lacunar state | 2014 | Case Report |  | 50 | Female |  | 6m | 6m | 9m |  |  | MRI | Vascular | 0 | 0 |  | 5 | 5 |
| Asghar-Ali, Ali A | Pure neuropsychiatric presentation of multiple sclerosis | 2004 | Clinical Case Conf. | #1 | 54 | Female |  |  |  |  |  |  | MRI | Other |  |  |  | 2 | 4 |
| Asghar-Ali, Ali A | Pure neuropsychiatric presentation of multiple sclerosis | * | Clinical Case Conf. | #2 | 50 | Female |  |  |  |  |  |  | MRI | Other | 0 |  |  | 3 | 4 |
| Avery, TL | Seven cases of frontal tumour with psychiatric presentation | 1971 | Case Report | #1 | 47 | Male |  |  |  |  |  |  |  | Tumor | 1 |  |  | 3 | 1 |
| Avery, TL | Seven cases of frontal tumour with psychiatric presentation | * | Case Report | #2 | 56 | Female |  |  |  | 6y |  |  |  | Tumor |  |  |  | 2 | 1 |
| Avery, TL | Seven cases of frontal tumour with psychiatric presentation | * | Case Report | #7 | 58 | Male |  |  |  | 2y |  |  |  | Tumor |  | 1 | 1 | 3 | 1 |
| Bakchine, S. | Manic-like state after bilateral orbitofrontal and right temporoparietal injury: efficacy of clonidine | 1989 | Case Report |  | 44 | Female | Right | 2w | 135d | 20m | 0 | 0 | CT | TBI | 1 | 0 |  | 5 | 3 |
| Bamrah, J. S. | Bipolar affective disorder following head injury | 1991 | Case Report |  | 58 | Male |  | 27y | 3w | 2y | 0 | 0 | CT | TBI | 0 | 0 |  | 5 | 3 |
| Barczak, P | Hypomania following complex partial seizures. A report of three cases | 1988 | Case Report | #1 | 54 | Male |  | 42y | 10d | 18m | 0 | 0 |  | Other |  |  |  | 3 | 1 |
| Barczak, P | Hypomania following complex partial seizures. A report of three cases | * | Case Report | #3 | 45 | Male |  |  |  | 5y | 1 | 1 |  | Other |  |  |  | 2 | 1 |
| Belli, H. | Solitary lesion in ponto-mesencephalic area related secondary mania: a case report | 2012 | Case Report |  | 62 | Male |  |  | 3w | 2m | 0 | 0 | MRI | Vascular | 0 | 0 |  | 5 | 4 |
| Bengesser, S. A. | Poststroke-bipolar affective disorder | 2013 | Case Report |  | 23 | Female |  | Few weeks |  | 1.5y | 1 | 1 |  | Vascular | 0 | 0 |  | 5 | 1 |
| Benjamin, Sheldon | Hypomania from left frontal AVM resection | 2000 | Case Report |  | 41 | Male | Right | 1w |  | 1y | 1 | 0 | MRI | Vascular | 0 |  |  | 4 | 5 |
| Benke, T. | Mania caused by a diencephalic lesion | 2002 | Case Report |  | 38 | Male |  | 48h | 8w | 2m | 0 | 0 | MRI | Vascular | 0 | 0 |  | 5 | 4 |
| Berthier, M. L. | Post-stroke rapid cycling bipolar affective disorder | 1992 | Case Report |  | 44 | Male |  | 2m | 1y | 1y |  |  |  | Vascular | 0 | 1 |  | 5 | 1 |
| Berthier, M. L. | Poststroke bipolar affective disorder: clinical subtypes, concurrent movement disorders, and anatomical correlates | 1996 | Case Series | #1 |  | Male | Right | 8,6±7m |  | 1-20y | 1 | 1 | Tracing | Vascular | 0 | 0 |  | 3 | 4 |
| Berthier, M. L. | Poststroke bipolar affective disorder: clinical subtypes, concurrent movement disorders, and anatomical correlates | * | Case Series | #2 |  | Male | Right | * | 2d | * | * | * | Tracing | Vascular | 1 | 0 |  | 3 | 4 |
| Berthier, M. L. | Poststroke bipolar affective disorder: clinical subtypes, concurrent movement disorders, and anatomical correlates | * | Case Series | #3 |  | Female | Right | * |  | * | * | * | Tracing | Vascular | 1 | 0 |  | 3 | 4 |
| Berthier, M. L. | Poststroke bipolar affective disorder: clinical subtypes, concurrent movement disorders, and anatomical correlates | * | Case Series | #4 |  | Female | Right | * |  | * | * | * | Tracing | Vascular | 0 | 0 |  | 3 | 4 |
| Berthier, M. L. | Poststroke bipolar affective disorder: clinical subtypes, concurrent movement disorders, and anatomical correlates | * | Case Series | #7 |  | Female | Right | * |  | * | * | * | MRI | Vascular | 0 | 0 |  | 3 | 5 |
| Berthier, M. L. | Poststroke bipolar affective disorder: clinical subtypes, concurrent movement disorders, and anatomical correlates | * | Case Series | #8 |  | Male | Right | * |  | * | * | * | MRI | Vascular | 0 | 1 | 1 | 3 | 5 |
| Berthier, M. L. | Poststroke bipolar affective disorder: clinical subtypes, concurrent movement disorders, and anatomical correlates | * | Case Series | #9 |  | Female | Right | * |  | * | * | * | Tracing | Vascular | 0 | 0 |  | 3 | 4 |
| Bhanji, S. | Aqueduct stenosis and manic-depressive psychosis | 1983 | Case Report |  | 24 | Male |  |  |  |  | 0 | 0 |  | Tumor | 0 | 0 |  | 4 | 1 |
| Bhatia, M. S. | Colloid cyst presenting as recurrent mania | 2013 | Case Report |  | 24 | Female |  |  | 15d | 6y | 1 |  | CT | Tumor | 0 | 0 |  | 4 | 4 |
| Binder, R. L. | Neurologically silent brain tumors in psychiatric hospital admissions: three cases and a review | 1983 | Case Report | #2 | 40 | Female |  |  | 10m | 13m | 0 | 0 |  | Tumor | 0 | 0 |  | 4 | 1 |
| Bobo, W. V. | Recurring episodes of Bell's mania after cerebrovascular accident | 2009 | Case Report |  | 54 | Female |  |  | several days | <5y | 1 | 1 |  | Vascular | 0 | 0 |  | 4 | 1 |
| Bogousslavsky, J. | Manic delirium and frontal-like syndrome with paramedian infarction of the right thalamus | 1988 | Case Report |  | 72 | Female | Right | im. | 2m | 6m | 0 | 0 | CT | Vascular | 0 | 0 |  | 5 | 3 |
| Bornke, C. | Acute mania due to a right hemisphere infarction | 1998 | Case Report |  | 67 | Female | Right |  |  |  | 0 | 0 | CT | Vascular | 0 | 0 |  | 4 | 4 |
| Brooks, J. O. | Secondary mania in older adults | 2005 | Clinical Case Conf. | #2 | 60 | Male |  |  | >4d |  | 0 | 0 | MRI | Tumor | 1 | 1 |  | 4 | 5 |
| Caeiro, L | Mania no AVC agudo | 2002 | Prosp. Obs. Study | #1 | 69 | Male |  | im. |  |  |  |  |  | Vascular | 1 |  |  | 3 | 1 |
| Caeiro, L. | Neuropsychiatric disturbances in acute subarachnoid haemorrhage | 2011 | Prosp. Obs. Study | #1 |  | Male |  | <4d |  |  |  |  |  | Vascular | 0 |  |  | 4 | 1 |
| Caeiro, L. | Neuropsychiatric disturbances in acute subarachnoid haemorrhage | * | Prosp. Obs. Study | #2 |  | Female |  | <4d |  |  |  |  |  | Vascular | 0 |  |  | 4 | 1 |
| Calo, J. J. P. | Mania after Traumatic Brain Injury - a Report of 2 Cases and 194 Literature-Review | 1994 | Case Report | #1 | 20 | Female |  | 57m | 9d | 2y | 0 | 0 | CT | TBI | 0 | 0 |  | 5 | 2 |
| Calo, J. J. P. | Mania after Traumatic Brain Injury - a Report of 2 Cases and 194 Literature-Review | * | Case Report | #2 | 67 | Male |  | 1m |  |  |  |  | CT | TBI | 0 | 0 |  | 5 | 3 |
| Camden, J.R. | Manic Behaviour Resulting From Left Frontal Closed Head Injury in an Adult With Fetal Alcohol Syndrome | 2007 | Case Report |  | 19 | Male |  | 2m |  |  |  |  | CT | TBI | 0 | 0 |  | 5 | 4 |
| Carran, M. A. | Mania following temporal lobectomy | 2003 | Retro. Obs. Study |  | 32.5±9.3 | 7F/9M |  | Within 1st y | 3 within weeks;6 <6m; 3 <1y; 3≥1y | 0-10y | 1 w/ rec. Manic ep. |  |  | Other | 3 |  |  | 4 | 1 |
| Celik, Y. | Post-stroke mania in late life due to right temporoparietal infarction | 2004 | Case Report |  | 69 | Female | Right | im. | 3w | 6m | 0 | 0 |  | Vascular | 0 | 0 |  | 5 | 1 |
| Chimowitz, MI | Resolution of psychotic depression after right temporoparietal infarction | 1990 | Case Report |  | 70 | Male |  |  | 2d | 6m | 0 | 0 |  | Vascular | 0 | 0 |  | 4 | 1 |
| Clark, A. F. | Mania following head injury. A report of two cases and a review of the literature | 1987 | Case Report | #2 | 60 | Male |  | 2m | >3w | 1.5y | 1 |  |  | TBI | 0 | 0 |  | 5 | 1 |
| Claude, H. | Manic Excitation and Cerebral Tumor | 1928 | Case Report |  | 52 | Female |  |  | 128d |  |  |  | Autopsy | Tumor | 0 | 0 |  | 4 | 3 |
| Cohen, M. R. | Localized right cerebral hemisphere dysfunction and recurrent mania | 1980 | Case Report |  | 61 | Male | Right | 3y | 71d | 15m | 1 |  |  | Vascular | 0 | 0 |  | 5 | 1 |
| Danel, T. | Mood disorders and right hemisphere infarction | 1989 | Case Report | #2 | 57 | Male | Right | 11m |  | 13m | 1 | 1 | CT | Vascular | 0 |  |  | 3 | 3 |
| Daniels, J. P. | Quetiapine treatment for mania secondary to brain injury in 2 patients | 2008 | Case Report | #1 | 27 | Male |  | 6w | 5w |  |  |  |  | TBI | 0 | 0 |  | 5 | 1 |
| Das, P. | Late-onset recurrent mania as a manifestation of Wallenberg syndrome: a case report and review of the literature | 2015 | Case Report |  | 86 | Male |  | 1m |  | 4y | 1 |  | MRI | Vascular | 0 | 0 |  | 5 | 5 |
| Dauncey, K | Mania in the early stages of AIDS | 1988 | Case Report |  | 37 | Male |  |  |  | 6m |  |  |  | Other | 0 | 0 |  | 4 | 1 |
| Drake, M. E., Jr. | Secondary mania after ventral pontine infarction | 1990 | Case Report | #1 | 52 | Male | Right |  |  | 18m | 0 | 0 |  | Vascular | 0 |  |  | 4 | 1 |
| Drake, M. E., Jr. | Secondary mania after ventral pontine infarction | * | Case Report | #2 | 56 | Male | Right |  |  | 12m | 0 | 0 |  | Vascular | 0 |  |  | 3 | 1 |
| El Hechmi, S. | Bipolar disorder in the aftermath of a traumatic brain injury: report of a case | 2013 | Case Report |  | 36 | Female |  | Months |  | 5y | 1 |  |  | TBI | 0 | 0 |  | 5 | 1 |
| Estrade, J. F. | Secondary mania. Diagnostic problems (apropos of a case of secondary mania in partial complex epilepsy crisis) | 1989 | Case Report |  | 52 | Female | Right | 9y |  | 31.53m | 1 |  |  | Other | 0 | 1 | 1 | 5 | 1 |
| Fawcett, R. G. | Cerebral infarct presenting as mania | 1991 | Case Report |  | 61 | Male | Right |  |  | 3y | 0 | 0 |  | Vascular | 0 | 0 |  | 4 | 1 |
| Fenn, D. | Post-stroke mania late in life involving the left hemisphere | 1999 | Case Report |  | 78 | Male | Right |  | 2.75m | 2m | 0 | 0 |  | Vascular | 0 | 0 |  | 4 | 1 |
| Filley, C. M. | Neurobehavioral presentations of brain neoplasms | 1995 | Case Series | #6 | 56 | Female | Right |  |  |  |  |  | MRI | Tumor | 1 |  |  | 2 | 4 |
| Gafoor, R. | Three case reports of secondary mania: evidence supporting a right frontotemporal locus | 2003 | Case Report | #1 | 72 | Male |  |  |  |  |  |  |  | Vascular |  |  |  | 2 | 1 |
| Gafoor, R. | Three case reports of secondary mania: evidence supporting a right frontotemporal locus | * | Case Report | #2 | 65 | Male |  |  |  |  |  |  |  |  |  |  |  | 2 | 1 |
| Gafoor, R. | Three case reports of secondary mania: evidence supporting a right frontotemporal locus | * | Case Report | #3 | 35 | Male |  |  |  |  |  |  |  | Vascular |  |  |  | 2 | 1 |
| Gal, Paul | Mental symptoms in cases of tumor of temporal lobe | 1958 | Case Series | #2 | 40 | Male |  |  |  |  |  |  |  | Tumor |  |  |  | 2 | 1 |
| Galindo Menendez, A. | Parenchymal neurosyphilis. Insidious onset (dementia) and acute onset (manic type) forms | 1996 | Case Report | #2 | 45 | Male |  | 2w | 3.25m |  |  |  |  | Other | 0 |  |  | 4 | 1 |
| Garland, E. J. | Multiple-Sclerosis and Affective-Disorders | 1991 | Case Report | #2 | 30 | Male |  |  | several weeks | 6m |  | 1 |  | Other | 1 | 1 | 1 | 4 | 1 |
| Goyal, R. | Mania secondary to right-sided stroke - responsive to olanzapine | 2006 | Case Report |  | 51 | Male |  | 1m | 11w | 12m | 0 | 0 |  | Vascular | 0 | 1 |  | 5 | 1 |
| Greenberg, D. B. | Mania resulting from brain stem tumor | 1985 | Case Report |  | 55 | Male | Left |  | 4w | 9m | 0 | 0 |  | Tumor |  |  |  | 2 | 1 |
| Haq, M. Z. | Bipolar disorder and tuberous sclerosis complex: is it a mere coincidence? | 2009 | Case Report |  | 26 | Female |  |  | 4m | 5m | 0 | 0 | CT | Other | 0 |  |  | 3 | 4 |
| Heinrich, T. W. | Recurrent mania associated with repeated brain injury | 2004 | Case Report |  | 69 | Male |  | 18m | 3w | 2m | 0 | 0 |  | TBI | 0 |  |  | 4 | 1 |
| Huffman, J. | Acute psychiatric manifestations of stroke: a clinical case conference | 2003 | Case Report | #4 | 57 | Male |  |  | 19d |  |  |  |  | Vascular | 1 | 1 | 1 | 4 | 1 |
| Hunt, N. | Seasonal affective disorder following brain injury | 1990 | Case Report |  |  | Female |  |  |  | 3y | 1 | 1 |  | Vascular | 1 | 0 |  | 3 | 3 |
| Inzelberg, R. | Acute mania and hemichorea | 2001 | Case Report |  | 61 | Male | Right | im. | 3w | 2w | 0 | 0 | MRI | Vascular | 0 |  |  | 3 | 2 |
| Isles, L. J. | Secondary Mania after Open-Heart-Surgery | 1991 | Case Report |  | 54 | Male |  | im. | 5w | 4w | 0 | 0 |  | Vascular | 0 | 0 |  | 5 | 1 |
| Jagadesan, V. | Cerebellar Stroke-manifesting as Mania | 2014 | Case Report |  | 28 | Male |  |  | 1m |  |  |  | CT | Vascular | 0 | 0 |  | 3 | 2 |
| Jamieson, R. C. | Manic psychosis in a patient with multiple metastatic brain tumors | 1979 | Case Report |  | 45 | Male |  |  | 8m | 2m | 0 | 0 |  | Tumor | 0 | 1 |  | 4 | 1 |
| Jampala, V. C. | Mania secondary to left and right hemisphere damage | 1983 | Case Report | #1 | 24 | Male | Right | 10m |  | 28y | 1 |  |  | Vascular | 0 | 0 |  | 5 | 1 |
| Julayanont, P. | Behavioral Disconnection Syndrome Manifesting as Combined Mania and Visual-Auditory Hallucinations Secondary to Isolated Right Thalamic Hemorrhage | 2017 | Case Report |  | 55 | Male |  | 7d | 4m | 1y | 0 |  | MRI | Vascular | 0 |  |  | 4 | 5 |
| Kanemoto, K | Hypomania after temporal lobectomy: a sequela to the increased excitability of the residual temporal lobe? | 1995 | Case Report |  | 33 | Male |  | Several days | 2m | 12m | 0 | 0 |  | Other |  |  |  | 3 | 1 |
| Kar, S. K. | Mood Disorder as an Early Presentation of Epidermoid of Quadrigeminal Cistern | 2017 | Case  Report |  | 32 | Male |  |  |  | 8w | 0 | 0 | MRI | Tumor | 1 |  |  | 3 | 5 |
| Koreki, A. | Increased left anterior insular and inferior prefrontal activity in post-stroke mania | 2012 | Case Report |  | 68 | Male | Right | 6w | 2m | 2y | 0 | 0 | CT | Vascular | 0 | 0 |  | 5 | 4 |
| Kotrla, K. J. | A case of organic mania associated with open heart surgery | 1994 | Case Report |  | 62 | Male | Left | 8m | 7d | 8m | 1 |  |  | Vascular | 1 | 0 |  | 5 | 1 |
| Ku, B. D. | Secondary mania in a patient with delayed anoxic encephalopathy after carbon monoxide intoxication | 2006 | Case Report |  | 55 | Female | Right | 35d | 15d | 100d | 0 | 0 | MRI | Other | 0 |  |  | 4 | 5 |
| Kulisevsky, J. | Hemiballismus and Secondary Mania Following a Right Thalamic Infarction | 1993 | Case Report |  | 81 | Female | Right | <3d | 1m | 3m | 0 | 0 | MRI | Vascular | 0 |  |  | 3 | 4 |
| Kumar, S. K. | CADASIL presenting as bipolar disorder | 1997 | Case Report |  | 55 | Male |  |  |  |  |  |  |  | Vascular | 0 |  |  | 3 | 1 |
| Lauterbach, E. C. | Bipolar disorders, dystonia, and compulsion after dysfunction of the cerebellum, dentatorubrothalamic tract, and substantia nigra | 1996 | Case Report | #3 | 27 | Male |  |  |  |  | 1 | 1 |  | TBI | 1 | 0 |  | 4 | 1 |
| Lee, Y. M. | Secondary mania in a patient with solitary red nucleus lesion | 2014 | Case Report |  | 59 | Male |  | 18d | 53d | 2m | 0 | 0 |  | Vascular | 0 |  |  | 4 | 1 |
| Leibson, E. | Anosognosia and mania associated with right thalamic haemorrhage | 2000 | Case Report |  | 53 | Male | Right | 1w | 9w |  | 0 | 0 |  | Vascular | 0 |  |  | 4 | 1 |
| Liu, C. Y. | Bipolar disorder following a stroke involving the left hemisphere | 1996 | Case Report |  | 48 | Male | Right | 4m | 2w | 2m | 0 | 0 | MRI | Vascular | 0 | 0 |  | 5 | 4 |
| Lupo, M. | Evidence of Cerebellar Involvement in the Onset of a Manic State | 2018 | Case  Report |  | 43 | Female | Right | 10m |  |  |  |  | MRI | Vascular |  |  |  | 3 | 5 |
| Malamud, Nathan | Psychiatric disorder with intracranial tumors of limbic system | 1967 | Case Series | #16 | 53 | Male |  |  |  |  |  |  |  | Tumor |  |  |  | 2 | 1 |
| Mark, Mordechi | Bipolar disorder associated with an acoustic neurinoma | 1991 | Case Report |  | 26 | Male |  |  |  | 15y | 1 | 1 |  | Tumor | 1 |  |  | 3 | 1 |
| McKeown, S. P. | Mania following head injury | 1987 | Case Report |  | 38 | Male |  | 3w |  |  |  |  |  | TBI | 0 | 0 |  | 5 | 1 |
| Miller, B. L. | Hypersexuality or altered sexual preference following brain injury | 1986 | Case Series | #4 | 31 | Female |  | >5d | 1m |  |  |  | CT | Vascular |  |  |  | 3 | 2 |
| Modrego, P. J. | Familial multiple sclerosis with repetitive relapses of manic psychosis in two patients (mother and daughter) | 2000 | Case Report | #2 | 19 | Female |  | 2m | 2m | 1y | 1 |  | MRI | Other | 0 | 1 | 1 | 5 | 4 |
| Mumoli, N. | Frontal lobe syndrome caused by a giant meningioma presenting as depression and bipolar disorder | 2013 | Case Report |  | 55 | Male |  |  | >8m | 15m | 1 |  | MRI | Tumor | 1 | 0 |  | 4 | 4 |
| Murai, T. | Rapid cycling bipolar disorder after left temporal polar damage | 2003 | Case Report |  | 48 | Female |  | 6w | 3w | 1y | 1 | 1 | MRI | TBI | 0 |  |  | 4 | 4 |
| Mustafa, B. | Secondary mania following traumatic brain injury | 2005 | Case Report |  | 35 | Female |  | 23d |  |  |  |  |  | TBI | 0 |  |  | 4 | 1 |
| Nagaratnam, N | Aberrant sexual behaviour following stroke | 1998 | Case Series | #2 | 65 | Male |  | 1m |  |  |  |  |  | Vascular |  |  |  | 3 | 1 |
| Nagaratnam, N. | Secondary mania of vascular origin in elderly patients: a report of two clinical cases | 2006 | Case Report | #1 | 72 | Female | Right | 3y |  |  |  |  | CT | Vascular | 0 |  |  | 4 | 4 |
| Nagaratnam, N. | Secondary mania of vascular origin in elderly patients: a report of two clinical cases | * | Case Report | #2 | 80 | Male | Right |  |  |  |  |  | CT | Vascular | 0 | 0 |  | 4 | 4 |
| Nizamie, S. H. | Mania following head injury: case reports and neuropsychological findings | 1988 | Case Report | #2 | 55 | Male |  | 7m | 3m |  | 0 | 0 |  | TBI | 0 | 0 |  | 5 | 1 |
| OH, Koh | A Case of Post-Stroke Mania | 2010 | Case Report |  | 72 | Female |  | 3d |  |  |  |  |  | Vascular | 0 | 0 |  | 5 | 1 |
| Okun, Michael S | Transient manic behavior after pallidotomy | 2003 | Case Report | #1 | 72 | Female |  | im. | 1w |  |  |  | MRI | Other |  | 0 |  | 4 | 4 |
| Okun, Michael S | Transient manic behavior after pallidotomy | * | Case Report | #2 | 56 | Female |  | im. | 1m |  |  |  | MRI | Other |  | 0 |  | 4 | 4 |
| Oppler, Willy | Manic psychosis in a case of parasagittal meningioma | 1950 | Case Report |  | 29 | Male |  |  | 5m | 4m | 0 | 0 |  | Tumor | 0 |  |  | 3 | 1 |
| Park, Soyeon | Case report: bipolar disorder as the first manifestation of CADASIL | 2014 | Case Report |  | 48 | Female |  |  |  |  |  |  |  | Vascular | 1 | 1 | 1 | 4 | 1 |
| Pathak, Abhishek | Post-stroke mania–a case report | 2014 | Case Report |  | 65 | Male |  | 2d | 10d |  |  |  | CT | Vascular | 0 | 0 |  | 5 | 3 |
| Reisch, T. | A case of hydrocephalus occlusus presenting as bipolar disorder | 2005 | Case Report |  | 24 | Male |  |  |  | 2.5y | 0 | 0 | MRI | Other | 1 |  |  | 3 | 5 |
| Robinson, R. G. | Mood disorders in stroke patients | 1984 | Case Series | #1 |  |  | Right |  |  |  |  |  |  | Vascular |  |  |  | 0 | 1 |
| Robinson, R. G. | Mood disorders in stroke patients | * | Case Series | #2 |  |  | Right |  |  |  |  |  |  | Vascular |  |  |  | 0 | 1 |
| Robinson, R. G. | Mood disorders in stroke patients | * | Case Series | #3 |  |  | Right |  |  |  |  |  |  | Vascular |  |  |  | 0 | 1 |
| Robinson, R. G. | Mood disorders in stroke patients | * | Case Series | #4 |  |  | Right |  |  |  |  |  |  | Vascular |  |  |  | 0 | 1 |
| Robinson, R. G. | Mood disorders in stroke patients | * | Case Series | #5 |  |  | Right |  |  |  |  |  |  | Vascular |  |  |  | 0 | 1 |
| Robinson, R. G. | Mood disorders in stroke patients | * | Case Series | #6 |  |  | Right |  |  |  |  |  |  | Vascular |  |  |  | 0 | 1 |
| Robinson, R. G. | Comparison of mania and depression after brain injury: causal factors | 1988 | Case Series |  | 53.3 ± 20 | 8F/9M | 15R/2L | 5.8±7.2m |  |  |  |  | Tracing | 9V/6Tu/2TBI |  | 7 |  | 4 | 4 |
| Rocha, F. F. | A successful outcome with valproic acid in a case of mania secondary to stroke of the right frontal lobe | 2008 | Case Report |  | 47 | Female |  | 3w | 2m | 12m | 0 | 0 |  | Vascular | 0 | 0 |  | 5 | 1 |
| Rocha, F. F. | Poststroke manic symptoms: an unusual neuropsychiatric condition | 2008 | Case Report |  | 57 | Male |  |  | 2d |  | 1 |  | MRI | Vascular | 0 |  |  | 3 | 4 |
| Rosenbaum, A. H. | Positive therapeutic response to lithium in hypomania secondary to organic brain syndrome | 1975 | Case Report |  | 57 | Male |  | 4m | 72d | 6m | 1 |  |  | Other | 0 | 0 |  | 5 | 1 |
| Routh, Rajdeep | Post‐stroke mania: a rare but treatable presentation | 2014 | Case Report |  | 83 | Male |  | 2w | several weeks |  |  |  |  | Vascular | 0 | 0 |  | 5 | 1 |
| Salazar-Calderon Perriggo, V. H. | Silent solitary right parietal chondroma resulting in secondary mania | 1993 | Case Report |  | 27 | Female | Right |  | 4w | 2y |  |  | CT | Tumor |  |  |  | 3 | 3 |
| Sanders, Richard D | Hypergraphia and Secondary Mania in Temporal Lobe Epilepsy: Case Reports and Literature Review | 1994 | Case Report | #1 | 66 | Male | Right | 1y |  | 8y | 1 | 1 |  | Vascular | 0 | 0 |  | 5 | 1 |
| Semiz, M. | Case of organic mania associated with stroke and open-heart surgery | 2010 | Case Report |  | 27 | Female | Right | 1y | 3m |  |  |  |  | Vascular | 0 | 0 |  | 5 | 1 |
| Semiz, U. B. | Leptospirosis presenting with mania and psychosis: Four consecutive cases seen in a military hospital in Turkey | 2005 | Case Report | #1 | 21 | Male |  |  |  |  |  |  |  | Other | 0 |  |  | 3 | 1 |
| Sidhom, Youssef | Bipolar Disorder and Multiple Sclerosis: A Case Series | 2014 | Case Series | #3 | 23 | Female |  |  |  |  |  |  | MRI | Other | 0 | 1 | 1 | 4 | 4 |
| Starkstein, S. E. | Mania after brain injury. A controlled study of causative factors | 1987 | Controlled Study |  | 50±20 | 4F/7M | 9R/2L | 10m±9m |  |  |  |  | Tracing | 4V/4Tu/2TBI/1O | 1 | 5 | 3 | 5 | 4 |
| Starkstein, S. E. | Mechanisms of mania after brain injury. 12 case reports and review of the literature | 1988 | Case Series | #1 | 66 | Male | Right | 1y |  |  |  |  | CT | Vascular | 0 | 1 | 1 | 5 | 3 |
| Starkstein, S. E. | Mechanisms of mania after brain injury. 12 case reports and review of the literature | * | Case Series | #2 | 27 | Male | Right | 2m | 3w |  | 1 | 1 |  | Vascular | 0 | 1 | 1 | 5 | 1 |
| Starkstein, S. E. | Mechanisms of mania after brain injury. 12 case reports and review of the literature | * | Case Series | #4 | 35 | Female | Right |  |  |  |  |  | CT | Vascular | 0 | 1 | 1 | 4 | 3 |
| Starkstein, S. E. | Mechanisms of mania after brain injury. 12 case reports and review of the literature | * | Case Series | #5 | 63 | Male | Right | 4w |  |  |  |  | CT | Other | 0 | 0 |  | 5 | 3 |
| Starkstein, S. E. | Mechanisms of mania after brain injury. 12 case reports and review of the literature | * | Case Series | #6 | 61 | Female | Right | im. |  | 3.5y | 1 |  | CT | Other | 0 | 1 |  | 5 | 3 |
| Starkstein, S. E. | Mechanisms of mania after brain injury. 12 case reports and review of the literature | * | Case Series | #7 | 48 | Female | Right |  |  |  | 1 |  | CT | Tumor | 0 | 1 |  | 4 | 3 |
| Starkstein, S. E. | Mechanisms of mania after brain injury. 12 case reports and review of the literature | * | Case Series | #8 | 54 | Female | Right |  |  |  | 1 | 0 |  | Tumor | 0 | 0 |  | 4 | 1 |
| Starkstein, S. E. | Mechanisms of mania after brain injury. 12 case reports and review of the literature | * | Case Series | #9 | 28 | Male | Amb. | im. |  | 1y | 1 |  | CT | Other | 0 | 0 |  | 5 | 3 |
| Starkstein, S. E. | Mechanisms of mania after brain injury. 12 case reports and review of the literature | * | Case Series | #10 | 74 | Male | Left | im. |  | 8y | 1 | 1 |  | Other | 0 | 0 |  | 5 | 1 |
| Starkstein, S. E. | Mania after brain injury: neuroradiological and metabolic findings | 1990 | Case Series | #1 | 37 | Male | 7R/1L | 6d |  |  |  |  | MRI | TBI | 0 | 0 |  | 5 | 4 |
| Starkstein, S. E. | Mania after brain injury: neuroradiological and metabolic findings | * | Case Series | #2 | 55 | Male | 7R/1L | 4wk |  |  |  |  | CT | Vascular | 0 | 0 |  | 5 | 3 |
| Starkstein, S. E. | Mania after brain injury: neuroradiological and metabolic findings | * | Case Series | #3 | 79 | Female | 7R/1L | im. |  | 6m | 1 |  | CT | Vascular | 0 | 0 |  | 5 | 3 |
| Starkstein, S. E. | Mania after brain injury: neuroradiological and metabolic findings | * | Case Series | #4 | 49±17 (25-79) | Male | 7R/1L | 2-im.; 2m; 6m; 24m |  |  |  |  | CT | Vascular | 0 | 0 |  | 5 | 3 |
| Starkstein, S. E. | Mania after brain injury: neuroradiological and metabolic findings | * | Case Series | #5 | * | Male | 7R/1L | * |  |  |  |  | MRI | Vascular | 0 | 0 |  | 5 | 4 |
| Starkstein, S. E. | Mania after brain injury: neuroradiological and metabolic findings | * | Case Series | #6 | * | Male | 7R/1L | * |  |  |  |  | MRI | Vascular | 0 | 1 | 1 | 5 | 4 |
| Starkstein, S. E. | Mania after brain injury: neuroradiological and metabolic findings | * | Case Series | #7 | * | Male | 7R/1L | * |  |  |  |  | CT | Vascular | 0 | 0 |  | 5 | 3 |
| Starkstein, S. E. | Mania after brain injury: neuroradiological and metabolic findings | * | Case Series | #8 | * | Male | 7R/1L | * |  |  |  |  | CT | TBI | 0 | 0 |  | 5 | 3 |
| Starkstein, S. E. | Manic-depressive and pure manic states after brain lesions | 1991 | Controlled Study |  | 61.2±15.9 | 3F/4M | 6R/1L | 3-2w; 1-7w; 1-3m;  1-6m; 1-ND |  |  |  |  | Tracing | 6V/1O | 1 | 2 | 1 | 5 | 4 |
| Starkstein, S. E. | Manic-depressive and pure manic states after brain lesions | * | Controlled Study |  | 50.2±16.9 | 4F/8M | 10R/2L | 5-1w; 1-18m; 1-2y; 1-4y; 4-im. |  |  |  |  | Tracing | 5V/5Tu/2TBI | 0 | 3 | 3 | * | * |
| Stern, K | Glioma of the diencephalon in a manic patient | 1942 | Case Report |  | 30 | Female |  |  | 9y |  |  |  | Tracing | Tumor | 0 | 1 | 1 | 4 | 3 |
| Sullivan, G. | Secondary Mania Following Cerebral Hypoxia | 1995 | Case Report |  | 27 | Male |  | 5y |  |  |  |  |  | Other | 0 | 0 |  | 5 | 1 |
| Sweet, Robert A | Case of craniopharyngioma in late life | 1990 | Case Report |  | 72 | Male |  |  |  |  |  |  |  | Tumor |  |  |  | 2 | 1 |
| Taylor, J. B. | Case 21-2018: A 61-Year-Old Man with Grandiosity, Impulsivity, and Decreased Sleep | 2018 | Case  Report |  | 61 | Male |  |  | 8m |  |  |  | MRI | Vascular | 1 | 1 |  | 4 | 5 |
| Topcuoglu, Volkan | Mood Disorder due to Herpes Simplex Encephalitis with Neuroimaging Findings Limited to the Right Hemisphere and Cerebellum: Case Report | 2012 | Case Report |  | 62 | Male |  | 4.5m | 5m | 7y | 0 | 0 | MRI | Other | 0 |  |  | 3 | 4 |
| Trillet, M. | Hemiballismus with logorrhea and thymo-affective disinhibition caused by hematoma of the left subthalamic nucleus | 1995 | Case Report |  | 71 | Male | Right | 2-3d |  |  |  |  | MRI | Vascular | 0 | 0 |  | 5 | 4 |
| Trimble, Michael R | Neuropsychiatric disturbances following brainstem lesions | 1981 | Case Report | #2 | 21 | Female |  | 1y |  | 5y |  |  |  | Vascular |  |  |  | 3 | 1 |
| Turecki, G. | Bipolar disorder following a left basal-ganglia stroke | 1993 | Case Report |  | 47 | Male | Right | im. |  |  | 0 | 0 |  | Vascular | 0 | 0 |  | 5 | 1 |
| Vidrih, B. | Arachnoid cyst as the cause of bipolar affective disorder: case report | 2012 | Case Report |  | 20 | Male |  |  |  | 7.5y |  | 1 |  | Tumor | 0 | 0 |  | 4 | 1 |
| Wright, M. T. | Bipolar syndromes following brain trauma | 1997 | Case Report |  | 46 | Male | Left | 14y and 9 y |  |  | 1 | 1 | MRI | TBI | 1 |  |  | 4 | 4 |
| Ybarra, Mariana Ines | Bipolar disorder and multiple sclerosis | 2007 | Case Report | #2 | 43 | Male |  |  |  |  |  | 1 |  | Other |  |  |  | 2 | 1 |
| Ybarra, Mariana Ines | Bipolar disorder and multiple sclerosis | * | Case Report | #3 | 38 | Female |  |  |  |  | 1 |  |  | Other | 1 |  |  | 3 | 1 |
| Yetimalar, Y. | Secondary mania after pontin cavernous angioma | 2007 | Case Report |  | 34 | Male |  |  | 3.75m |  |  |  |  | Tumor | 0 | 0 |  | 4 | 1 |
| Zincir, S. B. | Mania secondary to traumatic brain injury: a case report | 2014 | Case Report |  | 42 | Male |  | 3y | >20d | 2m | 0 | 0 |  | TBI | 0 | 0 |  | 5 | 1 |

**1st** – first; **A** – Affective Disorder; **Amb**. – Ambidextrous; **BLDA** – Brain Lesion Documentation Assessment; Conf. – Conference; **CQA** – Clinical Quality Assessment; **CT** – Computerized Tomography; **d** – day; **Depr**. – Depression; **E** – Event causing brain insult; **E-MM** – time between event causing brain insult and the manic/mixed state episode onset; **F** – Female; **F/U** – Follow-up time after mania resolution; **h** – hours; **im**. – immediate = 0; **L** – Left; **M** – Male; **m** – Month; **MM** – manic/mixed state episode onset; **MRI** – Magnetic Resonance Imaging; **ND** – Not Defined; **NP** – Neuropsychiatric; **O** – Other; **Obs**. – Observational; **Prev**. – Previous; **Prosp**. – Prospective; **R** – Right; **Recur. –** Recurrence of affective episode; **Retro.** – Retrospective; **S** – Suicide; **Tu** – Tumor; **TBI** – Traumatic Brain Injury; **V** – Vascular; **w** – week; **y** – year.

* The above cell contains the information about the group

**Table S4 –** Manic syndrome classification table

| **Author** | **Year** | **Case #** | **DSM 5 Criteria** | | | | | | | | | **Psy.** | **DSM 5** | | **DSM 4TR** | **DSM 4** | **DSM 3TR** | **DSM 3** | **ICD 10** | **MAS** | **PSE** | **Uns.** |
| --- | --- | --- | --- | --- | --- | --- | --- | --- | --- | --- | --- | --- | --- | --- | --- | --- | --- | --- | --- | --- | --- | --- |
|  |  |  | **A** | **B1** | **B2** | **B3** | **B4** | **B5** | **B6** | **B7** | **C^a^** |  | **Full  Crit.^b^** | **Core ^c^** |  |  |  |  |  |  |  |  |
| Alla, P. | 1997 | #1 | 1 |  | 1 | 1 |  |  | 1 |  |  |  |  | 1 |  |  |  |  |  |  |  | 1 |
| Alpers, Bernard J | 1937 | #1 | 1 |  | 1 |  |  | 1 | 1 |  |  |  |  | 1 |  |  |  |  |  |  |  | 1 |
| Antelmi, E. | 2014 | #1 | 1 |  | 1 |  |  | 1 | 1 |  |  |  |  | 1 | 1 |  |  |  |  |  |  |  |
| Asghar-Ali, Ali A | 2004 | #1 | 1 | 1 |  | 1 |  | 1 | 1 |  | 1 | 1 | 1 | 1 |  |  |  |  |  |  |  |  |
| Asghar-Ali, Ali A | 2004 | #2 |  |  |  | 1 | 1 |  | 1 | 1 | 1 | 1 |  |  |  |  |  |  |  |  |  | 1 |
| Avery, TL | 1971 | #1 | 1 |  |  |  |  |  |  |  |  |  |  |  |  |  |  |  |  |  |  | 1 |
| Avery, TL | 1971 | #2 | 1 |  |  |  |  | 1 |  |  |  |  |  |  |  |  |  |  |  |  |  | 1 |
| Avery, TL | 1971 | #7 | 1 | 1 |  |  |  |  | 1 | 1 | 1 | 1 | 1 | 1 |  |  |  |  |  |  |  |  |
| Bakchine, S. | 1989 | #1 | 1 |  |  | 1 | 1 | 1 | 1 |  | 1 | 1 | 1 | 1 |  |  |  |  |  |  |  |  |
| Bamrah, J. S. | 1991 | #1 | 1 | 1 | 1 | 1 |  |  | 1 | 1 | 1 | 1 | 1 | 1 |  |  |  |  |  |  |  |  |
| Barczak, P | 1988 | #1 | 1 |  | 1 | 1 | 1 |  | 1 |  | 1 | 1 | 1 | 1 |  |  |  |  |  |  |  |  |
| Barczak, P | 1988 | #3 | 1 |  |  | 1 |  |  | 1 | 1 | 1 | 1 | 1 | 1 |  |  |  |  |  |  |  |  |
| Belli, H. | 2012 | #1 | 1 | 1 | 1 | 1 | 1 |  | 1 | 1 | 1 | 1 | 1 | 1 |  |  |  |  |  |  |  |  |
| Bengesser, S. A. | 2013 | #1 |  |  |  |  |  |  |  |  |  |  |  |  |  |  |  |  |  |  |  | 1 |
| Benjamin, Sheldon | 2000 | #1 | 1 | 0 | 1 | 1 |  |  | 1 |  |  | 0 |  | 1 |  | 1 |  |  |  |  |  |  |
| Benke, T. | 2002 | #1 | 1 | 1 |  | 1 | 1 | 1 | 1 |  |  | 0 |  | 1 |  | 1 |  |  |  |  |  |  |
| Berthier, M. L. | 1992 | #1 | 1 |  |  | 1 | 1 | 1 | 1 | 1 |  |  |  | 1 |  |  |  |  |  | 1 |  |  |
| Berthier, M. L. | 1996 | #1 |  |  |  |  |  |  |  |  |  |  |  |  |  |  | 1 |  |  |  |  |  |
| Berthier, M. L. | 1996 | #2 |  |  |  |  |  |  |  |  |  |  |  |  |  |  | 1 |  |  |  |  |  |
| Berthier, M. L. | 1996 | #3 |  |  |  |  |  |  |  |  |  |  |  |  |  |  | 1 |  |  |  |  |  |
| Berthier, M. L. | 1996 | #4 |  |  |  |  |  |  |  |  |  |  |  |  |  |  | 1 |  |  |  |  |  |
| Berthier, M. L. | 1996 | #7 |  |  |  |  |  |  |  |  |  |  |  |  |  |  | 1 |  |  |  |  |  |
| Berthier, M. L. | 1996 | #8 |  |  |  |  |  |  |  |  |  |  |  |  |  |  | 1 |  |  |  |  |  |
| Berthier, M. L. | 1996 | #9 |  |  |  |  |  |  |  |  |  |  |  |  |  |  | 1 |  |  |  |  |  |
| Bhanji, S. | 1983 | #1 |  | 1 |  |  | 1 |  | 1 | 1 | 1 | 1 |  |  |  |  |  |  |  |  |  | 1 |
| Bhatia, M. S. | 2013 | #1 | 1 | 1 | 1 | 1 |  |  | 1 | 1 | 1 | 1 | 1 | 1 |  |  |  |  |  |  |  |  |
| Binder, R. L. | 1983 | #2 |  | 1 | 1 | 1 |  |  | 1 |  | 1 | 1 |  |  |  |  |  |  |  |  |  | 1 |
| Bobo, W. V. | 2009 | #1 | 1 |  | 1 | 1 |  |  | 1 |  | 1 | 1 | 1 | 1 |  |  |  |  |  |  |  |  |
| Bogousslavsky, J. | 1988 | #1 | 1 |  |  | 1 | 1 |  | 1 |  |  |  |  | 1 |  |  |  |  |  |  |  | 1 |
| Bornke, C. | 1998 | #1 | 1 | 1 | 1 | 1 | 1 |  | 1 |  | 1 | 1 | 1 | 1 |  |  |  |  |  |  |  |  |
| Brooks, J. O. | 2005 | #2 | 1 | 1 | 1 | 1 |  |  | 1 |  | 1 | 1 | 1 | 1 |  |  |  |  |  |  |  |  |
| Caeiro, L | 2002 | #1 |  |  |  |  |  |  |  |  |  |  |  |  |  |  |  |  |  | 1 |  |  |
| Caeiro, L. | 2011 | #1 |  |  |  |  |  |  |  |  |  |  |  |  | 1 |  |  |  |  |  |  |  |
| Caeiro, L. | 2011 | #2 |  |  |  |  |  |  |  |  |  |  |  |  | 1 |  |  |  |  |  |  |  |
| Calo, J. J. P. | 1994 | #1 | 1 | 1 | 1 |  | 1 | 1 | 1 | 1 | 1 |  | 1 | 1 |  |  |  |  |  |  |  |  |
| Calo, J. J. P. | 1994 | #2 | 1 | 1 | 1 | 1 | 1 |  | 1 | 1 | 1 |  | 1 | 1 |  |  |  |  |  |  |  |  |
| Camden, J.R. | 2007 | #1 | 1 | 0 | 1 | 1 |  |  | 1 |  |  | 0 |  | 1 |  |  |  |  |  |  |  | 1 |
| Carran, M. A. | 2003 | Group |  |  |  |  |  |  |  |  |  |  |  |  |  | 1 | 1 |  |  |  |  |  |
| Celik, Y. | 2004 | #1 | 1 | 1 | 1 | 1 | 1 |  | 1 |  | 1 | 1 | 1 | 1 |  |  |  |  |  |  |  |  |
| Chimowitz, MI | 1990 | #1 | 1 |  |  | 1 |  | 1 | 1 |  | 1 | 1 | 1 | 1 |  |  |  |  |  |  |  |  |
| Clark, A. F. | 1987 | #2 | 1 |  |  | 1 |  |  | 1 | 1 | 1 |  | 1 | 1 |  |  |  |  |  |  |  |  |
| Claude, H. | 1928 | #1 | 1 | 1 | 1 | 1 | 1 |  | 1 |  | 1 | 1 | 1 | 1 |  |  |  |  |  |  |  |  |
| Cohen, M. R. | 1980 | #1 | 1 | 1 | 1 | 1 | 1 |  | 1 | 1 | 1 | 0 | 1 | 1 |  |  |  |  |  |  |  |  |
| Danel, T. | 1989 | #2 |  |  |  |  |  |  |  |  |  |  |  |  |  |  |  |  |  |  |  | 1 |
| Daniels, J. P. | 2008 | #1 | 1 |  | 1 | 1 |  | 1 | 1 |  | 1 |  | 1 | 1 |  |  |  |  |  |  |  |  |
| Das, P. | 2015 | #1 | 1 | 1 | 1 | 1 | 1 |  | 1 |  | 1 | 1 | 1 | 1 |  |  |  |  |  |  |  |  |
| Dauncey, K | 1988 | #1 | 1 | 1 |  | 1 | 1 |  | 1 |  | 1 | 1 | 1 | 1 |  |  |  |  |  |  |  |  |
| Drake, M. E., Jr. | 1990 | #1 | 1 |  | 1 | 1 |  | 1 | 1 |  | 1 | 1 | 1 | 1 |  |  |  |  |  |  |  |  |
| Drake, M. E., Jr. | 1990 | #2 | 1 | 1 | 1 | 1 | 1 |  | 1 | 1 | 1 | 1 | 1 | 1 |  |  |  |  |  |  |  |  |
| El Hechmi, S. | 2013 | #1 | 1 |  |  |  |  |  | 1 |  |  |  |  |  |  |  |  |  |  |  |  | 1 |
| Estrade, J. F. | 1989 | #1 | 1 | 1 |  | 1 | 1 |  | 1 |  | 1 | 1 | 1 | 1 |  |  |  |  |  |  |  |  |
| Fawcett, R. G. | 1991 | #1 | 1 |  | 1 | 1 | 1 |  | 1 |  | 1 | 1 | 1 | 1 |  |  |  |  |  |  |  |  |
| Fenn, D. | 1999 | #1 | 1 |  | 1 | 1 | 1 | 1 | 1 |  | 1 |  | 1 | 1 |  |  |  |  |  |  |  |  |
| Filley, C. M. | 1995 | #6 |  |  |  | 1 | 1 |  | 1 |  |  |  |  |  |  |  |  |  |  |  |  | 1 |
| Gafoor, R. | 2003 | #1 | 1 | 1 | 1 | 1 | 1 |  | 1 | 1 |  |  |  | 1 |  |  |  |  |  |  |  | 1 |
| Gafoor, R. | 2003 | #2 | 1 | 1 | 1 |  |  |  | 1 | 1 | 1 | 1 | 1 | 1 |  |  |  |  |  |  |  |  |
| Gafoor, R. | 2003 | #3 | 1 |  | 1 |  |  |  | 1 |  |  |  |  |  |  |  |  |  |  |  |  | 1 |
| Gal, Paul | 1958 | #2 | 1 | 1 |  | 1 |  |  | 1 |  | 1 | 1 | 1 | 1 |  |  |  |  |  |  |  |  |
| Galindo Menendez, A. | 1996 | #2 | 1 | 1 | 1 |  |  | 1 | 1 | 1 | 1 | 0 | 1 | 1 |  |  |  |  |  |  |  |  |
| Garland, E. J. | 1991 | #2 | 1 | 1 | 1 | 1 | 1 |  | 1 | 1 | 1 | 1 | 1 | 1 |  |  |  |  |  |  |  |  |
| Goyal, R. | 2006 | #1 | 1 | 1 |  | 1 |  | 1 | 1 |  |  |  |  | 1 |  |  |  |  | 1 |  |  |  |
| Greenberg, D. B. | 1985 | #1 | 1 |  | 1 | 1 | 1 | 1 | 1 | 1 | 1 | 0 | 1 | 1 |  |  |  |  |  |  |  |  |
| Haq, M. Z. | 2009 | #1 | 1 | 1 | 1 | 1 |  | 1 | 1 |  | 1 | 1 | 1 | 1 |  |  |  |  |  |  |  |  |
| Heinrich, T. W. | 2004 | #1 | 1 |  |  | 1 | 1 |  | 1 |  | 1 | 0 | 1 | 1 |  |  |  |  |  |  |  |  |
| Huffman, J. | 2003 | #4 | 1 | 1 | 1 | 1 |  |  | 1 |  | 1 | 1 | 1 | 1 |  |  |  |  |  |  |  |  |
| Hunt, N. | 1990 | #1 | 1 | 1 |  |  |  |  | 1 |  |  |  |  |  |  |  |  |  |  |  |  | 1 |
| Inzelberg, R. | 2001 | #1 | 1 |  | 1 | 1 | 1 | 1 | 1 | 1 | 1 | 0 | 1 | 1 |  |  |  |  |  |  |  |  |
| Isles, L. J. | 1991 | #1 | 1 |  | 1 | 1 |  |  | 1 | 1 | 1 | 0 | 1 | 1 |  |  |  |  |  |  |  |  |
| Jagadesan, V. | 2014 | #1 | 1 | 1 | 1 | 1 |  | 1 | 1 |  | 1 |  | 1 | 1 |  |  |  |  |  |  |  |  |
| Jamieson, R. C. | 1979 | #1 | 1 | 1 | 1 | 1 |  | 1 | 1 | 1 | 1 | 1 | 1 | 1 |  |  |  |  |  |  |  |  |
| Jampala, V. C. | 1983 | #1 | 1 | 1 | 1 | 1 | 1 | 1 | 1 | 1 | 1 | 1 | 1 | 1 |  |  |  |  |  |  |  |  |
| Julayanont, P. | 2017 | #1 | 1 | 1 | 1 | 1 |  |  |  |  | 1 | 1 | 1 | 1 |  |  |  |  |  |  |  |  |
| Kanemoto, K | 1995 | #1 | 1 |  |  | 1 |  |  | 1 | 1 | 1 |  | 1 | 1 |  |  |  |  |  |  |  |  |
| Kar, S. K. | 2017 | #1 | 1 | 1 | 1 | 1 |  |  | 1 | 1 |  | 1 |  | 1 |  |  |  |  |  |  |  | 1 |
| Koreki, A. | 2012 | #1 | 1 |  | 1 | 1 | 1 |  | 1 |  |  |  |  | 1 | 1 |  |  |  |  |  |  |  |
| Kotrla, K. J. | 1994 | #1 | 1 | 1 | 1 | 1 | 1 |  | 1 |  | 1 | 1 | 1 | 1 |  |  |  |  |  |  |  |  |
| Ku, B. D. | 2006 | #1 | 1 |  | 1 | 1 |  | 1 | 1 |  | 1 | 1 | 1 | 1 |  |  |  |  |  |  |  |  |
| Kulisevsky, J. | 1993 | #1 | 1 | 1 | 1 | 1 | 1 | 1 | 1 |  | 1 | 1 | 1 | 1 |  |  |  |  |  |  |  |  |
| Kumar, S. K. | 1997 | #1 | 1 | 1 |  |  |  |  | 1 | 1 | 1 |  | 1 | 1 |  |  |  |  |  |  |  |  |
| Lauterbach, E. C. | 1996 | #3 | 1 | 1 | 1 |  | 1 | 1 | 1 | 1 |  |  |  | 1 |  |  | 1 | 1 |  |  |  |  |
| Lee, Y. M. | 2014 | #1 | 1 |  | 1 |  |  |  | 1 |  | 1 |  |  |  |  |  |  |  |  |  |  | 1 |
| Leibson, E. | 2000 | #1 | 1 | 1 |  | 1 |  | 1 | 1 |  |  |  |  | 1 |  |  |  | 1 |  |  |  |  |
| Liu, C. Y. | 1996 | #1 | 1 | 1 | 1 | 1 | 1 |  | 1 |  |  | 0 |  | 1 |  |  | 1 |  |  |  |  |  |
| Lupo, M. | 2018 | #1 | 1 |  |  |  |  |  | 1 | 1 | 1 | 1 |  |  |  | 1 |  |  |  |  |  |  |
| Malamud, Nathan | 1967 | #16 | 1 | 1 |  | 1 |  |  | 1 |  |  |  |  | 1 |  |  |  |  |  |  |  | 1 |
| Mark, Mordechi | 1991 | #1 |  |  |  |  |  |  |  |  |  |  |  |  |  |  |  |  |  |  |  | 1 |
| McKeown, S. P. | 1987 | #1 | 1 | 1 |  | 1 |  |  | 1 | 1 | 1 | 1 | 1 | 1 |  |  |  |  |  |  |  |  |
| Miller, B. L. | 1986 | #4 | 1 |  | 1 | 1 | 1 |  | 1 |  | 1 |  | 1 | 1 |  |  |  |  |  |  |  |  |
| Modrego, P. J. | 2000 | #2 | 1 |  | 1 | 1 | 1 |  | 1 |  | 1 | 1 | 1 | 1 |  |  |  |  |  |  |  |  |
| Mumoli, N. | 2013 | #1 | 1 |  |  |  |  | 1 | 1 | 1 |  |  |  | 1 |  |  |  |  |  |  |  | 1 |
| Murai, T. | 2003 | #1 | 1 |  | 1 | 1 |  | 1 | 1 |  |  |  |  | 1 |  | 1 |  |  |  |  |  |  |
| Mustafa, B. | 2005 | #1 | 1 |  | 1 | 1 |  | 1 | 1 |  | 1 |  | 1 | 1 |  |  |  |  |  |  |  |  |
| Nagaratnam, N | 1998 | #2 |  |  | 1 |  |  |  | 1 |  |  |  |  |  |  |  |  |  |  |  |  | 1 |
| Nagaratnam, N. | 2006 | #1 | 1 |  | 1 | 1 | 1 |  | 1 |  |  |  |  | 1 |  |  | 1 |  |  |  |  |  |
| Nagaratnam, N. | 2006 | #2 | 1 |  | 1 | 1 |  |  | 1 |  | 1 | 1 | 1 | 1 |  |  |  |  |  |  |  |  |
| Nizamie, S. H. | 1988 | #2 | 1 | 1 | 1 | 1 |  |  | 1 | 1 | 1 | 1 | 1 | 1 |  |  |  |  |  |  |  |  |
| OH, Koh | 2010 | #1 | 1 | 1 | 1 | 1 | 1 | 1 | 1 |  | 1 | 1 | 1 | 1 |  |  |  |  |  |  |  |  |
| Okun, Michael S | 2003 | #1 | 1 |  | 1 |  |  |  | 1 |  |  |  |  |  |  |  |  |  |  |  |  | 1 |
| Okun, Michael S | 2003 | #2 | 1 | 1 | 1 | 1 |  |  | 1 |  |  |  |  | 1 |  |  |  |  |  |  |  | 1 |
| Oppler, Willy | 1950 | #1 | 1 |  | 1 | 1 | 1 | 1 | 1 |  | 1 | 1 | 1 | 1 |  |  |  |  |  |  |  |  |
| Park, Soyeon | 2014 | #1 | 1 | 1 |  | 1 | 1 | 1 | 1 | 1 | 1 |  | 1 | 1 |  |  |  |  |  |  |  |  |
| Pathak, Abhishek | 2014 | #1 | 1 | 1 | 1 | 1 |  |  | 1 |  | 1 | 1 | 1 | 1 |  |  |  |  |  |  |  |  |
| Reisch, T. | 2005 | #1 | 1 |  | 1 |  |  |  | 1 | 1 |  |  |  | 1 |  |  |  |  |  |  |  | 1 |
| Robinson, R. G. | 1984 | Group |  |  |  |  |  |  |  |  |  |  |  |  |  |  |  |  |  |  | 1 |  |
| Robinson, R. G. | 1988 | Group | 1 | 1 | 1 | 1 | 1 | 1 | 1 |  | 1 | 1 | 1 | 1 |  |  |  |  |  |  |  |  |
| Rocha, F. F. | 2008a | #1 | 1 | 1 | 1 | 1 | 1 | 1 | 1 |  | 1 | 1 | 1 | 1 |  |  |  |  |  |  |  |  |
| Rocha, F. F. | 2008b | #1 | 1 |  | 1 | 1 | 1 |  |  |  | 1 |  | 1 | 1 |  |  |  |  |  |  |  |  |
| Rosenbaum, A. H. | 1975 | #1 | 1 | 1 |  | 1 |  |  | 1 |  | 1 | 1 | 1 | 1 |  |  |  |  |  |  |  |  |
| Routh, Rajdeep | 2014 | #1 | 1 |  | 1 | 1 | 1 | 1 | 1 | 1 | 1 | 1 | 1 | 1 |  |  |  |  |  |  |  |  |
| Salazar-Calderon Perriggo, V. H. | 1993 | #1 | 1 | 1 | 1 | 1 |  |  | 1 |  | 1 | 1 | 1 | 1 |  |  |  |  |  |  |  |  |
| Sanders, Richard D | 1994 | #1 |  |  | 1 | 1 |  | 1 |  |  | 1 | 0 |  |  |  |  |  |  |  |  |  | 1 |
| Semiz, M. | 2010 | #1 | 1 | 1 | 1 |  |  |  | 1 |  | 1 |  | 1 | 1 |  |  |  |  |  |  |  |  |
| Semiz, U. B. | 2005 | #1 |  | 1 | 1 |  | 1 | 1 | 1 |  |  |  |  |  |  |  |  |  |  |  |  | 1 |
| Sidhom, Youssef | 2014 | #3 | 1 | 1 | 1 |  |  |  | 1 |  | 1 |  | 1 | 1 |  |  |  |  |  |  |  |  |
| Starkstein, S. E. | 1987 | Group | 1 | 1 | 1 | 1 | 1 | 1 | 1 |  | 1 | 1 | 1 | 1 |  |  |  |  |  |  |  |  |
| Starkstein, S. E. | 1988 | #1 | 1 | 1 | 1 | 1 |  |  | 1 |  | 1 | 1 | 1 | 1 |  |  |  |  |  |  |  |  |
| Starkstein, S. E. | 1988 | #2 | 1 |  | 1 | 1 | 1 |  | 1 |  | 1 |  | 1 | 1 |  |  |  |  |  |  |  |  |
| Starkstein, S. E. | 1988 | #4 | 1 | 1 | 1 | 1 | 1 |  | 1 |  | 1 | 1 | 1 | 1 |  |  |  |  |  |  |  |  |
| Starkstein, S. E. | 1988 | #5 |  | 1 | 1 |  |  |  | 1 | 1 | 1 | 1 |  |  |  |  |  |  |  |  |  | 1 |
| Starkstein, S. E. | 1988 | #6 | 1 |  | 1 | 1 |  |  | 1 |  |  |  |  | 1 |  |  |  | 1 |  |  |  |  |
| Starkstein, S. E. | 1988 | #7 | 1 |  |  | 1 | 1 |  | 1 | 1 | 1 |  | 1 | 1 |  |  |  |  |  |  |  |  |
| Starkstein, S. E. | 1988 | #8 | 1 | 1 | 1 | 1 | 1 |  | 1 |  | 1 | 1 | 1 | 1 |  |  |  |  |  |  |  |  |
| Starkstein, S. E. | 1988 | #9 | 1 | 1 |  | 1 | 1 |  | 1 |  | 1 | 1 | 1 | 1 |  |  |  |  |  |  |  |  |
| Starkstein, S. E. | 1988 | #10 | 1 |  | 1 | 1 | 1 |  | 1 | 1 | 1 | 1 | 1 | 1 |  |  |  |  |  |  |  |  |
| Starkstein, S. E. | 1990 | #1 | 1 | 1 | 1 | 1 | 1 |  | 1 | 1 | 1 | 1 | 1 | 1 |  |  |  |  |  |  |  |  |
| Starkstein, S. E. | 1990 | #2 | 1 | 1 | 1 | 1 |  | 1 |  |  | 1 | 1 | 1 | 1 |  |  |  |  |  |  |  |  |
| Starkstein, S. E. | 1990 | #3 | 1 | 1 | 1 | 1 | 1 |  | 1 |  | 1 | 1 | 1 | 1 |  |  |  |  |  |  |  |  |
| Starkstein, S. E. | 1990 | #4 | 1 | 1 | 1 | 1 | 1 |  | 1 | 1 | 1 | 1 | 1 | 1 |  |  |  |  |  |  |  |  |
| Starkstein, S. E. | 1990 | #5 | 1 | 1 | 1 | 1 | 1 |  | 1 | 1 | 1 | 1 | 1 | 1 |  |  |  |  |  |  |  |  |
| Starkstein, S. E. | 1990 | #6 | 1 | 1 | 1 | 1 | 1 |  | 1 | 1 | 1 | 1 | 1 | 1 |  |  |  |  |  |  |  |  |
| Starkstein, S. E. | 1990 | #7 | 1 | 1 | 1 | 1 | 1 |  | 1 | 1 | 1 | 1 | 1 | 1 |  |  |  |  |  |  |  |  |
| Starkstein, S. E. | 1990 | #8 | 1 | 1 |  | 1 | 1 |  | 1 | 1 | 1 | 1 | 1 | 1 |  |  |  |  |  |  |  |  |
| Starkstein, S. E. | 1991 | Group | 1 | 1 | 1 | 1 | 1 |  | 1 | 1 | 1 | 1 | 1 | 1 |  |  |  |  |  |  |  |  |
| Stern, K | 1942 | #1 | 1 |  | 1 | 1 | 1 |  | 1 |  | 1 |  | 1 | 1 |  |  |  |  |  |  |  |  |
| Sullivan, G. | 1995 | #1 | 1 | 1 |  | 1 | 1 |  | 1 |  | 1 | 1 | 1 | 1 |  |  |  |  |  |  |  |  |
| Sweet, Robert A | 1990 | #1 | 1 | 1 |  | 1 | 1 |  | 1 |  | 1 | 1 | 1 | 1 |  |  |  |  |  |  |  |  |
| Taylor, J. B. | 2018 | #1 | 1 | 1 | 1 | 1 | 1 |  | 1 | 1 | 1 |  | 1 | 1 |  |  |  |  |  |  |  |  |
| Topcuoglu, Volkan | 2012 | #1 | 1 |  | 1 | 1 |  | 1 | 1 | 1 | 1 | 1 | 1 | 1 |  |  |  |  |  |  |  |  |
| Trillet, M. | 1995 | #1 | 1 |  | 1 | 1 | 0 |  | 1 |  | 0 | 0 |  | 1 |  |  |  |  |  |  |  | 1 |
| Trimble, Michael R | 1981 | #2 | 1 |  |  |  |  | 1 | 1 |  | 1 | 1 |  |  |  |  |  |  |  |  |  | 1 |
| Turecki, G. | 1993 | #1 | 1 | 1 | 1 | 1 |  |  | 1 | 1 | 1 | 1 | 1 | 1 |  |  |  |  |  |  |  |  |
| Vidrih, B. | 2012 | #1 | 1 | 1 | 1 | 1 | 1 |  | 1 |  | 1 | 1 | 1 | 1 |  |  |  |  |  |  |  |  |
| Wright, M. T. | 1997 | #1 | 1 |  | 1 | 1 |  |  | 1 |  | 1 |  | 1 | 1 |  |  |  |  |  |  |  |  |
| Ybarra, Mariana Ines | 2007 | #2 | 1 |  |  | 1 |  |  | 1 |  |  | 0 |  |  | 1 |  |  |  |  |  |  |  |
| Ybarra, Mariana Ines | 2007 | #3 | 1 |  | 1 | 1 |  |  | 1 |  |  |  |  | 1 | 1 |  |  |  |  |  |  |  |
| Yetimalar, Y. | 2007 | #1 | 1 | 1 | 1 | 1 |  | 1 | 1 |  | 1 |  | 1 | 1 |  |  |  |  |  |  |  |  |
| Zincir, S. B. | 2014 | #1 | 1 |  | 1 | 1 | 1 |  | 1 | 1 | 1 |  | 1 | 1 |  |  |  |  |  |  |  |  |

Crit. – Criteria; DSM – Diagnostic and Statistical Manual of Mental Disorders; ICD – International Classification of Diseases; MAS – Mania Acute Scale; Psc. – Psychotic Symptoms; PSE – Present State Exam; Uns. – Unspecified

0 = absence; 1 = presence; blank = not defined/unknown

^a^ Criterion C (marked impairment in social or occupational functioning, need for hospitalization or presence of psychotic symptoms) was only considered to be present when authors explicitly reported marked impairment in social or occupational functioning, hospitalization due to the psychiatric syndrome, or when psychotic symptoms were described or otherwise reported as present.

^b^ Full DSM 5 Criteria, i.e. (A + ≥ 3 B’s + C)

^c^ Core clinical DSM 5 criteria, i.e. (A + ≥ 3 B’s)

This table was completed using three priority criteria levels. First, we prioritized classification according to DSM 5 criteria. If not enough DSM5 criteria were reported, we extracted which criteria/Classification System was used by the authors to classify the clinical case. If no criteria/Classification System was reported by the authors, but it was unequivocally stated that the clinical case qualified for a manic syndrome diagnosis, we registered the diagnosis as based on Unspecified Criteria.

**Table S5** – Subgroup analyses of lesional mania cases according to gender, age, hand dominance, time-lapse between brain lesion and mania onset, affective episode recurrence and lesion etiology.

| **Subgroup** | **Side** | **Gender ^a^** | | | **Age ^a^** | | | **Hand Dominance ^a^** | | | **Time E-MM ^a^** | | | **Affective Episode Recurrence ^a^** | | | **Etiology ^a^** | | |
| --- | --- | --- | --- | --- | --- | --- | --- | --- | --- | --- | --- | --- | --- | --- | --- | --- | --- | --- | --- |
|  |  | **N (%)** | | **p*** | **N (%)** | | **p*** | **N (%)** | | **p*** | **N (%)** | | **p*** | **N (%)** | | **p*** | **N (%)** | | **p*** |
|  |  | **Female (N=45)** | **Male (N=87)** |  | **≤52y b (N=62)** | **>52y (N=62)** |  | **Right (N=40)** | **Non-Right (N=5)** |  | **≤1m (N=38)** | **>1m (N=34)** |  | **With Recurrence (N=35)** | **Without Recurrence (N=41)** |  | **Vascular (N=77)** | **Non-Vascular (N=70)** |  |
| **Total** | **Right** | 41 (91.1) | 69 (79.3) | n.s. | 44 (77.19) | 48 (84.21) | n.s. | 30 (75.0) | 3 (60.0) | n.s. | 32 (84.2) | 21 (72.4) | n.s. | 27 (79.41) | 30 (83.33) | n.s. | 63 (81.8) | 52 (86.7) | n.s. |
|  | **Left** | 21 (46.7) | 37 (42.5) | n.s. | 29 (50.88) | 24 (42.11) | n.s. | 16 (40.0) | 1 (20.0) | n.s. | 14 (36.8) | 15 (51.7) | n.s. | 13 (38.24) | 12 (33.33) | n.s. | 27 (35.1) | 30 (50.0) | n.s. |
| **Frontal** | **Right** | 13 (28.9) | 19 (21.8) | n.s. | 18 (31.58) | 10 (17.54) | n.s. | 7 (17.5) | 1 (20.0) | n.s. | 7 (18.4) | 4 (13.8) | n.s. | 9 (26.47) | 7 (19.44) | n.s. | 10 (13.0) | 21 (35.0) | 0.01 |
|  | **Left** | 5 (11.1) | 16 (18.4) | n.s. | 10 (17.54) | 10 (17.54) | n.s. | 6 (15.0) | 1 (20.0) | n.s. | 5 (13.2) | 5 (17.2) | n.s. | 8 (23.53) | 4 (11.11) | n.s. | 5 (6.5) | 15 (25.0) | 0.01 |
| **Temporal** | **Right** | 10 (22.2) | 27 (31.0) | n.s. | 14 (24.56) | 15 (26.32) | n.s. | 9 (22.5) | 2 (40.0) | n.s. | 8 (21.1) | 9 (31.0) | n.s. | 2 (5.88) | 10 (27.78) | n.s. | 16 (20.8) | 20 (33.3) | n.s. |
|  | **Left** | 4 (8.9) | 10 (11.5) | n.s. | 9 (15.79) | 5 (8.77) | n.s. | 3 (7.5) | 0 (0.0) | n.s. | 2 (5.3) | 6 (20.7) | n.s. | 4 (11.76) | 3 (8.33) | n.s. | 4 (5.2) | 9 (15.0) | n.s. |
| **Parietal** | **Right** | 4 (8.9) | 15 (17.2) | n.s. | 7 (12.28) | 11 (19.30) | n.s. | 4 (10.0) | 0 (0.0) | n.s. | 7 (18.4) | 5 (17.2) | n.s. | 4 (11.76) | 9 (25.00) | n.s. | 11 (14.3) | 8 (13.3) | n.s. |
|  | **Left** | 1 (2.2) | 4 (4.6) | n.s. | 1 (1.75) | 4 (7.02) | n.s. | 3 (7.5) | 1 (20.0) | n.s. | 0 (0.0) | 3 (10.3) | n.s. | 2 (5.88) | 1 (2.78) | n.s. | 5 (6.5) | 0 (0.0) | n.s. |
| **Occipital** | **Right** | 3 (6.7) | 4 (4.6) | n.s. | 2 (3.51) | 4 (7.02) | n.s. | 3 (7.5) | 0 (0.0) | n.s. | 1 (2.6) | 3 (10.3) | n.s. | 2 (5.88) | 2 (5.56) | n.s. | 2 (2.6) | 5 (8.3) | n.s. |
|  | **Left** | 1 (2.2) | 0 (0.0) | n.s. | 1 (1.75) | 0 (0.00) | n.s. | 0 (0.0) | 0 (0.0) | n.s. | 0 (0.0) | 1 (3.5) | n.s. | 1 (2.94) | 0 (0.00) | n.s. | 0 (0.0) | 1 (1.7) | n.s. |
| **Insula** | **Right** | 1 (2.2) | 3 (3.45) | n.s. | 2 (3.51) | 2 (3.51) | n.s. | 1 (2.5) | 0 (0.0) | n.s. | 0 (0.0) | 3 (10.3) | n.s. | 1 (2.94) | 1 (2.78) | n.s. | 3 (3.9) | 1 (1.7) | n.s. |
|  | **Left** | 1 (2.2) | 3 (3.45) | n.s. | 1 (1.75) | 3 (5.26) | n.s. | 2 (5.0) | 0 (0.0) | n.s. | 1 (2.6) | 1 (3.5) | n.s. | 1 (2.94) | 1 (2.78) | n.s. | 3 (3.9) | 1 (1.7) | n.s. |
| **Basal Ganglia** | **Right** | 12 (26.7) | 11 (12.6) | n.s. | 4 (7.02) | 10 (17.54) | n.s. | 5 (12.5) | 0 (0.0) | n.s. | 4 (10.5) | 4 (13.8) | n.s. | 3 (8.82) | 3 (8.33) | n.s. | 20 (25.97) | 3 (5.0) | 0.0002* |
|  | **Left** | 6 (13.3) | 6 (6.9) | n.s. | 4 (7.02) | 7 (12.28) | n.s. | 5 (12.5) | 0 (0.0) | n.s. | 4 (10.5) | 2 (6.9) | n.s. | 0 (0.0) | 2 (5.56) | n.s. | 8 (10.39) | 4 (6.7) | n.s. |
| **Thalamus** | **Right** | 10 (22.2) | 14 (16.1) | n.s. | 9 (15.79) | 13 (22.81) | n.s. | 7 (17.5) | 1 (20.0) | n.s. | 8 (21.1) | 3 (10.3) | n.s. | 1 (2.94) | 10 (27.78) | n.s. | 17 (22.1) | 7 (11.7) | n.s. |
|  | **Left** | 2 (4.4) | 6 (6.9) | n.s. | 5 (8.77) | 3 (5.26) | n.s. | 1 (2.5) | 0 (0.0) | n.s. | 1 (2.6) | 1 (3.5) | n.s. | 0 (0.0) | 3 (8.33) | n.s. | 4 (5.2) | 4 (6.7) | n.s. |
| **Hypothalamus^d^** | **Right** | 2 (4.4) | 1 (1.2) | n.s. | 2 (3.51) | 1 (1.75) | n.s. | 1 (2.5) | 0 (0.0) | n.s. | 1 (2.6) | 1 (3.5) | n.s. | 0 (0.0) | 1 (2.78) | n.s. | 1 (1.3) | 2 (3.3) | n.s. |
|  | **Left** | 1 (2.2) | 0 (0.0) | n.s. | 0 (0.00) | 1 (1.75) | n.s. | 1 (2.5) | 0 (0.0) | n.s. | 0 (0.0) | 0 (0.0) | n.s. | 0 (0.0) | 0 (0.00) | n.s. | 0 (0.0) | 1 (1.7) | n.s. |
| **White Matter** | **Right** | 12 (26.7) | 11 (12.6) | n.s. | 7 (12.28) | 10 (17.54) | n.s. | 3 (7.5) | 0 (0.0) | n.s. | 2 (5.3) | 2 (6.9) | n.s. | 5 (14.71) | 2 (5.56) | n.s. | 16 (20.8) | 7 (11.7) | n.s. |
|  | **Left** | 7 (15.56) | 9 (10.3) | n.s. | 6 (10.53) | 8 (14.04) | n.s. | 3 (7.5) | 0 (0.0) | n.s. | 1 (2.6) | 2 (6.9) | n.s. | 1 (2.94) | 3 (8.33) | n.s. | 10 (13.0) | 6 (10.0) | n.s. |
| **Cerebellum** | **Right** | 2 (4.4) | 4 (4.6) | n.s. | 4 (7.02) | 1 (1.75) | n.s. | 0 (0.0) | 0 (0.0) | n.s. | 1 (2.6) | 0 (0.0) | n.s. | 3 (8.82) | 1 (2.78) | n.s. | 1 (1.3) | 5 (8.3) | n.s. |
|  | **Left** | 3 (6.7) | 2 (2.3) | n.s. | 4 (7.02) | 1 (1.75) | n.s. | 1 (2.5) | 0 (0.0) | n.s. | 0 (0.0) | 1 (3.5) | n.s. | 2 (5.88) | 0 (0.00) | n.s. | 3 (3.9) | 2 (3.3) | n.s. |
| **Brainstem** | **Right** | 3 (6.7) | 8 (9.2) | n.s. | 4 (7.02) | 5 (8.77) | n.s. | 1 (2.5) | 1 (20.0) | n.s. | 2 (5.3) | 0 (0.0) | n.s. | 6 (17.65) | 3 (8.33) | n.s. | 6 (7.8) | 5 (8.3) | n.s. |
|  | **Left** | 3 (6.7) | 5 (5.8) | n.s. | 5 (8.77) | 3 (5.26) | n.s. | 1 (2.5) | 0 (0.0) | n.s. | 0 (0.0) | 1 (3.5) | n.s. | 2 (5.88) | 3 (8.33) | n.s. | 5 (6.5) | 3 (5.0) | n.s. |
| **Other Brain** | **Right** | 2 (4.4) | 1 (1.2) | n.s. | 0 (0.00) | 1 (1.75) | n.s. | 1 (2.5) | 0 (0.0) | n.s. | 2 (5.3) | 0 (0.0) | n.s. | 0 (0.0) | 0 (0.0) | n.s. | 2 (2.6) | 1 (1.7) | n.s. |
|  | **Left** | 2 (4.4) | 1 (1.2) | n.s. | 0 (0.00) | 1 (1.75) | n.s. | 1 (2.5) | 0 (0.0) | n.s. | 2 (5.3) | 0 (0.0) | n.s. | 0 (0.0) | 0 (0.0) | n.s. | 2 (2.6) | 1 (1.7) | n.s. |
| **Unspecified** | **Right** | 0 (0.0) | 0 (0.0) | n.s. | 0 (0.00) | 0 (0.00) | n.s. | 6 (15.0) | 0 (0.0) | n.s. | 0 (0.0) | 0 (0.0) | n.s. | 0 (0.0) | 0 (0.0) | n.s. | 6 (7.8) | 0 (0.0) | 0.03 |
|  | **Left** | 0 (0.0) | 0 (0.0) | n.s. | 0 (0.00) | 0 (0.00) | n.s. | 0 (0.0) | 0 (0.0) | n.s. | 0 (0.0) | 0 (0.0) | n.s. | 0 (0.0) | 0 (0.0) | n.s. | 0 (0.0) | 0 (0.0) | n.s. |

**E-MM** – time between event causing brain insult and the manic/mixed state episode onset; **m** – month; **y** – years-old

^a^ Does not include all case reports since not all articles reported this information

^b^ p-value for Fisher’s exact tests comparing lesion frequency between groups

^c^ When age was considered, subgroups were defined according to sample’s median: 52 years-old

^d^ White matter includes corona radiata, semi-ovale center, internal capsule, external capsule, corpus callosum and unspecified white matter regions.

* P-values that remain significant after correcting for multiple comparisons. Statistical significance was defined using a False Discovery Rate (FDR) of 0.01, according to Benjamini-Hochberg (123)

**Table S6** – Subgroup analyses of lesional mania cases according to availability of lesion scan and clinical quality assessment (CQA).

| **Subgroup** | **Side** | **Neuroimage Report ^a^** | | | **Clinical Quality Assessment ^a^** | | |
| --- | --- | --- | --- | --- | --- | --- | --- |
|  |  | **N (%)** | | **p^b^** | **N (%)** | | **p^b^** |
|  |  | **With Neuroimage (N=118)** | **Without Neuroimage (N=93)** |  | **≤3 (N=53)** | **>3 (N=158)** |  |
| **Total** | **Right** | 97 (81.51) | 72 (78.26) | n.s. | 47 (92.16) | 122 (81.33) | n.s. |
|  | **Left** | 42 (35.29) | 29 (31.52) | n.s. | 19 (37.25) | 52 (34.67) | n.s. |
| **Frontal** | **Right** | 27 (22.69) | 15 (16.30) | n.s. | 12 (23.53) | 30 (20.00) | n.s. |
|  | **Left** | 15 (12.61) | 12 (13.04) | n.s. | 5 (9.80) | 22 (14.67) | n.s. |
| **Temporal** | **Right** | 31 (26.05) | 30 (32.61) | n.s. | 14 (27.45) | 47 (31.33) | n.s. |
|  | **Left** | 6 (5.04) | 12 (13.04) | 0.05 | 5 (9.80) | 13 (8.67) | n.s. |
| **Parietal** | **Right** | 8 (6.72) | 12 (13.04) | n.s. | 8 (15.69) | 12 (8.00) | n.s. |
|  | **Left** | 9 (7.56) | 3 (3.26) | n.s. | 0 (0.00) | 5 (3.33) | n.s. |
| **Occipital** | **Right** | 5 (4.20) | 3 (3.26) | n.s. | 0 (0.00) | 8 (5.33) | n.s. |
|  | **Left** | 0 (0.00) | 1 (1.09) | n.s. | 0 (0.00) | 1 (0.67) | n.s. |
| **Insula** | **Right** | 2 (1.68) | 2 (2.17) | n.s. | 2 (3.92) | 2 (1.33) | n.s. |
|  | **Left** | 2 (1.68) | 2 (2.17) | n.s. | 0 (0.00) | 4 (2.67) | n.s. |
| **Basal Ganglia** | **Right** | 26 (21.85) | 4 (4.35) | 0.0002* | 11 (21.57) | 19 (12.67) | n.s. |
|  | **Left** | 8 (6.72) | 4 (4.35) | n.s. | 4 (7.84) | 8 (5.33) | n.s. |
| **Thalamus** | **Right** | 22 (18.49) | 8 (8.70) | 0.05 | 12 (23.53) | 18 (12.00) | n.s. |
|  | **Left** | 5 (4.20) | 3 (3.26) | n.s. | 4 (7.84) | 4 (2.67) | n.s. |
| **Hypothalamus** | **Right** | 3 (2.52) | 0 (0.00) | n.s. | 2 (3.92) | 1 (0.67) | n.s. |
|  | **Left** | 1 (0.84) | 0 (0.00) | n.s. | 1 (1.96) | 0 (0.00) | n.s. |
| **White Matter ^c^** | **Right** | 21 (17.65) | 4 (4.35) | 0.002* | 14 (27.45) | 11 (7.33) | n.s. |
|  | **Left** | 11 (9.24) | 5 (5.43) | n.s. | 9 (17.65) | 7 (4.67) | n.s. |
| **Cerebellum** | **Right** | 3 (2.52) | 3 (3.26) | n.s. | 4 (7.84) | 2 (1.33) | n.s. |
|  | **Left** | 2 (1.68) | 3 (3.26) | n.s. | 4 (7.84) | 1 (0.67) | n.s. |
| **Brainstem** | **Right** | 2 (1.68) | 9 (9.78) | 0.01* | 6 (11.76) | 5 (3.33) | n.s. |
|  | **Left** | 3 (2.52) | 5 (5.43) | n.s. | 5 (9.80) | 3 (2.00) | n.s. |
| **Other Brain** | **Right** | 1 (0.84) | 2 (2.17) | n.s. | 1 (1.96) | 2 (1.33) | n.s. |
|  | **Left** | 1 (0.84) | 2 (2.17) | n.s. | 1 (1.96) | 2 (1.33) | n.s. |
| **Unspecified** | **Right** | 10 (8.40) | 6 (6.52) | n.s. | 6 (11.76) | 10 (6.67) | n.s. |
|  | **Left** | 3 (2.52) | 0 (0.00) | n.s. | 0 (0.00) | 3 (2.00) | n.s. |

^a^ Does not include all case reports since not all articles reported this information

^b^ p-value for Fisher’s exact tests comparing lesion frequency between groups

^c^ White matter includes corona radiata, semi-ovale center, internal capsule, external capsule, corpus callosum and unspecified white matter regions.

* P-values that remain significant after correcting for multiple comparisons. Statistical significance was defined using a False Discovery Rate (FDR) of 0.01, according to Benjamini-Hochberg (123)

**Table S7** – Cerebellum and White Matter Analyses of Lesions with Available Images: Comparisons of Right vs. Left Hemisphere Lesions and of Right Hemisphere Stroke Lesions with a Control Sample of Stroke

| **Area** | **% lesioned voxels^a^ (mean ± SD; N=56)** | | | | | **% lesioned voxels^a^ (mean ± SD)** | | | | |
| --- | --- | --- | --- | --- | --- | --- | --- | --- | --- | --- |
|  | **Left** | | **Right** | | **p  (Sign test)^b^** | **Sperber et al (N=439)** | | **Barahona-Corrêa et al (N=29)** | | **p (Rank-sum test)^c^** |
|  | **Mean** | **SD** | **Mean** | **SD** |  | **Mean** | **SD** | **Mean** | **SD** |  |
| **GREY MATTER** | | | | | | | | | | |
| **CEREBELLUM** | | | | | | | | | | |
| Crus I of cerebellar hemisphere | 0 | 0 | 0.05 | 0.27 | n.s. |  |  |  |  |  |
| Crus II of cerebellar hemisphere | 0 | 0 | 0 | 0 | NA |  |  |  |  |  |
| Lobule III of cerebellar hemisphere | 0 | 0 | 0.02 | 0.09 | n.s. |  |  |  |  |  |
| Lobule IV, V of cerebellar hemisphere | 0.04 | 0.26 | 0.13 | 0.50 | n.s. |  |  |  |  |  |
| Lobule VI of cerebellar hemisphere | 0.03 | 0.21 | 0.10 | 0.44 | n.s. |  |  |  |  |  |
| Lobule VIIB of cerebellar hemisphere | 0 | 0 | 0.01 | 0.09 | n.s. |  |  |  |  |  |
| Lobule VIII of cerebellar hemisphere | 0 | 0 | 0.05 | 0.36 | n.s. |  |  |  |  |  |
| Lobule IX of cerebellar hemisphere | 0 | 0 | 0.02 | 0.14 | n.s. |  |  |  |  |  |
| Lobule X of cerebellar hemisphere | 0 | 0 | 0 | 0 | NA |  |  |  |  |  |
| **WHITE MATTER** | | | | | | | | | | |
| Corticospinal tract | 0.02 | 0.12 | 0 | 0 | n.s. | 0 | 0 | 0 | 0 | N.A. |
| Medial lemniscus | 0.003 | 0.02 | 0 | 0 | n.s. | 0 | 0 | 0 | 0 | N.A. |
| Inferior cerebellar peduncle | 0.03 | 0.19 | 0 | 0 | n.s. | 0 | 0 | 0 | 0 | N.A. |
| Superior cerebellar peduncle | 0 | 0 | 0.004 | 0.03 | n.s. | 0.09 | 0.87 | 0.01 | 0.04 | n.s. |
| Cerebral peduncle | 0.02 | 0.13 | 0 | 0 | n.s. | 1.95 | 4.10 | 0 | 0 | 0.0006 |
| Anterior limb of internal capsule | 0.05 | 0.31 | 0.39 | 1.25 | n.s. | 1.60 | 3.29 | 0.70 | 1.68 | n.s. |
| Posterior limb of internal capsule | 0.03 | 0.21 | 0.65 | 1.65 | 0.0001 | 0.87 | 1.77 | 1.03 | 2.10 | n.s. |
| Retrolenticular part of internal capsule | 0.02 | 0.07 | 0.43 | 1.24 | n.s. | 2.14 | 5.06 | 0.73 | 1.61 | n.s. |
| Anterior corona radiata | 0.25 | 0.84 | 0.43 | 1.32 | n.s. | 0.98 | 2.24 | 0.45 | 1.27 | n.s. |
| Superior corona radiata | 0.02 | 0.09 | 0.12 | 0.60 | n.s. | 0.35 | 0.86 | 0.16 | 0.78 | 0.02 |
| Posterior corona radiata | 0.01 | 0.05 | 0.16 | 0.69 | n.s. | 0.86 | 2.01 | 0.25 | 0.92 | 0.03 |
| Posterior thalamic radiation | 0.02 | 0.10 | 0.33 | 0.90 | n.s. | 0.30 | 1.09 | 0.45 | 1.02 | n.s. |
| Sagittal stratum | 0.04 | 0.19 | 0.89 | 2.97 | n.s. | 3.92 | 6.72 | 1.24 | 3.19 | n.s. |
| External capsule | 0.08 | 0.29 | 0.29 | 1.21 | n.s. | 0.02 | 0.14 | 0.54 | 1.65 | 6.4x10^-18^ |
| Cingulum (cingulate gyrus) | 0.02 | 0.16 | 0.03 | 0.16 | n.s. | 0.07 | 0.29 | 0 | 0 | n.s. |
| Cingulum (hippocampus) | 0.01 | 0.07 | 0.65 | 2.38 | n.s. | 0.35 | 1.28 | 0.85 | 2.52 | n.s. |
| Fornix (cres) / Stria terminalis | 0.03 | 0.17 | 0.43 | 1.83 | n.s. | 6.30 | 13.04 | 0.43 | 1.33 | 0.005 |
| Superior longitudinal fasciculus | 0.02 | 0.12 | 0.13 | 0.43 | n.s. | 0.11 | 0.35 | 0.20 | 0.52 | n.s. |
| Superior fronto-occipital fasciculus | 0.01 | 0.08 | 0.37 | 2.09 | n.s. | 0.17 | 1.17 | 0.19 | 0.78 | n.s. |
| Uncinate fasciculus | 0.13 | 0.92 | 0.28 | 1.36 | n.s. | 1.03 | 2.91 | 0.44 | 1.81 | n.s. |
| Tapetum | 0 | 0 | 0.26 | 1.03 | n.s. | 0 | 0 | 0.50 | 1.40 | 5.9x10^-15^ |

^a^ Displayed values are means and standard deviations. Please see Supplementary Table S8 for medians, minimums and maximums.

^b^ p-value for Sign tests comparing left- and right-hemisphere lesion volumes based on quantitative GM and WM analysis. The values being compared reflect the proportion of voxels in each AAL and JHU atlas area that are included in the lesion. Statistical significance was defined using a False Discovery Rate (FDR) of 0,01, according to Benjamini-Hochberg (123).

^c^ p-value for Wilcoxon rank-sum tests comparing lesion volumes based on quantitative GM and WM analysis between vascular lesional mania cases and an unselected sample of right-sided stroke described by Sperber & Karnath (124). The values being compared reflect the median proportion of voxels in each AAL and JHU atlas area that are included in the lesion.

Statistical significance was defined using a False Discovery Rate (FDR) of 0,01, according to Benjamini-Hochberg (123).

NA – Not Applicable; n.s. – non-significant; SD – Standard Deviation;

**Table S8** – Median, minimum and maximum from the comparison of Right vs. Left Hemisphere Lesions and of Right Hemisphere Stroke Lesions with a Control Sample of Stroke

| **Area** | **% lesioned voxels (N=56)** | | | | | | | **% lesioned voxels** | | | | | | |
| --- | --- | --- | --- | --- | --- | --- | --- | --- | --- | --- | --- | --- | --- | --- |
|  | **Left** | | | **Right** | | | **p  (Sign Test)^a^** | **Sperber et al (N=439)** | | | **Barahona-Corrêa et al (N=29)** | | | **p (Rank-sum test)^b^** |
|  | **Median** | **Min** | **Max** | **Median** | **Min** | **Max** |  | **Median** | **Min** | **Max** | **Median** | **Min** | **Max** |  |
| **GREY MATTER** | | | | | | | | | | | | | | |
| **FRONTAL** | | | | | | | | | | | | | | |
| Precentral gyrus | 0 | 0 | 0.33 | 0 | 0 | 0.40 | n.s. | 0 | 0 | 7.10 | 0 | 0 | 0.18 | 0.009 |
| Superior frontal gyrus | 0 | 0 | 2.45 | 0 | 0 | 3.39 | n.s. | 0 | 0 | 3.19 | 0 | 0 | 0.79 | n.s. |
| Superior frontal gyrus, orbital part | 0 | 0 | 9.16 | 0 | 0 | 9.23 | n.s. | 0 | 0 | 3.02 | 0 | 0 | 9.23 | 0.005 |
| Middle frontal gyrus | 0 | 0 | 2.36 | 0 | 0 | 3.64 | n.s. | 0 | 0 | 5.17 | 0 | 0 | 1.82 | n.s. |
| Middle frontal gyrus, orbital part | 0 | 0 | 9.81 | 0 | 0 | 9.53 | n.s. | 0 | 0 | 3.79 | 0 | 0 | 9.53 | n.s. |
| Inferior frontal gyrus, pars opercularis | 0 | 0 | 1.77 | 0 | 0 | 3.86 | n.s. | 0 | 0 | 11.71 | 0 | 0 | 3.86 | 0.0007 |
| Inferior frontal gyrus, pars triangularis | 0 | 0 | 4.08 | 0 | 0 | 5.85 | n.s. | 0 | 0 | 11.67 | 0 | 0 | 5.85 | 0.004 |
| Inferior frontal gyrus, pars orbitalis | 0 | 0 | 10.38 | 0 | 0 | 9.67 | n.s. | 0 | 0 | 8.74 | 0 | 0 | 6.70 | n.s. |
| Rolandic opercullum | 0 | 0 | 5.21 | 0 | 0 | 6.14 | n.s. | 0 | 0 | 12.81 | 0 | 0 | 6.14 | 0.001 |
| Supplmentar motor area | 0 | 0 | 0 | 0 | 0 | 0.49 | n.s. | 0 | 0 | 8.83 | 0 | 0 | 0.00 | n.s. |
| Olfactory cortex | 0 | 0 | 10.61 | 0 | 0 | 12.99 | n.s. | 0 | 0 | 3.54 | 0 | 0 | 5.38 | n.s. |
| Medial frontal gyrus | 0 | 0 | 3.10 | 0 | 0 | 3.73 | n.s. | 0 | 0 | 5.02 | 0 | 0 | 2.08 | n.s. |
| Medial orbitofrontal cortex | 0 | 0 | 14.69 | 0 | 0 | 13.70 | n.s. | 0 | 0 | 5.66 | 0 | 0 | 13.70 | 0.0006 |
| Rectus gyrus | 0 | 0 | 17.80 | 0 | 0 | 18.06 | n.s. | 0 | 0 | 1.38 | 0 | 0 | 8.74 | 0.005 |
| **TEMPORAL AND INSULA** | | | | | | | | | | | | | | |
| Hippocampus | 0 | 0 | 0.46 | 0 | 0 | 7.88 | 0.00002 | 0 | 0 | 7.47 | 0.09 | 0 | 5.76 | 9.3x10^-7^ |
| Parahippocampal gyrus | 0 | 0 | 1.00 | 0 | 0 | 7.98 | 0.006 | 0 | 0 | 2.84 | 0 | 0 | 7.98 | 0.004 |
| Amygdala | 0 | 0 | 2.89 | 0 | 0 | 3.87 | n.s. | 0 | 0 | 6.62 | 0 | 0 | 3.87 | 1.0x10^-6^ |
| Transverse temporal gyrus (heschl) | 0 | 0 | 6.82 | 0 | 0 | 6.10 | n.s. | 0 | 0 | 18.29 | 0 | 0 | 6.10 | 0.01 |
| Superior temporal gyrus | 0 | 0 | 6.92 | 0 | 0 | 4.89 | 0.01 | 0 | 0 | 11.64 | 0 | 0 | 4.79 | n.s. |
| Superior temporal pole | 0 | 0 | 5.28 | 0 | 0 | 4.63 | n.s. | 0 | 0 | 5.73 | 0 | 0 | 4.63 | 0.0004 |
| Middle temporal gyrus | 0 | 0 | 3.58 | 0 | 0 | 5.45 | 0.008 | 0 | 0 | 9.80 | 0 | 0 | 4.98 | n.s. |
| Middle temporal pole | 0 | 0 | 5.73 | 0 | 0 | 4.51 | n.s. | 0 | 0 | 4.92 | 0 | 0 | 4.51 | 2.1x10^-18^ |
| Inferior temporal gyrus | 0 | 0 | 0.49 | 0 | 0 | 7.28 | 0.01 | 0 | 0 | 9.25 | 0 | 0 | 7.28 | 5.0x10^-6^ |
| Insula | 0 | 0 | 6.64 | 0 | 0 | 6.59 | n.s. | 0 | 0 | 11.95 | 0 | 0 | 6.59 | 0.02 |
| **OCCIPITAL** | | | | | | | | | | | | | | |
| Calcarine sulcus | 0 | 0 | 0.26 | 0 | 0 | 0.77 | n.s. | 0 | 0 | 6.67 | 0 | 0 | 0.26 | n.s. |
| Cuneus | 0 | 0 | 0 | 0 | 0 | 0 | NA | 0 | 0 | 5.40 | 0 | 0 | 0.00 | n.s. |
| Lingual gyrus | 0 | 0 | 0.10 | 0 | 0 | 4.93 | 0.02 | 0 | 0 | 9.02 | 0 | 0 | 4.93 | n.s. |
| Superior occipital | 0 | 0 | 0.10 | 0 | 0 | 0.16 | n.s. | 0 | 0 | 5.63 | 0 | 0 | 0.16 | n.s. |
| Middle occipital gyrus | 0 | 0 | 0.12 | 0 | 0 | 1.17 | n.s. | 0 | 0 | 10.07 | 0 | 0 | 1.17 | 0.04 |
| Inferior occipital | 0 | 0 | 0.15 | 0 | 0 | 10.92 | n.s. | 0 | 0 | 11.24 | 0 | 0 | 2.99 | n.s. |
| Fusiform gyrus | 0 | 0 | 0.54 | 0 | 0 | 8.88 | 0.00006 | 0 | 0 | 6.04 | 0 | 0 | 8.88 | 9.0x10^-6^ |
| **PARIETAL** | | | | | | | | | | | | | | |
| Postcentral gyrus | 0 | 0 | 1.18 | 0 | 0 | 0.56 | n.s. | 0 | 0 | 8.05 | 0 | 0 | 0.42 | 0.01 |
| Superior parietal lobule | 0 | 0 | 0 | 0 | 0 | 0 | NA | 0 | 0 | 5.63 | 0 | 0 | 0 | n.s. |
| Inferior parietal lobule | 0 | 0 | 0 | 0 | 0 | 0 | NA | 0 | 0 | 9.91 | 0 | 0 | 0 | n.s. |
| Supramarginal gyrus | 0 | 0 | 4.31 | 0 | 0 | 0 | n.s. | 0 | 0 | 10.78 | 0 | 0 | 0 | 0.002 |
| Angular gyrus | 0 | 0 | 0.41 | 0 | 0 | 0.10 | n.s. | 0 | 0 | 10.40 | 0 | 0 | 0 | 0.01 |
| Precuneus | 0 | 0 | 0.10 | 0 | 0 | 0.10 | n.s. | 0 | 0 | 4.43 | 0 | 0 | 0.02 | n.s. |
| Paracentral lobule | 0 | 0 | 0 | 0 | 0 | 0.87 | n.s. | 0 | 0 | 7.58 | 0 | 0 | 0 | n.s. |
| **CINGULUM** | | | | | | | | | | | | | | |
| Anterior cingulate gyrus | 0 | 0 | 5.92 | 0 | 0 | 8.07 | n.s. | 0 | 0 | 7.26 | 0 | 0 | 1.78 | n.s. |
| Midcingulate gyrus | 0 | 0 | 0 | 0 | 0 | 0.11 | n.s. | 0 | 0 | 7.14 | 0 | 0 | 0 | n.s. |
| Posterior cingulate gyrus | 0 | 0 | 0 | 0 | 0 | 0 | NA | 0 | 0 | 6.48 | 0 | 0 | 0 | n.s. |
| **SUBCORTICAL GREY MATTER** | | | | | | | | | | | | | | |
| Caudate nucleus | 0 | 0 | 1.04 | 0 | 0 | 5.00 | 0.0005 | 0 | 0 | 10.38 | 0 | 0 | 5.00 | n.s. |
| Putamen | 0 | 0 | 0.53 | 0 | 0 | 8.74 | n.s. | 0 | 0 | 13.45 | 0 | 0 | 8.74 | n.s. |
| Globus pallidum | 0 | 0 | 3.37 | 0 | 0 | 8.46 | n.s. | 0 | 0 | 14.81 | 0 | 0 | 8.46 | n.s. |
| Thalamus | 0 | 0 | 1.45 | 0 | 0 | 8.49 | 0.0005 | 0 | 0 | 10.24 | 0 | 0 | 7.87 | 0.006 |
| **CEREBELLUM** | | | | | | | | | | | | | | |
| Crus I of cerebellar hemisphere | 0 | 0 | 0.10 | 0 | 0 | 1.97 | n.s. |  |  |  |  |  |  |  |
| Crus II of cerebellar hemisphere | 0 | 0 | 0 | 0 | 0 | 0 | NA |  |  |  |  |  |  |  |
| Lobule III of cerebellar hemisphere | 0 | 0 | 0 | 0 | 0 | 0.56 | n.s. |  |  |  |  |  |  |  |
| Lobule IV, V of cerebellar hemisphere | 0 | 0 | 1.91 | 0 | 0 | 2.41 | n.s. |  |  |  |  |  |  |  |
| Lobule VI of cerebellar hemisphere | 0 | 0 | 1.56 | 0 | 0 | 2.93 | n.s. |  |  |  |  |  |  |  |
| Lobule VIIB of cerebellar hemisphere | 0 | 0 | 0.11 | 0 | 0 | 0.71 | n.s. |  |  |  |  |  |  |  |
| Lobule VIII of cerebellar hemisphere | 0 | 0 | 0.11 | 0 | 0 | 2.73 | n.s. |  |  |  |  |  |  |  |
| Lobule IX of cerebellar hemisphere | 0 | 0 | 0 | 0 | 0 | 1.05 | n.s. |  |  |  |  |  |  |  |
| Lobule X of cerebellar hemisphere | 0 | 0 | 0 | 0 | 0 | 0 | NA |  |  |  |  |  |  |  |
| **WHITE MATTER** | | | | | | | | | | | | | | |
| Corticospinal tract | 0 | 0 | 0.88 | 0 | 0 | 0 | n.s. | 0 | 0 | 0 | 0 | 0 | 0 | N.A. |
| Medial lemniscus | 0 | 0 | 0.14 | 0 | 0 | 0 | n.s. | 0 | 0 | 0 | 0 | 0 | 0 | N.A. |
| Inferior cerebellar peduncle | 0 | 0 | 1.45 | 0 | 0 | 0 | n.s. | 0 | 0 | 0 | 0 | 0 | 0 | N.A. |
| Superior cerebellar peduncle | 0 | 0 | 0 | 0 | 0 | 0.20 | n.s. | 0 | 0 | 11.90 | 0 | 0 | 0.20 | n.s. |
| Cerebral peduncle | 0 | 0 | 0.97 | 0 | 0 | 0 | n.s. | 0 | 0 | 19.23 | 0 | 0 | 0 | 0.0006 |
| Anterior limb of internal capsule | 0 | 0 | 2.25 | 0 | 0 | 7.17 | n.s. | 0 | 0 | 15.97 | 0 | 0 | 7.17 | n.s. |
| Posterior limb of internal capsule | 0 | 0 | 1.57 | 0 | 0 | 8.42 | 0.0001 | 0 | 0 | 9.75 | 0 | 0 | 8.42 | n.s. |
| Retrolenticular part of internal capsule | 0 | 0 | 0.41 | 0 | 0 | 5.96 | n.s. | 0 | 0 | 28.67 | 0 | 0 | 5.96 | n.s. |
| Anterior corona radiata | 0 | 0 | 4.83 | 0 | 0 | 6.92 | n.s. | 0 | 0 | 12.95 | 0 | 0 | 6.03 | n.s. |
| Superior corona radiata | 0 | 0 | 0.56 | 0 | 0 | 4.20 | n.s. | 0 | 0 | 5.95 | 0 | 0 | 4.20 | 0.02 |
| Posterior corona radiata | 0 | 0 | 0.30 | 0 | 0 | 3.62 | n.s. | 0 | 0 | 13.81 | 0 | 0 | 3.62 | 0.03 |
| Posterior thalamic radiation | 0 | 0 | 0.70 | 0 | 0 | 3.47 | n.s. | 0 | 0 | 8.96 | 0 | 0 | 3.17 | n.s. |
| Sagittal stratum | 0 | 0 | 1.17 | 0 | 0 | 14.18 | n.s. | 0 | 0 | 31.87 | 0 | 0 | 12.34 | n.s. |
| External capsule | 0 | 0 | 1.36 | 0 | 0 | 8.84 | n.s. | 0 | 0 | 1.80 | 0 | 0 | 8.84 | 6.4x10^-18^ |
| Cingulum (cingulate gyrus) | 0 | 0 | 1.16 | 0 | 0 | 1.07 | n.s. | 0 | 0 | 1.88 | 0 | 0 | 0.00 | n.s. |
| Cingulum (hippocampus) | 0 | 0 | 0.43 | 0 | 0 | 11.73 | n.s. | 0 | 0 | 9.39 | 0 | 0 | 10.36 | n.s. |
| Fornix (cres) / Stria terminalis | 0 | 0 | 1.16 | 0 | 0 | 11.83 | n.s. | 0 | 0 | 72.33 | 0 | 0 | 6.49 | 0.005 |
| Superior longitudinal fasciculus | 0 | 0 | 0.82 | 0 | 0 | 2.42 | n.s. | 0 | 0 | 1.65 | 0 | 0 | 2.42 | n.s. |
| Superior fronto-occipital fasciculus | 0 | 0 | 0.59 | 0 | 0 | 15.19 | n.s. | 0 | 0 | 10.26 | 0 | 0 | 3.94 | n.s. |
| Uncinate fasciculus | 0 | 0 | 6.91 | 0 | 0 | 9.74 | n.s. | 0 | 0 | 15.79 | 0 | 0 | 9.74 | n.s. |
| Tapetum | 0 | 0 | 0 | 0 | 0 | 5.87 | n.s. | 0 | 0 | 0 | 0 | 0 | 5.87 | 5.9x10^-15^ |

^a^ p-value for Sign tests comparing the left- and right-hemisphere lesion volumes based on quantitative GM and WM analysis. The values being compared reflect the proportion of voxels in each AAL and JHU atlas area that are included in the lesion.

^b^ p-value for Wilcoxon rank-sum tests comparing lesion volumes based on quantitative GM and WM analysis between vascular lesional mania cases and an unselected sample of right-sided stroke described by Sperber & Karnath (124). The values being compared reflect the median proportion of voxels in each AAL and JHU atlas area that are included in the lesion.

Statistical significance was defined using a False Discovery Rate (FDR) of 0,01, according to Benjamini-Hochberg (123).

Max – Maximum; Min - Minimum; NA – Not Applicable; n.s. – non-significant;

**Table S9 –** Comparison of Right Hemisphere Lesion with a Control Sample of Stroke Lesions using percentage of patients lesioned in a given area

| **Area** | **Patients (%)** | | |
| --- | --- | --- | --- |
|  | **Sperber et al (N=439)** | **Barahona-Corrêa  et al (N=29)** | **p (Fisher's exact test)^a^** |
|  |  |  |  |
| **GREY MATTER** | | | |
| **FRONTAL** | | | |
| Precentral gyrus | 13.2 | 3.4 | n.s. |
| Superior frontal gyrus | 2.5 | 13.8 | 0.03 |
| Superior frontal gyrus, orbital part | 2.1 | 10.3 | n.s. |
| Middle frontal gyrus | 7.7 | 10.3 | n.s. |
| Middle frontal gyrus, orbital part | 3.9 | 6.9 | n.s. |
| Inferior frontal gyrus, pars opercularis | 23.2 | 3.4 | 0.006 |
| Inferior frontal gyrus, pars triangularis | 15.3 | 3.4 | n.s. |
| Inferior frontal gyrus, pars orbitalis | 11.8 | 10.3 | n.s. |
| Rolandic operculum | 30.8 | 6.9 | 0.0009 |
| Supplementary motor area | 2.3 | 0 | n.s. |
| Olfactory cortex | 3.9 | 6.9 | n.s. |
| Medial frontal gyrus | 1.4 | 6.9 | n.s. |
| Medial orbitofrontal cortex | 1.1 | 10.3 | 0.01 |
| Rectus gyrus | 0.5 | 6.9 | n.s. |
| **TEMPORAL AND INSULA** | | | |
| Hippocampus | 15 | 44.8 | 0.001 |
| Parahippocampal gyrus | 5.5 | 20.7 | n.s. |
| Amygdala | 16.9 | 17.2 | n.s. |
| Transverse temporal gyrus (Heschl) | 32.3 | 6.9 | 0.002 |
| Superior temporal gyrus | 28.7 | 34 | n.s. |
| Superior temporal pole | 16.4 | 37.9 | 0.03 |
| Middle temporal gyrus | 22.6 | 41.4 | n.s. |
| Middle temporal pole | 6.4 | 27.6 | 0.0063 |
| Inferior temporal gyrus | 8.9 | 37.9 | 0.0007 |
| Insula | 36.0 | 27.6 | n.s. |
| **OCCIPITAL** | | | |
| Calcarine sulcus | 9.3 | 6.9 | n.s. |
| Cuneus | 6.8 | 0 | n.s. |
| Lingual gyrus | 7.7 | 10.3 | n.s. |
| Superior occipital | 8 | 3.4 | n.s. |
| Middle occipital gyrus | 12.3 | 3.4 | n.s. |
| Inferior occipital | 8.4 | 10.3 | n.s. |
| Fusiform gyrus | 4.6 | 27.6 | 0.0005 |
| **PARIETAL** | | | |
| Postcentral gyrus | 12.1 | 3.4 | n.s. |
| Superior parietal lobule | 5.7 | 0 | n.s. |
| Inferior parietal lobule | 15.9 | 0 | 0.005 |
| Supramarginal gyrus | 24.6 | 0 | 0.0002 |
| Angular gyrus | 18.5 | 0 | 0.003 |
| Precuneus | 1.6 | 0 | n.s. |
| Paracentral lobule | 2.1 | 0 | n.s. |
| **CINGULUM** | | | |
| Anterior cingulate gyrus | 1.1 | 10.3 | n.s. |
| Midcingulate gyrus | 1.8 | 0 | n.s. |
| Posterior cingulate gyrus | 0.7 | 0 | n.s. |
| **SUBCORTICAL GREY MATTER** | | | |
| Caudate nucleus | 18.9 | 24.1 | n.s. |
| Putamen | 32.8 | 31.0 | n.s. |
| Globus pallidum | 22.1 | 20.7 | n.s. |
| Thalamus | 7.7 | 44.8 | 0.000008 |
| **WHITE MATTER** | | | |
| Corticospinal tract | 0 | 0 | N.A. |
| Medial lemniscus | 0 | 0 | N.A. |
| Inferior cerebellar peduncle | 0 | 0 | N.A. |
| Superior cerebellar peduncle | 0 | 3.4 | n.s. |
| Cerebral peduncle | 3 | 0 | n.s. |
| Anterior limb of internal capsule | 24.4 | 34.5 | n.s. |
| Posterior limb of internal capsule | 26.9 | 48 | n.s. |
| Retrolenticular part of internal capsule | 28.7 | 34.5 | n.s. |
| Anterior corona radiata | 20.7 | 34.5 | n.s. |
| Superior corona radiata | 31.9 | 13.8 | n.s. |
| Posterior corona radiata | 25.1 | 17.2 | n.s. |
| Posterior thalamic radiation | 23.2 | 27.6 | n.s. |
| Sagittal stratum | 19.1 | 31.0 | n.s. |
| External capsule | 36.7 | 41.4 | n.s. |
| Cingulum (cingulate gyrus) | 1.6 | 7 | n.s. |
| Cingulum (hippocampus) | 5 | 17.2 | n.s. |
| Fornix (cres) / Stria terminalis | 16.6 | 24.1 | n.s. |
| Superior longitudinal fasciculus | 34.2 | 20.7 | n.s. |
| Superior fronto-occipital fasciculus | 30.8 | 10.3 | n.s. |
| Uncinate fasciculus | 23.9 | 20.7 | n.s. |
| Tapetum | 16.6 | 13.8 | n.s. |

^a^ p-value for Fisher’s exact tests comparing lesion frequency per AAL and JHU atlas area between vascular lesional mania cases and an unselected sample of right-sided stroke described by Sperber & Karnath(124). For both analyses, statistical significance was defined using a False Discovery Rate (FDR) of 0.1, according to Benjamini-Hochberg(123).

NA – Not Applicable; n.s. – non-significant; SD – Standard Deviation;

We compared the proportion of mania patients with lesions in each area of the GM and WM atlases to that in a sample of 439 right-hemisphere stroke patients(124). Since in the sample of right-hemisphere stroke patients we had access to 3-dimensional (3D) MRI images, while in lesional mania patients we had access to a maximum of 5 brain scan slices per subject, the mean number of lesioned areas was much higher in the former, rendering a direct comparison uninterpretable. To allow for comparisons between right-hemisphere stroke and lesional mania images, the presence of a lesion in a particular brain area was considered according to differing criteria in each of the two groups. Specifically, lesional thresholds, defined as a proportion of the total volume of that area that was lesioned, were defined to obtain a similar mean number of affected brain areas per individual in each group, while maintaining a minimum number of 1 lesioned area per subject across both groups. For lesional mania cases, where few brain-image slices were available in each patient, an area was considered to be lesioned when 0.1% or more of the voxels comprising that area, with a minimum of 5 voxels, were lesioned. In right-hemisphere stroke patients, where we had access to full MRI scans, an area was considered lesioned when 15% or more of the total number of voxels were lesioned. Using these thresholds, the mean number of affected brain areas per individual in each group was similar (9.0 ± 8.8 for right-hemisphere stroke and 9.1 ± 9.1 for lesional mania; p=0.97).

**Table S10** – Exploratory analyses using only a selected subpopulation of lesional mania cases

| **Area** | **% lesioned voxels^a^ (mean ± SD; N=16)** | | | | | **% lesioned voxels^a^ (mean ± SD)** | | | | |
| --- | --- | --- | --- | --- | --- | --- | --- | --- | --- | --- |
|  | **Left** | | **Right** | | **p**  **(Sign test)^b^** | **Sperber et al (N=439)** | | **Barahona-Corrêa et al (N=12)** | | **p (Rank-sum test)^c^** |
|  | **Mean** | **SD** | **Mean** | **SD** |  | **Mean** | **SD** | **Mean** | **SD** |  |
| **GREY MATTER** | | | | | | | | | | |
| **FRONTAL** | | | | | | | | | | |
| Precentral gyrus | 0 | 0 | 0 | 0 | NA | 0.23 | 0.70 | 0 | 0 | n.s. |
| Superior frontal gyrus | 0 | 0 | 0.0004 | 0.002 | n.s. | 0.05 | 0.30 | 0 | 0 | n.s. |
| Superior frontal gyrus, orbital part | 0.002 | 0.01 | 0 | 0 | n.s. | 0.03 | 0.25 | 0 | 0 | n.s. |
| Middle frontal gyrus | 0 | 0 | 0.03 | 0.11 | n.s. | 0.17 | 0.63 | 0.04 | 0.12 | n.s. |
| Middle frontal gyrus, orbital part | 0 | 0 | 0 | 0 | NA | 0.07 | 0.40 | 0 | 0 | n.s. |
| Inferior frontal gyrus, pars opercularis | 0.03 | 0.13 | 0 | 0 | n.s. | 1.04 | 2.24 | 0 | 0 | 0.01 |
| Inferior frontal gyrus, pars triangularis | 0.02 | 0.06 | 0 | 0 | n.s. | 0.73 | 1.97 | 0 | 0 | 0.03 |
| Inferior frontal gyrus, pars orbitalis | 0.09 | 0.34 | 0 | 0 | n.s. | 0.17 | 0.83 | 0 | 0 | n.s. |
| Rolandic operculum | 0.33 | 1.30 | 0.05 | 0.21 | n.s. | 1.72 | 3.40 | 0.07 | 0.24 | 0.04 |
| Supplementary motor area | 0 | 0 | 0 | 0 | NA | 0.04 | 0.46 | 0 | 0 | n.s. |
| Olfactory cortex | 0 | 0 | 0 | 0 | NA | 0.03 | 0.27 | 0 | 0 | n.s. |
| Medial frontal gyrus | 0 | 0 | 0 | 0 | NA | 0.04 | 0.39 | 0 | 0 | n.s. |
| Medial orbitofrontal cortex | 0 | 0 | 0 | 0 | NA | 0.04 | 0.42 | 0 | 0 | n.s. |
| Rectus gyrus | 0 | 0 | 0 | 0 | NA | 0.00 | 0.07 | 0 | 0 | n.s. |
| **TEMPORAL AND INSULA** | | | | | | | | | | |
| Hippocampus | 0 | 0 | 0.47 | 0.3 | 0.03 | 0.21 | 0.78 | 0.20 | 0.32 | 0.005 |
| Parahippocampal gyrus | 0.002 | 0.01 | 0.7 | 0.12 | n.s. | 0.09 | 0.39 | 0.04 | 0.14 | n.s. |
| Amygdala | 0 | 0 | 0.28 | 0.97 | n.s. | 0.07 | 0.58 | 0.38 | 1.12 | 0.006 |
| Transverse temporal gyrus (Heschl) | 0.43 | 1.70 | 0.38 | 1.52 | n.s. | 1.73 | 3.51 | 0.51 | 1.76 | n.s. |
| Superior temporal gyrus | 0.43 | 1.73 | 0.22 | 0.73 | n.s. | 1.33 | 2.57 | 0.30 | 0.84 | n.s. |
| Superior temporal pole | 0.34 | 1.32 | 0.27 | 0.62 | n.s. | 0.16 | 0.61 | 0.36 | 0.70 | n.s. |
| Middle temporal gyrus | 0.35 | 1.01 | 0.47 | 1.01 | n.s. | 0.75 | 1.69 | 0.63 | 1.13 | n.s. |
| Middle temporal pole | 0.41 | 1.16 | 0.18 | 0.38 | n.s. | 0.02 | 0.26 | 0.24 | 0.42 | n.s. |
| Inferior temporal gyrus | 0.03 | 0.09 | 0.36 | 0.84 | n.s. | 0.13 | 0.67 | 0.48 | 0.94 | 0.009 |
| Insula | 0.26 | 1.02 | 0.06 | 0.16 | n.s. | 1.47 | 2.59 | 0.08 | 0.19 | 0.02 |
| **OCCIPITAL** | | | | | | | | | | |
| Calcarine sulcus | 0.004 | 0.018 | 0.01 | 0.03 | n.s. | 0.23 | 0.88 | 0.01 | 0.03 | n.s. |
| Cuneus | 0.002 | 0.006 | 0 | 0 | n.s. | 0.09 | 0.50 | 0 | 0 | n.s. |
| Lingual gyrus | 0 | 0 | 0.31 | 1.23 | n.s. | 0.37 | 1.40 | 0.41 | 1.42 | n.s. |
| Superior occipital | 0 | 0 | 0.01 | 0.04 | n.s. | 0.17 | 0.69 | 0.01 | 0.05 | n.s. |
| Middle occipital gyrus | 0.01 | 0.03 | 0.07 | 0.29 | n.s. | 0.40 | 1.19 | 0.10 | 0.34 | n.s. |
| Inferior occipital | 0 | 0 | 0.01 | 0.04 | n.s. | 0.26 | 1.21 | 0.01 | 0.05 | n.s. |
| Fusiform gyrus | 0 | 0 | 0.12 | 0.25 | n.s. | 0.10 | 0.47 | 0.16 | 0.28 | 0.01 |
| **PARIETAL** | | | | | | | | | | |
| Postcentral gyrus | 0.07 | 0.30 | 0.0002 | 0.001 | n.s. | 0.26 | 0.72 | 0 | 0 | n.s. |
| Superior parietal lobule | 0 | 0 | 0 | 0 | NA | 0.04 | 0.39 | 0 | 0 | n.s. |
| Inferior parietal lobule | 0 | 0 | 0 | 0 | NA | 0.17 | 0.89 | 0 | 0 | n.s. |
| Supramarginal gyrus | 0.27 | 1.08 | 0 | 0 | n.s. | 0.64 | 1.67 | 0 | 0 | 0.04 |
| Angular gyrus | 0.03 | 0.10 | 0 | 0 | n.s. | 0.36 | 1.20 | 0 | 0 | n.s. |
| Precuneus | 0.0011 | 0.004 | 0 | 0 | n.s. | 0.05 | 0.28 | 0 | 0 | n.s. |
| Paracentral lobule | 0 | 0 | 0 | 0 | NA | 0.03 | 0.42 | 0 | 0 | n.s. |
| **CINGULUM** | | | | | | | | | | |
| Anterior cingulate gyrus | 0 | 0 | 0 | 0 | NA | 0.06 | 0.55 | 0 | 0 | n.s. |
| Midcingulate gyrus | 0 | 0 | 0 | 0 | NA | 0.08 | 0.56 | 0 | 0 | n.s. |
| Posterior cingulate gyrus | 0 | 0 | 0 | 0 | NA | 0.02 | 0.31 | 0 | 0 | n.s. |
| **SUBCORTICAL GREY MATTER** | | | | | | | | | | |
| Caudate nucleus | 0.003 | 0.01 | 0.36 | 0.71 | n.s. | 0.71 | 1.68 | 0.49 | 0.79 | n.s. |
| Putamen | 0.03 | 0.13 | 0.24 | 0.56 | n.s. | 1.65 | 3.08 | 0.33 | 0.63 | n.s. |
| Globus pallidum | 0 | 0 | 0.15 | 0.52 | n.s. | 1.15 | 2.90 | 0.20 | 0.59 | n.s. |
| Thalamus | 0.06 | 0.26 | 0.41 | 0.87 | 0.03 | 0.35 | 1.12 | 0.55 | 0.97 | 0.04 |
| **CEREBELLUM** | | | | | | | | | | |
| Crus I of cerebellar hemisphere | 0 | 0 | 0 | 0 | NA |  |  |  |  |  |
| Crus II of cerebellar hemisphere | 0 | 0 | 0 | 0 | NA |  |  |  |  |  |
| Lobule III of cerebellar hemisphere | 0 | 0 | 0 | 0 | NA |  |  |  |  |  |
| Lobule IV, V of cerebellar hemisphere | 0 | 0 | 0 | 0 | NA |  |  |  |  |  |
| Lobule VI of cerebellar hemisphere | 0 | 0 | 0 | 0 | NA |  |  |  |  |  |
| Lobule VIIB of cerebellar hemisphere | 0 | 0 | 0.04 | 0.18 | n.s. |  |  |  |  |  |
| Lobule VIII of cerebellar hemisphere | 0 | 0 | 0.17 | 0.68 | n.s. |  |  |  |  |  |
| Lobule IX of cerebellar hemisphere | 0 | 0 | 0.07 | 0.26 | n.s. |  |  |  |  |  |
| Lobule X of cerebellar hemisphere | 0 | 0 | 0 | 0 | NA |  |  |  |  |  |
| **WHITE MATTER** | | | | | | | | | | |
| Corticospinal tract | 0.05 | 0.22 | 0 | 0 | n.s. | 0 | 0 | 0 | 0 | N.A. |
| Medial lemniscus | 0.01 | 0.04 | 0 | 0 | n.s. | 0 | 0 | 0 | 0 | N.A. |
| Inferior cerebellar peduncle | 0 | 0 | 0 | 0 | NA | 0 | 0 | 0 | 0 | N.A. |
| Superior cerebellar peduncle | 0 | 0 | 0 | 0 | NA | 0.09 | 0.87 | 0 | 0 | n.s. |
| Cerebral peduncle | 0.06 | 0.24 | 0 | 0 | n.s. | 1.95 | 4.10 | 0 | 0 | 0.03 |
| Anterior limb of internal capsule | 0.03 | 0.12 | 0.47 | 1.20 | n.s. | 1.60 | 3.29 | 0.62 | 1.36 | n.s. |
| Posterior limb of internal capsule | 0.003 | 0.01 | 0.43 | 0.98 | n.s. | 0.87 | 1.77 | 0.58 | 1.10 | n.s. |
| Retrolenticular part of internal capsule | 0 | 0 | 0.35 | 1.16 | n.s. | 2.14 | 5.06 | 0.46 | 1.33 | n.s. |
| Anterior corona radiata | 0.002 | 0.01 | 0.12 | 0.47 | n.s. | 0.98 | 2.24 | 0.16 | 0.54 | n.s. |
| Superior corona radiata | 0.02 | 0.09 | 0.03 | 0.12 | n.s. | 0.35 | 0.86 | 0.04 | 0.14 | n.s. |
| Posterior corona radiata | 0.01 | 0.05 | 0.01 | 0.05 | n.s. | 0.86 | 2.01 | 0.02 | 0.06 | n.s. |
| Posterior thalamic radiation | 0.06 | 0.18 | 0.31 | 0.85 | n.s. | 0.30 | 1.09 | 0.42 | 0.97 | n.s. |
| Sagittal stratum | 0.07 | 0.29 | 0.21 | 0.49 | n.s. | 3.92 | 6.72 | 0.28 | 0.56 | n.s. |
| External capsule | 0.05 | 0.16 | 0.18 | 0.46 | n.s. | 0.02 | 0.14 | 0.23 | 0.52 | 5.6x10-9 |
| Cingulum (cingulate gyrus) | 0 | 0 | 0 | 0 | NA | 0.07 | 0.29 | 0 | 0 | n.s. |
| Cingulum (hippocampus) | 0 | 0 | 0 | 0 | NA | 0.35 | 1.28 | 0 | 0 | n.s. |
| Fornix (cres) / Stria terminalis | 0 | 0 | 0.07 | 0.19 | n.s. | 6.30 | 13.04 | 0.10 | 0.22 | 0.05 |
| Superior longitudinal fasciculus | 0.05 | 0.20 | 0.12 | 0.32 | n.s. | 0.11 | 0.35 | 0.16 | 0.37 | n.s. |
| Superior fronto-occipital fasciculus | 0 | 0 | 0.10 | 0.39 | n.s. | 0.17 | 1.17 | 0.13 | 0.46 | n.s. |
| Uncinate fasciculus | 0 | 0 | 0.67 | 2.43 | n.s. | 1.03 | 2.91 | 0.90 | 2.80 | n.s. |
| Tapetum | 0 | 0 | 0.30 | 0.88 | n.s. | 0 | 0 | 0.41 | 1.00 | 1.1x10-17 |

^a^ Displayed values are means and standard deviations. Please see Supplementary Table S11 for medians, minimums and maximums.

^b^ p-value for Sign tests comparing the left- and right-hemisphere lesion volumes based on quantitative GM and WM analysis. The values being compared reflect the proportion of voxels in each AAL and JHU atlas area that are included in the lesion. Statistical significance was defined using a False Discovery Rate (FDR) of 0,01, according to Benjamini-Hochberg (123).

^c^ p-value for Wilcoxon rank-sum tests comparing lesion volumes based on quantitative GM and WM analysis between vascular lesional mania cases and an unselected sample of right-sided stroke described by Sperber & Karnath (124). The values being compared reflect the median proportion of voxels in each AAL and JHU atlas area that are included in the lesion.

NA – Not Applicable; n.s. – non-significant; SD – Standard Deviation;

This exploratory analysis only considers patients with no personal or family history of psychiatric disorder, with vascular lesion etiology, that fulfill DSM 5 criteria for manic episode, and with CT or MRI images for analysis (i.e. all the group diagrams were excluded). Due to the exploratory nature of these analyses, and the low number of patients, analyses were not corrected for multiple comparisons.

**Table S11** – Median, minimum and maximum from the comparison of Right vs. Left Hemisphere Lesions and of Right Hemisphere Stroke Lesions with a Control Sample of Stroke: exploratory analyses using only a selected subpopulation of lesional mania cases (see table S10 for info on subpopulation).

| **Area** | **% lesioned voxels (N=16)** | | | | | | | **% lesioned voxels** | | | | | | |
| --- | --- | --- | --- | --- | --- | --- | --- | --- | --- | --- | --- | --- | --- | --- |
|  | **Left** | | | **Right** | | | **p  (Sign Test)^a^** | **Sperber et al (N=439)** | | | **Barahona-Corrêa et al (N=12)** | | | **p (Rank-sum test)^b^** |
|  | **Median** | **Min** | **Max** | **Median** | **Min** | **Max** |  | **Median** | **Min** | **Max** | **Median** | **Min** | **Max** |  |
| **GREY MATTER** | | | | | | | | | | | | | | |
| **FRONTAL** | | | | | | | | | | | | | | |
| Precentral gyrus | 0 | 0 | 0 | 0 | 0 | 0 | NA | 0 | 0 | 7.10 | 0 | 0 | 0.00 | n.s. |
| Superior frontal gyrus | 0 | 0 | 0 | 0 | 0 | 0.01 | n.s. | 0 | 0 | 3.19 | 0 | 0 | 0.01 | n.s. |
| Superior frontal gyrus, orbital part | 0 | 0 | 0.03 | 0 | 0 | 0 | n.s. | 0 | 0 | 3.02 | 0 | 0 | 0.00 | n.s. |
| Middle frontal gyrus | 0 | 0 | 0 | 0 | 0 | 0.42 | n.s. | 0 | 0 | 5.17 | 0 | 0 | 0.42 | n.s. |
| Middle frontal gyrus, orbital part | 0 | 0 | 0 | 0 | 0 | 0 | NA | 0 | 0 | 3.79 | 0 | 0 | 0.00 | n.s. |
| Inferior frontal gyrus, pars opercularis | 0 | 0 | 0.51 | 0 | 0 | 0 | n.s. | 0 | 0 | 11.71 | 0 | 0 | 0.00 | 0.01 |
| Inferior frontal gyrus, pars triangularis | 0 | 0 | 0.25 | 0 | 0 | 0 | n.s. | 0 | 0 | 11.67 | 0 | 0 | 0.00 | 0.03 |
| Inferior frontal gyrus, pars orbitalis | 0 | 0 | 1.37 | 0 | 0 | 0 | n.s. | 0 | 0 | 8.74 | 0 | 0 | 0.00 | n.s. |
| Rolandic opercullum | 0 | 0 | 5.21 | 0 | 0 | 0.83 | n.s. | 0 | 0 | 12.81 | 0 | 0 | 0.83 | 0.04 |
| Supplementary motor area | 0 | 0 | 0 | 0 | 0 | 0 | NA | 0 | 0 | 8.83 | 0 | 0 | 0.00 | n.s. |
| Olfactory cortex | 0 | 0 | 0 | 0 | 0 | 0 | NA | 0 | 0 | 3.54 | 0 | 0 | 0.00 | n.s. |
| Medial frontal gyrus | 0 | 0 | 0 | 0 | 0 | 0 | NA | 0 | 0 | 5.02 | 0 | 0 | 0.00 | n.s. |
| Medial orbitofrontal cortex | 0 | 0 | 0 | 0 | 0 | 0 | NA | 0 | 0 | 5.66 | 0 | 0 | 0.00 | n.s. |
| Rectus gyrus | 0 | 0 | 0 | 0 | 0 | 0 | NA | 0 | 0 | 1.38 | 0 | 0 | 0.00 | n.s. |
| **TEMPORAL AND INSULA** | | | | | | | | | | | | | | |
| Hippocampus | 0 | 0 | 0 | 0 | 0 | 1.01 | 0.03 | 0 | 0 | 7.47 | 0.05 | 0 | 1.01 | 0.005 |
| Parahippocampal gyrus | 0 | 0 | 0.03 | 0 | 0 | 0.50 | n.s. | 0 | 0 | 2.84 | 0 | 0 | 0.50 | n.s. |
| Amygdala | 0 | 0 | 0 | 0 | 0 | 3.87 | n.s. | 0 | 0 | 6.62 | 0 | 0 | 3.87 | 0.006 |
| Transverse temporal gyrus (Heschl) | 0 | 0 | 6.82 | 0 | 0 | 6.10 | n.s. | 0 | 0 | 18.29 | 0 | 0 | 6.10 | n.s. |
| Superior temporal gyrus | 0 | 0 | 6.92 | 0 | 0 | 2.93 | n.s. | 0 | 0 | 11.64 | 0 | 0 | 2.93 | n.s. |
| Superior temporal pole | 0 | 0 | 5.28 | 0 | 0 | 1.92 | n.s. | 0 | 0 | 5.73 | 0 | 0 | 1.92 | n.s. |
| Middle temporal gyrus | 0 | 0 | 3.58 | 0 | 0 | 3.12 | n.s. | 0 | 0 | 9.80 | 0 | 0 | 3.12 | n.s. |
| Middle temporal pole | 0 | 0 | 4.01 | 0 | 0 | 1.26 | n.s. | 0 | 0 | 4.92 | 0 | 0 | 1.26 | n.s. |
| Inferior temporal gyrus | 0 | 0 | 0.34 | 0 | 0 | 2.55 | n.s. | 0 | 0 | 9.25 | 0 | 0 | 2.55 | 0.009 |
| Insula | 0 | 0 | 4.07 | 0 | 0 | 0.60 | n.s. | 0 | 0 | 11.95 | 0 | 0 | 0.60 | 0.02 |
| **OCCIPITAL** | | | | | | | | | | | | | | |
| Calcarine sulcus | 0 | 0 | 0.07 | 0 | 0 | 0.12 | n.s. | 0 | 0 | 6.67 | 0 | 0 | 0.12 | n.s. |
| Cuneus | 0 | 0 | 0.02 | 0 | 0 | 0 | n.s. | 0 | 0 | 5.40 | 0 | 0 | 0.00 | n.s. |
| Lingual gyrus | 0 | 0 | 0 | 0 | 0 | 4.93 | n.s. | 0 | 0 | 9.02 | 0 | 0 | 4.93 | n.s. |
| Superior occipital | 0 | 0 | 0 | 0 | 0 | 0.16 | n.s. | 0 | 0 | 5.63 | 0 | 0 | 0.16 | n.s. |
| Middle occipital gyrus | 0 | 0 | 0.12 | 0 | 0 | 1.17 | n.s. | 0 | 0 | 10.07 | 0 | 0 | 1.17 | n.s. |
| Inferior occipital | 0 | 0 | 0 | 0 | 0 | 0.16 | n.s. | 0 | 0 | 11.24 | 0 | 0 | 0.16 | n.s. |
| Fusiform gyrus | 0 | 0 | 0 | 0 | 0 | 0.81 | n.s. | 0 | 0 | 6.04 | 0 | 0 | 0.81 | 0.01 |
| **PARIETAL** | | | | | | | | | | | | | | |
| Postcentral gyrus | 0 | 0 | 1.18 | 0 | 0 | 0.003 | n.s. | 0 | 0 | 8.05 | 0 | 0 | 0.00 | n.s. |
| Superior parietal lobule | 0 | 0 | 0 | 0 | 0 | 0 | NA | 0 | 0 | 5.63 | 0 | 0 | 0 | n.s. |
| Inferior parietal lobule | 0 | 0 | 0 | 0 | 0 | 0 | NA | 0 | 0 | 9.91 | 0 | 0 | 0 | n.s. |
| Supramarginal gyrus | 0 | 0 | 4.31 | 0 | 0 | 0 | n.s. | 0 | 0 | 10.78 | 0 | 0 | 0 | 0.04 |
| Angular gyrus | 0 | 0 | 0.41 | 0 | 0 | 0 | n.s. | 0 | 0 | 10.40 | 0 | 0 | 0 | n.s. |
| Precuneus | 0 | 0 | 0.02 | 0 | 0 | 0 | n.s. | 0 | 0 | 4.43 | 0 | 0 | 0.00 | n.s. |
| Paracentral lobule | 0 | 0 | 0 | 0 | 0 | 0 | NA | 0 | 0 | 7.58 | 0 | 0 | 0 | n.s. |
| **CINGULUM** | | | | | | | | | | | | | | |
| Anterior cingulate gyrus | 0 | 0 | 0 | 0 | 0 | 0 | NA | 0 | 0 | 7.26 | 0 | 0 | 0.00 | n.s. |
| Midcingulate gyrus | 0 | 0 | 0 | 0 | 0 | 0 | NA | 0 | 0 | 7.14 | 0 | 0 | 0 | n.s. |
| Posterior cingulate gyrus | 0 | 0 | 0 | 0 | 0 | 0 | NA | 0 | 0 | 6.48 | 0 | 0 | 0 | n.s. |
| **SUBCORTICAL GREY MATTER** | | | | | | | | | | | | | | |
| Caudate nucleus | 0 | 0 | 0.05 | 0 | 0 | 2.19 | n.s. | 0 | 0 | 10.38 | 0 | 0 | 2.19 | n.s. |
| Putamen | 0 | 0 | 0.53 | 0 | 0 | 1.87 | n.s. | 0 | 0 | 13.45 | 0 | 0 | 1.87 | n.s. |
| Globus pallidum | 0 | 0 | 0 | 0 | 0 | 2.06 | n.s. | 0 | 0 | 14.81 | 0 | 0 | 2.06 | n.s. |
| Thalamus | 0 | 0 | 1.02 | 0 | 0 | 2.94 | 0.03 | 0 | 0 | 10.24 | 0 | 0 | 2.94 | 0.04 |
| **CEREBELLUM** | | | | | | | | | | | | | | |
| Crus I of cerebellar hemisphere | 0 | 0 | 0 | 0 | 0 | 0 | NA |  |  |  |  |  |  |  |
| Crus II of cerebellar hemisphere | 0 | 0 | 0 | 0 | 0 | 0 | NA |  |  |  |  |  |  |  |
| Lobule III of cerebellar hemisphere | 0 | 0 | 0 | 0 | 0 | 0 | NA |  |  |  |  |  |  |  |
| Lobule IV, V of cerebellar hemisphere | 0 | 0 | 0 | 0 | 0 | 0 | NA |  |  |  |  |  |  |  |
| Lobule VI of cerebellar hemisphere | 0 | 0 | 0 | 0 | 0 | 0 | NA |  |  |  |  |  |  |  |
| Lobule VIIB of cerebellar hemisphere | 0 | 0 | 0 | 0 | 0 | 0.71 | n.s. |  |  |  |  |  |  |  |
| Lobule VIII of cerebellar hemisphere | 0 | 0 | 0 | 0 | 0 | 2.73 | n.s. |  |  |  |  |  |  |  |
| Lobule IX of cerebellar hemisphere | 0 | 0 | 0 | 0 | 0 | 1.05 | n.s. |  |  |  |  |  |  |  |
| Lobule X of cerebellar hemisphere | 0 | 0 | 0 | 0 | 0 | 0 | NA |  |  |  |  |  |  |  |
| **WHITE MATTER** | | | | | | | | | | | | | | |
| Corticospinal tract | 0 | 0 | 0.88 | 0 | 0 | 0 | n.s. | 0 | 0 | 0 | 0 | 0 | 0 | N.A. |
| Medial lemniscus | 0 | 0 | 0.14 | 0 | 0 | 0 | n.s. | 0 | 0 | 0 | 0 | 0 | 0 | N.A. |
| Inferior cerebellar peduncle | 0 | 0 | 0 | 0 | 0 | 0 | NA | 0 | 0 | 0 | 0 | 0 | 0 | N.A. |
| Superior cerebellar peduncle | 0 | 0 | 0 | 0 | 0 | 0 | NA | 0 | 0 | 11.90 | 0 | 0 | 0.00 | n.s. |
| Cerebral peduncle | 0 | 0 | 0.97 | 0 | 0 | 0 | n.s. | 0 | 0 | 19.23 | 0 | 0 | 0 | 0.03 |
| Anterior limb of internal capsule | 0 | 0 | 0.46 | 0 | 0 | 4.68 | n.s. | 0 | 0 | 15.97 | 0 | 0 | 4.68 | n.s. |
| Posterior limb of internal capsule | 0 | 0 | 0.05 | 0 | 0 | 3.81 | n.s. | 0 | 0 | 9.75 | 0 | 0 | 3.81 | n.s. |
| Retrolenticular part of internal capsule | 0 | 0 | 0 | 0 | 0 | 4.65 | n.s. | 0 | 0 | 28.67 | 0 | 0 | 4.65 | n.s. |
| Anterior corona radiata | 0 | 0 | 0.03 | 0 | 0 | 1.87 | n.s. | 0 | 0 | 12.95 | 0 | 0 | 1.87 | n.s. |
| Superior corona radiata | 0 | 0 | 0.35 | 0 | 0 | 0.48 | n.s. | 0 | 0 | 5.95 | 0 | 0 | 0.48 | n.s. |
| Posterior corona radiata | 0 | 0 | 0.22 | 0 | 0 | 0.21 | n.s. | 0 | 0 | 13.81 | 0 | 0 | 0.21 | n.s. |
| Posterior thalamic radiation | 0 | 0 | 0.70 | 0 | 0 | 3.17 | n.s. | 0 | 0 | 8.96 | 0 | 0 | 3.17 | n.s. |
| Sagittal stratum | 0 | 0 | 1.17 | 0 | 0 | 1.57 | n.s. | 0 | 0 | 31.87 | 0 | 0 | 1.57 | n.s. |
| External capsule | 0 | 0 | 0.63 | 0 | 0 | 1.78 | n.s. | 0 | 0 | 1.80 | 0 | 0 | 1.78 | 5.6x10^-9^ |
| Cingulum (cingulate gyrus) | 0 | 0 | 0 | 0 | 0 | 0 | NA | 0 | 0 | 1.88 | 0 | 0 | 0.00 | n.s. |
| Cingulum (hippocampus) | 0 | 0 | 0 | 0 | 0 | 0 | NA | 0 | 0 | 9.39 | 0 | 0 | 0.00 | n.s. |
| Fornix (cres) / Stria terminalis | 0 | 0 | 0 | 0 | 0 | 0.71 | n.s. | 0 | 0 | 72.33 | 0 | 0 | 0.71 | 0.05 |
| Superior longitudinal fasciculus | 0 | 0 | 0.82 | 0 | 0 | 1.00 | n.s. | 0 | 0 | 1.65 | 0 | 0 | 1.00 | n.s. |
| Superior fronto-occipital fasciculus | 0 | 0 | 0 | 0 | 0 | 1.58 | n.s. | 0 | 0 | 10.26 | 0 | 0 | 1.58 | n.s. |
| Uncinate fasciculus | 0 | 0 | 0 | 0 | 0 | 9.74 | n.s. | 0 | 0 | 15.79 | 0 | 0 | 9.74 | n.s. |
| Tapetum | 0 | 0 | 0 | 0 | 0 | 3.19 | n.s. | 0 | 0 | 0 | 0 | 0 | 3.19 | 1.1x10^-17^ |

^a^ p-value for Sign tests comparing the left- and right-hemisphere lesion volumes based on quantitative GM and WM analysis. The values being compared reflect the proportion of voxels in each AAL and JHU atlas area that are included in the lesion. Statistical significance was defined using a False Discovery Rate (FDR) of 0,01, according to Benjamini-Hochberg (123).

^b^ p-value for Wilcoxon rank-sum tests comparing lesion volumes based on quantitative GM and WM analysis between vascular lesional mania cases and an unselected sample of right-sided stroke described by Sperber & Karnath (124). The values being compared reflect the median proportion of voxels in each AAL and JHU atlas area that are included in the lesion.

Max – Maximum; Min – Minimum; NA – Not Applicable; n.s. – non-significant;

This exploratory analysis only considers patients with no personal or family history of psychiatric disorder, with vascular lesion etiology, that fulfill DSM 5 criteria for manic episode, and with CT or MRI images for analysis (i.e. all the group diagrams were excluded). Due to the exploratory nature of these analyses, and the low number of patients, analyses were not corrected for multiple comparisons.

**
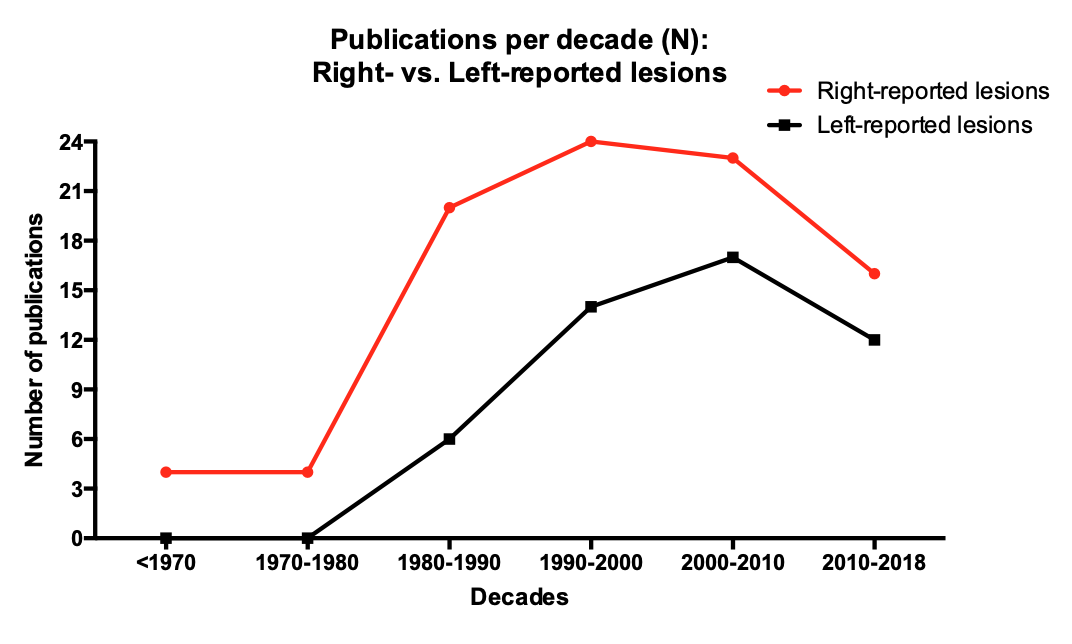
Figure S1 –** Number and percentage of publications per decade reporting left- vs. right-sided lesions

Across time, the absolute number of publications with lesional mania cases reporting right-sided lesions was always higher than those reporting left-sided lesions. Publications reporting bilateral focal lesions are considered both for right-sided and left-sided lesions.

**Figure S2 –** Lesion distribution by major brain areas in cases meeting DSM5 manic syndrome (A), after excluding surgical etiology (B) and with no medication at mania onset

**
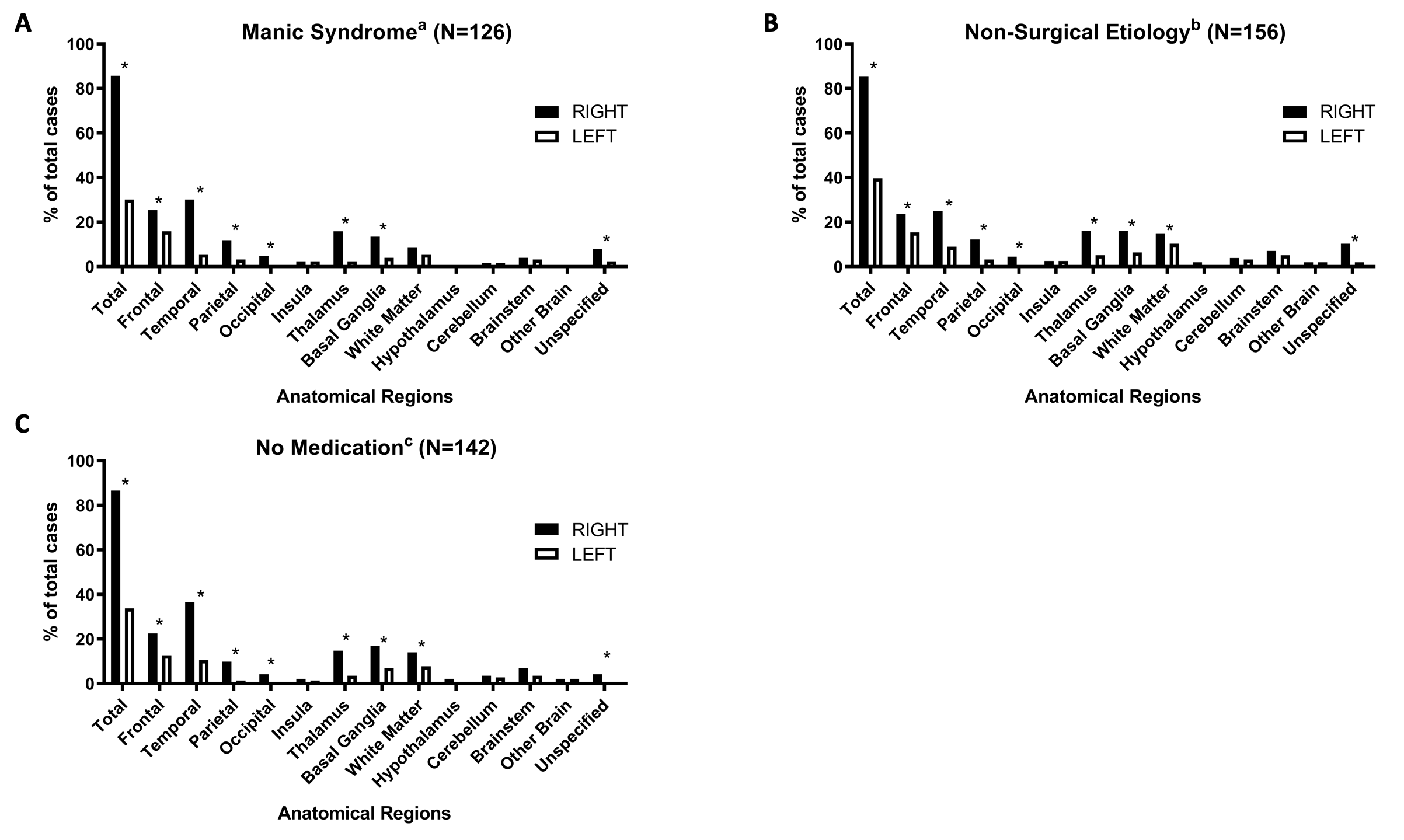
**

^a^ Lesion distribution for cases who met criteria for manic syndrome according to DSM5

^b^ Does not include the following case series, which did not provide enough information on individual lesion etiology: Starkstein 1987 and first case series of Starkstein 1991 (See Table S3 for complete references).

^c^ Does not include the following case series, which did not provide enough information on individual medication report: Robinson 1988 and Starkstein 1987 (See Table S3 for complete references).

**References for Supplementary Material**

1. Wells G, et al. Newcastle-Ottawa quality assessment scale cohort studies. 2014.

2. Gagnier JJ, et al. The CARE guidelines: consensus-based clinical case report guideline development. Journal of clinical epidemiology. 2014;67(1):46-51.

3. Murad MH, Mustafa RA, Schünemann HJ, Sultan S, Santesso N. Rating the certainty in evidence in the absence of a single estimate of effect. BMJ Evidence-Based Medicine. 2017;22(3):85-7.

4. Moga C, Guo B, Schopflocher D, Harstall C. Development of a quality appraisal tool for case series studies using a modified Delphi technique. Edmonton AB: Institute of Health Economics. 2012.

5. Šimundić A-M. Measures of diagnostic accuracy: basic definitions. Ejifcc. 2009;19(4):203.

6. Rice ME, Harris GT. Comparing effect sizes in follow-up studies: ROC Area, Cohen's d, and r. Law and human behavior. 2005;29(5):615-20.

7. Fleiss JL. Measuring nominal scale agreement among many raters. Psychological bulletin. 1971;76(5):378.

8. Gwet KL. Computing inter‐rater reliability and its variance in the presence of high agreement. British Journal of Mathematical and Statistical Psychology. 2008;61(1):29-48.

9. Alla P, de Jaureguiberry JP, Galzin M, Gisserot O, Jaubert D. Hemiballism with manic access caused by toxoplasmic abscess in AIDS. Annales de medecine interne. 1997;148(7):507-9.

10. Alpers BJ. Relation of the hypothalamus to disorders of personality: report of a case. Archives of Neurology & Psychiatry. 1937;38(2):291-303.

11. Antelmi E, Fabbri M, Cretella L, Guarino M, Stracciari A. Late onset bipolar disorder due to a lacunar state. Behavioural neurology. 2014;2014:780742.

12. Asghar-Ali AA, Taber KH, Hurley RA, Hayman LA. Pure neuropsychiatric presentation of multiple sclerosis. 2014.

13. Avery T. Seven cases of frontal tumour with psychiatric presentation. The British Journal of Psychiatry. 1971;119(548):19-23.

14. Bakchine S, et al. Manic-like state after bilateral orbitofrontal and right temporoparietal injury: efficacy of clonidine. Neurology. 1989;39(6):777-81.

15. Bamrah JS, Johnson J. Bipolar affective disorder following head injury. The British journal of psychiatry : the journal of mental science. 1991;158:117-9.

16. Barczak P, Edmunds E, Betts T. Hypomania following complex partial seizures. A report of three cases. The British Journal of Psychiatry. 1988;152(1):137-9.

17. Belli H, Akbudak M, Ural C, Kulacaoglu F. Solitary lesion in ponto-mesencephalic area related secondary mania: a case report. Psychiatria Danubina. 2012;24(2):223-5.

18. Bengesser SA, et al. Poststroke-bipolar affective disorder. Fortschritte der Neurologie-Psychiatrie. 2013;81(8):459-63.

19. Benjamin S, Kirsch D, Visscher T, Ozbayrak KR, Weaver JP. Hypomania from left frontal AVM resection. Neurology. 2000;54(6):1389-90.

20. Benke T, Kurzthaler I, Schmidauer C, Moncayo R, Donnemiller E. Mania caused by a diencephalic lesion. Neuropsychologia. 2002;40(3):245-52.

21. Berthier M. Post-stroke rapid cycling bipolar affective disorder. The British Journal of Psychiatry. 1992;160(2):283-.

22. Berthier ML, Kulisevsky J, Gironell A, Fernandez Benitez JA. Poststroke bipolar affective disorder: clinical subtypes, concurrent movement disorders, and anatomical correlates. The Journal of neuropsychiatry and clinical neurosciences. 1996;8(2):160-7.

23. Bhanji S, Gardner-Thorpe C, Rahavard F. Aqueduct stenosis and manic depressive psychosis. Journal of neurology, neurosurgery, and psychiatry. 1983;46(12):1158-9.

24. Bhatia MS, Srivastava S, Jhanjee A, Oberoi A. Colloid cyst presenting as recurrent mania. The Journal of neuropsychiatry and clinical neurosciences. 2013;25(3):E01-2.

25. Binder RL. Neurologically silent brain tumors in psychiatric hospital admissions: three cases and a review. The Journal of clinical psychiatry. 1983;44(3):94-7.

26. Bobo WV, Murphy MJ, Heckers SH. Recurring episodes of Bell's mania after cerebrovascular accident. Psychosomatics. 2009;50(3):285-8.

27. Bogousslavsky J, et al. Manic delirium and frontal-like syndrome with paramedian infarction of the right thalamus. Journal of neurology, neurosurgery, and psychiatry. 1988;51(1):116-9.

28. Bornke C, Postert T, Przuntek H, Buttner T. Acute mania due to a right hemisphere infarction. European Journal of Neurology. 1998;5(4):407-9.

29. Brooks JO, 3rd, Hoblyn JC. Secondary mania in older adults. The American journal of psychiatry. 2005;162(11):2033-8.

30. Caeiro L, Ferro J, Albuquerque R, Figueira M. Mania no AVC agudo. Sinapse. 2002;2:90.

31. Caeiro L, Santos CO, Ferro JM, Figueira ML. Neuropsychiatric disturbances in acute subarachnoid haemorrhage. European Journal of Neurology. 2011;18(6):857-64.

32. Calo JJP, et al. Mania after traumatic brain injury - a report of 2 cases and 194 literature-review. Archivos De Neurobiologia. 1994;57(4):194-201.

33. Camden JR, Spiegel DR. Manic behavior resulting from left frontal closed head injury in an adult with fetal alcohol syndrome. Psychosomatics. 2007;48(5):433-5.

34. Carran MA, Kohler CG, O'Connor MJ, Bilker WB, Sperling MR. Mania following temporal lobectomy. Neurology. 2003;61(6):770-4.

35. Celik Y, Erdogan E, Tuglu C, Utku U. Post-stroke mania in late life due to right temporoparietal infarction. Psychiatry and clinical neurosciences. 2004;58(4):446-7.

36. Chimowitz M, Furlan A. Resolution of psychotic depression after right temporoparietal infarction. J Nerv Ment Dis 1990;178::458-9.

37. Clark AF, Davison K. Mania following head injury. A report of two cases and a review of the literature. The British journal of psychiatry : the journal of mental science. 1987;150:841-4.

38. Claude H, Baruk H, Lamache A, Cuel J. Manic excitation and cerebral tumor. Encephale-Revue De Psychiatrie Clinique Biologique Et Therapeutique. 1928;23(1):9-19.

39. Cohen MR, Niska RW. Localized right cerebral hemisphere dysfunction and recurrent mania. The American journal of psychiatry. 1980;137(7):847-8.

40. Danel T, et al. Mood disorders and right hemisphere infarction. L'Encephale. 1989;15(6):549-53.

41. Daniels JP, Felde A. Quetiapine treatment for mania secondary to brain injury in 2 patients. The Journal of clinical psychiatry. 2008;69(3):497-8.

42. Das P, Chopra A, Rai A, Kuppuswamy PS. Late-onset recurrent mania as a manifestation of Wallenberg syndrome: a case report and review of the literature. Bipolar disorders. 2015;17(6):677-82.

43. Dauncey K. Mania in the early stages of AIDS. The British Journal of Psychiatry. 1988;152(5):716-7.

44. Drake ME, Jr., Pakalnis A, Phillips B. Secondary mania after ventral pontine infarction. The Journal of neuropsychiatry and clinical neurosciences. 1990;2(3):322-5.

45. El Hechmi S, Ben Romdhane I, Belkhiria A, Medini F, Labbene R. Bipolar disorder in the aftermath of a traumatic brain injury: report of a case. European Psychiatry. 2013;28(8):54-.

46. Estrade JF, Samuel-Lajeunesse B. Secondary mania. Diagnostic problems (apropos of a case of secondary mania in partial complex epilepsy crisis). Annales medico-psychologiques. 1989;147(6):662-7.

47. Fawcett RG. Cerebral infarct presenting as mania. The Journal of clinical psychiatry. 1991;52(8):352-3.

48. Fenn D, George K. Post-stroke mania late in life involving the left hemisphere. Australian and New Zealand Journal of Psychiatry. 1999;33(4):598-600.

49. Filley CM, Kleinschmidt-DeMasters BK. Neurobehavioral presentations of brain neoplasms. The Western journal of medicine. 1995;163(1):19-25.

50. Gafoor R, O'Keane V. Three case reports of secondary mania: evidence supporting a right frontotemporal locus. European Psychiatry. 2003;18(1):32-3.

51. Gal P. Mental symptoms in cases of tumor of temporal lobe. American Journal of Psychiatry. 1958;115(2):157-60.

52. Galindo Menendez A. Parenchymal neurosyphilis. Insidious onset (dementia) and acute onset (manic type) forms. Actas luso-espanolas de neurologia, psiquiatria y ciencias afines. 1996;24(5):261-7.

53. Garland EJ, Zis AP. Multiple-sclerosis and affective-disorders. Canadian Journal of Psychiatry-Revue Canadienne De Psychiatrie. 1991;36(2):112-7.

54. Goyal R, Sameer M, Chandrasekaran R. Mania secondary to right-sided stroke-responsive to olanzapine. General hospital psychiatry. 2006;28(3):262-3.

55. Greenberg DB, Brown GL. Mania resulting from brain stem tumor. J Nerv Ment Dis. 1985;173(7):434-6.

56. Haq MZ, Dubey I, Khess CR, Das U, Kumar R. Bipolar disorder and tuberous sclerosis complex: is it a mere coincidence? CNS spectrums. 2009;14(11):643-7.

57. Heinrich TW, Junig JT. Recurrent mania associated with repeated brain injury. General Hospital Psychiatry. 2004;26(6):490-2.

58. Huffman J, Stern TA. Acute psychiatric manifestations of stroke: a clinical case conference. Psychosomatics. 2003;44(1):65-75.

59. Hunt N, Silverstone T. Seasonal affective disorder following brain injury. The British journal of psychiatry : the journal of mental science. 1990;156:884-6.

60. Inzelberg R, Nisipeanu P, Joel D, Sarkantyus M, Carasso RL. Acute mania and hemichorea. Clinical Neuropharmacology. 2001;24(5):300-3.

61. Isles LJ, Orrell MW. Secondary mania after open-heart-surgery. British Journal of Psychiatry. 1991;159:280-2.

62. Jagadesan V, Thiruvengadam KR, Muralidharan R. Cerebellar Stroke-manifesting as Mania. Indian journal of psychological medicine. 2014;36(3):338-40.

63. Jamieson RC, Wells CE. Manic psychosis in a patient with multiple metastatic brain tumors. The Journal of clinical psychiatry. 1979;40(6):280-3.

64. Jampala VC, Abrams R. Mania secondary to left and right hemisphere damage. The American journal of psychiatry. 1983;140(9):1197-9.

65. Kanemoto K. Hypomania after temporal lobectomy: a sequela to the increased excitability of the residual temporal lobe? Journal of Neurology, Neurosurgery & Psychiatry. 1995;59(4):448-9.

66. Koreki A, Takahata K, Tabuchi H, Kato M. Increased left anterior insular and inferior prefrontal activity in post-stroke mania. BMC neurology. 2012;12:68.

67. Kotrla KJ, Chacko RC, Barrett SA. A case of organic mania associated with open heart surgery. Journal of geriatric psychiatry and neurology. 1994;7(1):8-12.

68. Ku BD, et al. Secondary mania in a patient with delayed anoxic encephalopathy after carbon monoxide intoxication. Journal of clinical neuroscience : official journal of the Neurosurgical Society of Australasia. 2006;13(8):860-2.

69. Kulisevsky J, Berthier ML, Pujol J. Hemiballismus and secondary mania following a right thalamic infarction. Neurology. 1993;43(7):1422-4.

70. Kumar SK, Mahr G. CADASIL presenting as bipolar disorder. Psychosomatics. 1997;38(4):397-8.

71. Lauterbach EC. Bipolar disorders, dystonia, and compulsion after dysfunction of the cerebellum, dentatorubrothalamic tract, and substantia nigra. Biological Psychiatry. 1996;40(8):726-30.

72. Lee YM. Secondary mania in a patient with solitary red nucleus lesion. Psychiatry and clinical neurosciences. 2014;68(3):243.

73. Leibson E. Anosognosia and mania associated with right thalamic haemorrhage. Journal of neurology, neurosurgery, and psychiatry. 2000;68(1):107-8.

74. Liu CY, Wang SJ, Fuh JL, Yang YY, Liu HC. Bipolar disorder following a stroke involving the left hemisphere. Australian and New Zealand Journal of Psychiatry. 1996;30(5):688-91.

75. Malamud N. Psychiatric disorder with intracranial tumors of limbic system. Archives of Neurology. 1967;17(2):113-23.

76. Mark M, Modai I, Aizenberg D, Heilbronn Y, Elizur A. Bipolar disorder associated with an acoustic neurinoma. Psychiatric Services. 1991;42(12):1258-60.

77. McKeown SP, Jani CJ. Mania following head injury. The British journal of psychiatry : the journal of mental science. 1987;151:867-8.

78. Miller BL, Cummings JL, McIntyre H, Ebers G, Grode M. Hypersexuality or altered sexual preference following brain injury. Journal of neurology, neurosurgery, and psychiatry. 1986;49(8):867-73.

79. Modrego PJ, Ferrandez J. Familial multiple sclerosis with repetitive relapses of manic psychosis in two patients (mother and daughter). Behavioural Neurology. 2000;12(4):175-9.

80. Mumoli N, Pulera F, Vitale J, Camaiti A. Frontal lobe syndrome caused by a giant meningioma presenting as depression and bipolar disorder. Singapore medical journal. 2013;54(8):e158-9.

81. Murai T, Fujimoto S. Rapid cycling bipolar disorder after left temporal polar damage. Brain Injury. 2003;17(4):355-8.

82. Mustafa B, Evrim O, Sari A. Secondary mania following traumatic brain injury. Journal of Neuropsychiatry and Clinical Neurosciences. 2005;17(1):122-4.

83. Nagaratnam N, Tse A, Lim R, Chowdhury M. Aberrant sexual behaviour following stroke. European Journal Of Internal Medicine. 1998;9:207-10.

84. Nagaratnam N, Wong KK, Patel I. Secondary mania of vascular origin in elderly patients: a report of two clinical cases. Archives of gerontology and geriatrics. 2006;43(2):223-32.

85. Nizamie SH, Nizamie A, Borde M, Sharma S. Mania following head injury: case reports and neuropsychological findings. Acta psychiatrica Scandinavica. 1988;77(6):637-9.

86. Koh OH, Azreen HN, Gill JS, Pillai SK. A case of post-stroke mania. Malays. J. Psychiatry. 2010;19(1):41-45.

87. Okun MS, Bakay RA, DeLong MR, Vitek JL. Transient manic behavior after pallidotomy. Brain and cognition. 2003;52(2):281-3.

88. Oppler W. Manic psychosis in a case of parasagittal meningioma. Archives of Neurology & Psychiatry. 1950;64(3):417-30.

89. Park S, Park B, Koh MK, Joo YH. Case report: bipolar disorder as the first manifestation of CADASIL. Bmc Psychiatry. 2014;14.

90. Pathak A, Srivastava M. Post-stroke mania–a case report. ASEAN Journal of Psychiatry. 2014;15(2):209-12.

91. Reisch T, Brekenfeld C, Barth A. A case of hydrocephalus occlusus presenting as bipolar disorder. Acta psychiatrica Scandinavica. 2005;112(2):159-62.

92. Robinson RG, Boston JD, Starkstein SE, Price TR. Comparison of mania and depression after brain injury: causal factors. The American journal of psychiatry. 1988;145(2):172-8.

93. Robinson RG, Kubos KL, Starr L, Rao K, Price T. Mood disorders in stroke patients. Brain. 1984;107(Pt 1):81-93.

94. Rocha FF, Carneiro JG, Pereira Pde A, Correa H, Teixeira AL. Poststroke manic symptoms: an unusual neuropsychiatric condition. Revista brasileira de psiquiatria (Sao Paulo, Brazil : 1999). 2008;30(2):173-4.

95. Rocha FF, Correa H, Teixeira AL. A successful outcome with valproic acid in a case of mania secondary to stroke of the right frontal lobe. Progress in neuro-psychopharmacology & biological psychiatry. 2008;32(2):587-8.

96. Rosenbaum AH, Barry MJ, Jr. Positive therapeutic response to lithium in hypomania secondary to organic brain syndrome. The American journal of psychiatry. 1975;132(10):1072-3.

97. Routh R, Hill A. Post‐stroke mania: a rare but treatable presentation. Progress in Neurology and Psychiatry. 2014;18(1):24-5.

98. Salazar-Calderon Perriggo VH, Oommen KJ, Sobonya RE. Silent solitary right parietal chondroma resulting in secondary mania. Clinical neuropathology. 1993;12(6):325-9.

99. Sanders RD, Mathews TA. Hypergraphia and Secondary Mania in Temporal Lobe Epilepsy: Case Reports and Literature Review. Cognitive and Behavioral Neurology. 1994;7(2):114-7.

100. Semiz M, Kavakci O, Yontar G, Yildirim O. Case of organic mania associated with stroke and open heart surgery. Psychiatry and clinical neurosciences. 2010;64(5):587.

101. Semiz UB, et al. Leptospirosis presenting with mania and psychosis: Four consecutive cases seen in a military hospital in Turkey. International Journal of Psychiatry in Medicine. 2005;35(3):299-305.

102. Sidhom Y, et al. Bipolar Disorder and Multiple Sclerosis: A Case Series. Behavioural Neurology. 2014.

103. Starkstein SE, Boston JD, Robinson RG. Mechanisms of mania after brain injury. 12 case reports and review of the literature. J Nerv Ment Dis. 1988;176(2):87-100.

104. Starkstein SE, Fedoroff P, Berthier ML, Robinson RG. Manic-depressive and pure manic states after brain lesions. Biological psychiatry. 1991;29(2):149-58.

105. Starkstein SE, et al. Mania after brain injury: neuroradiological and metabolic findings. Annals of neurology. 1990;27(6):652-9.

106. Starkstein SE, Pearlson GD, Boston J, Robinson RG. Mania after brain injury. A controlled study of causative factors. Archives of neurology. 1987;44(10):1069-73.

107. Stern K, Dancey TE. Glioma of the diencephalon in a manic patient. American Journal of Psychiatry. 1942;98(5):716-9.

108. Sullivan G, Jenkins PL. Secondary mania following cerebral hypoxia. Irish Journal of Psychological Medicine. 1995;12(2):68-9.

109. Sweet RA. Case of craniopharyngioma in late life. The Journal of neuropsychiatry and clinical neurosciences. 1990.

110. Topcuoglu V, Gimzal Gonentur A, Bilgin Topcuoglu O, Yazgan C, Kora K. Mood Disorder due to Herpes Simplex Encephalitis with Neuroimaging Findings Limited to the Right Hemisphere and Cerebellum: Case Report. Turkiye Klinikleri Tip Bilimleri Dergisi. 2012;32(6):1724-8.

111. Trillet M, Vighetto A, Croisile B, Charles N, Aimard G. Hemiballismus with logorrhea and thymo-affective disinhibition caused by hematoma of the left subthalamic nucleus. Revue neurologique. 1995;151(6-7):416-9.

112. Trimble MR, Cummings JL. Neuropsychiatric disturbances following brainstem lesions. The British Journal of Psychiatry. 1981;138(1):56-9.

113. Turecki G, Mari Jde J, Del Porto JA. Bipolar disorder following a left basal-ganglia stroke. The British journal of psychiatry : the journal of mental science. 1993;163:690.

114. Vidrih B, Karlovic D, Pasic MB. Arachnoid cyst as the cause of bipolar affective disorder: case report. Acta clinica Croatica. 2012;51(4):655-9.

115. Wright MT, Cummings JL, Mendez MF, Foti DJ. Bipolar syndromes following brain trauma. Neurocase. 1997;3(2):111-8.

116. Ybarra MI, Moreira MA, Araujo CR, Lana-Peixoto MA, Lucio Teixeira A. Bipolar disorder and multiple sclerosis. Arquivos De Neuro-Psiquiatria. 2007;65(4B):1177-80.

117. Yetimalar Y, Iyidogan E, Basoglu M. Secondary mania after pontin cavernous angioma. The Journal of neuropsychiatry and clinical neurosciences. 2007;19(3):344-5.

118. Zincir SB, Izci F, Acar G. Mania secondary to traumatic brain injury: a case report. The Journal of neuropsychiatry and clinical neurosciences. 2014;26(2):E31.

119. Julayanont P, Ruthirago D, Alam K, Alderazi YJ. Behavioral Disconnection Syndrome Manifesting as Combined Mania and Visual-Auditory Hallucinations Secondary to Isolated Right Thalamic Hemorrhage. Journal of Neuropsychiatry and Clinical Neurosciences. 2017;29(4):401-8.

120. Kar SK, Das KK, Jaiswal AK, Jaiswal S. Mood Disorder as an Early Presentation of Epidermoid of Quadrigeminal Cistern. Journal of Neurosciences in Rural Practice. 2017;8(3):443-5.

121. Lupo M, et al. Evidence of Cerebellar Involvement in the Onset of a Manic State. Frontiers in Neurology. 2018;9.

122. Taylor JB, Prager LM, Quijije NV, Schaefer PW. Case 21-2018: A 61-Year-Old Man with Grandiosity, Impulsivity, and Decreased Sleep. N Engl J Med. 2018;379(2):182-9.

123. Benjamini Y, Hochberg Y. Controlling the false discovery rate: a practical and powerful approach to multiple testing. Journal of the royal statistical society Series B (Methodological). 1995:289-300.

124. Sperber C, Karnath H-O. Topography of acute stroke in a sample of 439 right brain damaged patients. NeuroImage: Clinical. 2016;10:124-8.
