# Supplementary material for: Right-sided brain lesions predominate among patients with lesional mania: evidence from a systematic review and pooled lesion analysis
Source: Transl Psychiatry. 2020 May 12;10:139. doi: 10.1038/s41398-020-0811-0 (PMC7217919; doi:10.1038/s41398-020-0811-0)
Supplement: Supplementary file 2 — Matlab Script [file 41398_2020_811_MOESM2_ESM.pdf]

```
% Lisboa re-analysis 2020: transforming 3D lesions into sliced images for
% comparability with the post stroke bipolar cases taken from the
% literature
clear
clc

z_extent=zeros(492,1);

for i=1:492
    try
    header=spm_vol(['D:\Abgeschlossene
Projekte\Project_Stroke_Anatomy_03112015\atlas_analysis_lisboa\lesions\RH
LM_' num2str(1000+i) '.nii']);
    lesion=spm_read_vols(header);
    lesion=double(lesion>0);
    lesion_z_sum=squeeze(sum(sum(lesion,1),2));
    z_extent(i)=sum(lesion_z_sum>0);
    end
end

% required slice numbers according to Bernardo: 212-1 151-2 30-3 16-4 30-
5

% the z-extent values are sorted by size, i.e. larger lesion receive more
% slices
[sorted_z_extent,idx]=sort(z_extent,'descend');
slice_number=zeros(492,1);
slice_number(1:30)=5;
slice_number(31:46)=4;
slice_number(47:76)=3;
slice_number(77:227)=2;
slice_number(228:439)=1;

%slice_number_vector gives for the i-th lesion (RHLM 1000+i) the number
of
%slices

for i=1:492
    slice_number_vector(idx(i))=slice_number(i);
end

%% actual reslicing proces

for i=1:492
try
    header=spm_vol(['D:\Abgeschlossene
Projekte\Project_Stroke_Anatomy_03112015\atlas_analysis_lisboa\lesions\RH
LM_' num2str(1000+i) '.nii']);
    lesion=spm_read_vols(header);
    lesion=double(lesion>0);
    lesion_z_sum=squeeze(sum(sum(lesion,1),2));
    max_z_slice=find(lesion_z_sum==max(lesion_z_sum)); %this identifies
the z-row that contains the maximum lesion load
    max_z_slice = max_z_slice(randi(length(max_z_slice))); % in case the
maximum lesion load is repeated, a random slice of these maximum slices
is chosen
    mask=zeros(181,217,181); %prepare blank for mask
```

```
    if slice_number_vector(i) == 1 % if only 1 slice, then the largest
slice is chosen
        mask(:,:,max_z_slice)=1;
    elseif slice_number_vector(i) == 2 % if 2 slices, then the largest
slice is chosen + 8 slices away the next one, in the z-direction
direction with the larger lesion extent
        mask(:,:,max_z_slice)=1;

        if sum(lesion_z_sum(1:max_z_slice-1)) >
sum(lesion_z_sum(max_z_slice+1:end))
        mask(:,:,max_z_slice-8)=1;
        else
        mask(:,:,max_z_slice+8)=1;
        end

    elseif slice_number_vector(i) == 3 % if 3 slices, then the largest
slice is chosen + 8 slices away the next one above and below
        mask(:,:,max_z_slice)=1;
        mask(:,:,max_z_slice+8)=1;
        mask(:,:,max_z_slice-8)=1;

    elseif slice_number_vector(i) == 4 % if 4 slices, then the largest
slice is chosen + 8 slices away the next one above and below, and 16
slices above/below, whereever the z-lesion-extent is largest
        mask(:,:,max_z_slice)=1;
        mask(:,:,max_z_slice+8)=1;
        mask(:,:,max_z_slice-8)=1;

        if sum(lesion_z_sum(1:max_z_slice-1)) >
sum(lesion_z_sum(max_z_slice+1:end))
        mask(:,:,max_z_slice-16)=1;
        else
        mask(:,:,max_z_slice+16)=1;
        end

    elseif slice_number_vector(i) == 5 % if 6 slices, then the largest
slice is chosen + 8 and 16 slices away the next ones above and below
        mask(:,:,max_z_slice)=1;
        mask(:,:,max_z_slice+8)=1;
        mask(:,:,max_z_slice-8)=1;
        mask(:,:,max_z_slice+16)=1;
        mask(:,:,max_z_slice-16)=1;
    end

    new_lesion=lesion.*mask;
    header.fname=['D:\Abgeschlossene
Projekte\Coop_secondary_bipolar\Analysis_2020\Sliced_lesions\RHLM_'
num2str(1000+i) '_resliced.nii'];
    spm_write_vol(header,new_lesion);
    end
end
```
